# The evaluation of the effect of probiotics on the healing of equine distal limb wounds

**DOI:** 10.1371/journal.pone.0236761

**Published:** 2020-07-29

**Authors:** Jacintha M. Wilmink, Søren Ladefoged, Angelique Jongbloets, Johannes C. M. Vernooij

**Affiliations:** 1 Woumarec, Wageningen, The Netherlands; 2 Højgård Hestehospital A/S, Morud, Denmark; 3 Paardenkliniek West-Brabant, Roosendaal, The Netherlands; 4 Division Farm Animal Health, Department of Population Health Sciences, Faculty of Veterinary Medicine, University of Utrecht, Utrecht, The Netherlands; Monash University, AUSTRALIA

## Abstract

The effect of dressings saturated with either a standardized suspension of probiotic bacteria or saline on healing of traumatic distal limb wounds in horses was evaluated for 24 days, and the systemic inflammatory effect was assessed. The wounds were divided in two groups based on the phase of healing: wounds with an incomplete (ICGB) or a complete granulation bed (CGB). The wound area was expressed as percentage of the wound area at day 0 and defined as relative wound area. The mean relative wound area decreased faster in probiotic than saline treated wounds. The difference was most obvious in CGB and increased rapidly from day 0 until day 12 up to 30%, and stabilized around 25% thereafter until the end of the observation period, but it was not statistically significant because of the large variation within the treatment groups. The mean wound area of CGB decreased to 28.4% (range: 6.3 to 49.3) with probiotic and to 51.9% (range: 29.3 to 81.7) with saline treatment at day 24. Additionally, the rate to 50% healing in CGB was 3.4 faster with probiotic compared to saline treatment, whereas in ICGB this was 1.9 faster. Topical probiotics did not increase serum amyloid A and white blood cell counts. Although the mentioned differences were not statistically significant, the clinical relevance of the effect of treatment with probiotics in CGB wounds is clear, supported by the differences in mean wound area in course of time and the time required to reach 50% healing (day 12 for probiotic vs more than day 24 for saline treated wounds). Thus the probiotic treated wounds reached 50% reduction in wound area in half of the time of the saline treated wounds. The topical use of probiotics can be considered as safe as it did not cause a systemic effect.

## Introduction

Delayed wound healing is a major issue in both horses and humans. Although the aetiology of chronic wounds in these species is different, striking similarities exist in the pathophysiology and the complications encountered during treatment of wounds healing by second-intention, such as problems with bacterial colonisation and biofilm formation [1, 2], an inappropriate inflammatory response, and unrestrained proliferation resulting in exuberant granulation tissue (EGT) in horses and in hypertrophic scars or keloids in both species [3, 4]. Because of these similarities, the equine wound appears to be a very suitable model for the human chronic wound, and wound studies in horses can give valuable information for possible human application. In both species, bacteria are a continuous threat for wounds healing by second intention. At the same time there is an urge to further reduce the use of antibiotics during wound treatment. Therefore, there is a need to find alternative treatment modalities to limit the influence of pathogenic bacteria that delay wound healing.

In the equine species, traumatic wounds often have to heal by second intention as closure is not possible or not successful [5]. Second-intention healing in horses is slow, particularly when limbs are involved [6]. The wound bed of equine limb wounds is poorly perfused [7], and the initial inflammatory response is weak and progresses into a chronic low-grade response [8], characterized by abundant neutrophil infiltration with release of reactive oxygen species and cytolytic enzymes. Poor perfusion and a weak initial inflammatory response makes equine limb wounds more susceptible to bacterial colonisation and invasion, and to a large extent explains the high incidence of wound dehiscence after closure of horse wounds, as well as the delayed start of second intention healing [8–10]. There is evidence that stimulation of the initial inflammatory response and inhibition of the chronic response improves second intention healing in horses: healing becomes faster and fewer complications are seen [11]. For this reason, it seems interesting to modulate both the bacterial population and the inflammatory response of wounds, which are related to each other and to the speed and efficiency of second intention healing.

Probiotics are defined by the World Health Organisation (WHO) as “Live microorganisms which when administered in adequate amounts confer a health benefit on the host” [12]. Probiotics are non-pathogenic strains of bacteria which have been used orally for various medical applications: such as enteral dysbacteriosis, gastroenteritis, pediatric post-antibiotic-associated diarrhea in humans [13] and diarrhea and colitis in horses [14]. *In vitro* studies have shown that strains of *Lactobacillus* and *Bifidobacterium* are able to inhibit the growth of clinical isolates of *Staphylococcus aureus* and MRSA [15], inhibit elastase and biofilm formation of *Pseudomonas aeruginosa* by affecting the production of quorum-sensing signal molecules [16, 17] and inhibit adhesion and biofilm formation of *Staphylococcus aureus* and *Staphylococcus epidermidis* [18, 19]. Proposed mechanisms of probiotic action include colonization resistance via competition with pathogens for adhesion sites, nutrients and growth factors, modulation of the host immune response and production of various low-molecular-weight substances such as lactic acid and bacteriocins [15, 20, 21].

The topical use of probiotic bacteria for treatment of chronic wounds, e.g. diabetic and non-diabetic foot ulcers and burn wounds has been investigated recently in humans and laboratory rodents. These studies have shown that probiotic bacteria can reduce the bacterial load of wounds, inflammatory cell infiltration, and promote healing [22–25]. However the safety of the use of probiotic bacteria on wounds has not been assessed before, and it is unknown whether these probiotic bacteria can invade wounds and cause a systemic reaction, which may be of importance for immune compromised patients.

The effect of topical treatment of wounds with probiotic bacteria has to our knowledge not been investigated previously in the equine species. Additionally, any systemic reaction after topical application of probiotic bacteria can be easily measured in the equine species because horses react very sensitive to inflammatory insults by rapid induction of the inflammatory marker serum amyloid A [26].

We hypothesized that topical probiotic treatment (1) would stimulate equine wound healing, and (2) would not cause a systemic inflammatory reaction. The effect on equine wounds with an incomplete granulation bed may induce a faster formation of healthy granulation tissue by stimulating the local initial inflammatory response in wounds thus promoting better and faster wound demarcation. The effect in wounds with a complete granulation bed may promote healing by competing with resident pathogenic bacteria.

The aim of the present study was to evaluate the differences in change in wound area in course of time between treatment with probiotic-saturated dressing on distal limb wounds in horses healing by second intention and treatment with saline saturated dressing. We qualitatively scored the wound bed and the horses well-being at each bandage change, as well as the development of bacterial burden between Day 0 and Day 9. Secondly we evaluated as a measure for safety the change in level of serum amyloid A (SAA) in the course of time between both treatments and measured white blood cell count (WBC) and differential blood count as general reference. Both aims may be of interest for both equine and human patients.

## Materials and methods

### Study design

A randomized, single-blinded, multi-centre, clinical study was conducted over a 24-month period including horses with lower limb wounds from six equine referral centres in Denmark (2) and the Netherlands (4). The participating veterinarians were trained by a work-shop or by video.

The horses were randomly assigned to a 24 day treatment period of either topical treatment with probiotic bacteria or control treatment with sterile saline applied to the same type of dressing. Based on a schedule determined beforehand, half of the clinics started with the treatment with probiotic bacteria, the other half with saline, and every next horse per clinic received the other treatment and so on. Informed consent was obtained from all owners prior to inclusion of horses into the study. The study protocol was reviewed and approved prior to the initiation of the study by the Animal Ethics Committee (DEC) and the Animal Welfare Body (IvD) of the Utrecht University, and by the Danish Animal Experimentation Inspectorate, conform the EU standards (European Directive 2010/63/EU).

### Horses

Horses with a limb wound healing by second intention were included in the study, when healthy and having a wither-height of more than 148 cm. For non-adult animals the expected wither-height based on breed should be more than 148 cm. It was decided beforehand that horses with general diseases before the study would not be enrolled, and horses with diseases during the study for more than two days (i.e. colic, fever, allergic reactions) would be excluded. Additionally, horses treated with antimicrobial or immunosuppressive medication, either systemic or topical within seven days before or during the study were excluded. All horses underwent a full clinical examination prior to inclusion in the study. During the study period, the horses were hospitalized and confined to a box stall to create uniform circumstances, fed hay ad libitum and pellets according to assessed daily requirements.

### Wounds

Limb wounds were included when they were at the carpus or tarsus or distally, of more than 10 cm^2^ in size, of less than 6 months’ duration, and without complications such as penetration to synovial cavities. The majority of wounds originated from trauma, and a few from pressure. The wounds were allocated in two groups according to the wound morphology. The group “Incomplete Granulation Bed” (ICGB) included the wounds that had tissue defects or clefts with a depth of more than 0.5 cm below the wound margins, measured with a probe, which had to be filled with granulation tissue. The second group “Complete Granulation Bed” (CGB) included wounds with a complete granulation bed, or only minor clefts or defects of less than 0.5 cm depth. Wounds would be excluded from the wound area measurements when the measurements could not be performed reliably (wounds too wide to record on one photograph) and when abnormalities in the healing pattern occurred, such as an extreme enlargement by any factor, for example trauma (biting, rubbing), necrosis of skin, or wound dehiscence after partial wound closure.

### Wound bed preparation

Horses were restrained if necessary, either by application of a nose twitch or by sedation intravenously with detomidine hydrochloride 0.01–0.03 mg/kg in combination with butorphanol tartrate 0.01–0.02 mg/kg. Wounds were cleaned with sterile saline solution and protected with sterile moistened swabs while the surrounding skin was clipped, washed and flushed with water and thereafter irrigated with saline. Thereafter the swabs were removed, the wounds were irrigated again and then debrided. Initial debridement (day 0) of the wounds in ICGB was as follows: the defect was debrided by using a monofilament polyester debridement pad (Debrisoft, Lohmann & Rauscher, Germany) to remove fibrin and necrosis from the cavity. When additionally, EGT was present, this was excised. EGT was defined as granulation tissue more than 3 mm above the level of the wound margins. In CGB the entire wound surface was excised from distal to proximal. After debridement, the wounds were bandaged with a sterile, non-adherent pressure bandage for 30 minutes to stop bleeding. In case that EGT formed during the study this was excised in the same way.

### Preparation and application of dressing

For the horses assigned to the topical probiotic treatment, a cross-linked acrylate copolymer dressing in fiber form (Oasis SAF, Brightwake Ltd., United Kingdom) was moistened with a probiotic suspension containing strains of *Lactobacillus acidophilus*, *Bifidobacterium animalis* subsp *lactis*, *Lactobacillus paracasei* subsp *paracasei*. Further details about the probiotics are presented in S1 Details. The suspension was provided in 40 ml or 80 ml sealed containers (Biosa Denmark Aps, Denmark) to saturate 10 x 10 cm and 10 x 20 cm dressings respectively. The pH of the probiotic suspension was adjusted prior to use by addition of 10M of sodium hydroxide (NaOH), resulting in total doses of 5x10^6^–10^9^ CFU/ml of pH 5 corresponding to 2x10^6^ - 4x10^8^ CFU/cm^2^. The volume of the solution applied to the fibre dressing and thus the wound was 0.4 ml/cm^2^. The moistened dressing was cut in the shape of the wound, placed on the wound and covered by two dry layers of the fibre dressing that overlapped the wound with saturated dressing in all directions. The entire procedure was sterile. A secondary layer of padding (Gamgee, Robinson Healthcare Ltd., United Kingdom) was applied and the bandage was finished with a third layer of elastic self-adhesive bandage. For the horses assigned to the control treatment, the same type of primary cross-linked acrylate copolymer dressing was moistened with 0.4 ml/cm^2^ sterile saline, cut in shape and placed on the wound; the rest of the procedure was identical to the probiotic treatment.

### Wound evaluation

The dressings and bandages were changed and wounds evaluated at day 0, 1, 3, 6, 9, 12, 15, 18, 21 and day 24 of the study. Wounds were gently cleaned with swabs moistened with sterile saline during which exudate and debris was removed. At each bandage change 4 standardized digital photographs (Samsung ST66 with image size set at 3 Megapixel, Samsung Electronics Ltd., United Kingdom) were taken from a distance of 40 cm perpendicular to the wound with rulers held vertically and horizontally adjacent to the wound as a reference. The best photograph was selected. Area analysis of the digitally imaged wounds and reference calibration were performed by one person unacquainted with the horses and their treatments, using image analysis software (ImageJ version 1.47, National Institutes of Health, USA). The total wound area, average of 3 measurements, was determined in cm^2^. To enable comparison of wounds of different sizes, the wound area was expressed as percentage of the wound area at Day 0 and named the ‘relative wound area’. Thus, the relative wound area at day *x* was defined as the wound area (cm^2^) measured at day *x* expressed as a percentage of the wound area (cm^2^) measured at Day 0.

Following criteria were scored (0 = not present, 1 = minimal, 2 = slight, 3 = moderate, 4 = marked) at each bandage: signs of pain, lameness at the walk, amount of wound exudate, presence of malodour, problems of the surrounding skin and EGT formation. Additionally, the wound bed was scored as regular or irregular when defects of less respectively more than 0.5 cm were present, the depth of any defects of the wound bed was assessed in mm, and excision of EGT was recorded.

### Hematology

On day 0, 3 and 9 a blood sample was drawn from the jugular vein into sodium-EDTA tubes for determination of white blood cell count (WBC) and serum tubes containing no additive for preparation of serum samples for analysis of serum amyloid A (SAA). WBC and differential blood count were determined as generally known reference for a systemic inflammatory reaction. They were obtained using an automated flow cytometric hematology system (ADVIA 120 system, Siemens Healthcare, Germany). The blood samples in the serum tubes were allowed to coagulate for approximately 30 minutes before centrifugation at 2500 *g* for 5 minutes after which serum was collected, and stored in a cryo-tube at -20˚C until analysis. SAA protein concentrations were determined using a commercial available latex agglutination immunoassay (Eiken Chemical Co., Tokyo, Japan), previously validated for use in horses [27]. Values outside the reference range were marked.

### Wound swabs

On day 0, 3 and 9 a wound swab was taken for identification of aerobic bacteria. A sterile cotton tipped swab was rolled over the entire wound surface for at least 5 seconds. The swabs were placed in Amies transport medium (Sarstedt AG & Co, Germany), stored at 4^ᵒ^C, processed within 24–96 hours by Laboklin GMBH & CO. KG., Germany, and analysed according to standard microbiology culture protocol. In short, each swab was smeared onto a Colombia agar plate with 5% sheep blood (bioMérieux, France) and an Endo-Agar plate (BD, United States) via a fractionated smear of 3 fractions. Then the swab was placed in an enrichment bouillon (DifcoTM Fluid Thioglycolate Medium, BD, United States). The agar plates and bouillon were incubated for 18 +/- 24 hours at 36 +/- 2°C. After incubation the enrichment bouillon was smeared out in the same way on both types of plates and incubated as stated above. The original agar plates were analysed on bacteria species and quantity of the colonies that were grown. The bacteria species were determined by culture morphology, native or Gram staining, biochemical methods or Matrix-assisted laser Desorption-Ionization-Time of flight (Maldi-Tof) mass spectrometry (Laboklin GMBH & CO. KG., Germany), and the number of species per wound was counted. For quantification, distinction was made between low (growth in the first fraction, score 1), moderate (growth in the second fraction, score 2) and high (growth in the third fraction, score 3) concentration. Total score per wound was determined by adding the scores per present bacterial species.

### Statistical analysis

A full description of the data for the analysis is presented in S1 Data. Differences between means of relative wound area reduction (%) between groups were assessed using linear mixed effects analysis. Survival analysis was performed to assess the hazard ratio for the time between start of the treatment and the day reaching 50% healing of the wounds between the groups. A proportional odds model with random effects was applied to analyze the qualitative scores of the wound bed in course of time between the groups.

The linear mixed effects model [28] was applied with percentage wound area as outcome variable with explanatory variables treatment, day, wound group and 2-way and 3-way interactions between the explanatory variables. The validity of the model (normality and constant variance) was assessed by the visual inspection of residual plots. Horse ID and time within horse were added to the model to estimate the random intercept and a random slope within horse. As the wound area per time point was relative (percentage) to the area at intake the values at day 0 were excluded as they did not show variation (all 100%).

Secondly a cox proportional hazards analysis [29] was applied with time between start of the treatment and the day reaching 50% reduction in wound area as outcome with explanatory variables wound group, treatment and the interaction between both. The proportional hazards assumption was assessed by visual inspection of the residual plots which was met for the comparison between both treatments within ICGB and CGB respectively.

For both the linear mixed model and the cox proportional hazards analysis the Akaike’s Information criterion (AIC) model selection criterion (smaller is better) was used to select the best model between competing models using a backward selection approach. The estimated effect size with 95% confidence interval (CI) were presented for probiotic versus control treatment, according to the REFLECT guidelines for reporting randomized controlled trials [30].

The improvement (lower number) between day 0 and day 9 in number of bacterial spp and qualitative score for bacterial load respectively between groups was tested by estimating the odds ratio and by fisher’s exact test. The analysis was conducted in R version 3.3.0 [31].

Differences in the qualitatively scored outcomes (pain, lameness at walk, problems with surrounding skin, amount of exudate, presence of malodour, and EGT formation) between groups were tested using repeated measurement analysis based on the proportional odds model. The level of significance was set at 5%.

## Results

### Horses

Twenty-nine warmblood horses were included in the study. The wounds of 15 horses were treated with probiotics and those of the other 14 horses were treated with saline. The horses were healthy at the start of the study and, apart from one horse that showed signs of upper respiratory tract infection at day 9 which resolved without treatment, the horses remained healthy during the study period without using any antimicrobial or immunosuppressive medication. There were no exclusions because of medication during the study. At least 50% of the reports showed that horses had received systemic and/or topical antimicrobial and/or immunosuppressive medication during the treatment period before the start of the study (9 probiotic, 7 saline). Seven horses (3 probiotic, 4 saline), treated with antimicrobials and NSAIDs just before entering the clinic, were included when the animals were free of medication for at least 7 days. Patient demographics and baseline wound recordings were not different between the groups (Table 1).

**Table 1:** Patient demographics and baseline wound recordings of the 29 warmblood horses included in the study.

Parameter	Probiotic dressing	Saline dressing
**Number of patients (N)**	15	14
**Age (months) (mean, range)**	65 (4–192)	63 (1–204)
**Gender (female/male)**	9/6	8/6
**Horse height (cm) (mean, range)**	160 (148–172)	161 (120–175)
**Horse weight (kg) (mean, range)**	464 (250–600)	464 (150–750)
**Body condition (below average/average/above average)**	1/14/0	2/12/0
**Baseline wound size (cm**^**2**^**) (mean, range)**	37.7 (10.7–94.4)	36.9 (11.3–107.3)
**ICGB (mean, range)**	29.2 (13.5–62.9)	33.7 (11.3–107.3)
**CGB (mean, range)**	47.4 (10.7–94.4)	41.2 (16.0–59.9)
**Wound age at start of study (days) (mean, range)**	24 (7–56)	23 (0–60)
**ICGB (mean, range)**	14 (7–35)	16 (0–42)
**CGB (mean, range)**	34 (14–56)	33 (14–60
**Wound with and without a defect (ICGB/CGB)**	8/7	8/6
**Origin of wound (trauma/pressure ulcer/unknown)**	12/1/2	12/1/1

ICGB = incomplete granulation bed; CGB = complete granulation bed.

The wounds of 4 horses (1 treated with probiotics and 3 treated with saline) were excluded from the wound area analysis for the following reasons. The wound of one horse (ICGB, saline treatment) had a skin flap that became necrotic and was excised at day 3. The wound of another horse (ICGB, saline treatment) was partially sutured but wound dehiscence occurred after day 7. For both wounds this meant an immediate increase in the wound areas to 165 and 300% respectively relative to day 0 making the curves useless for wound area measurements. The wounds of two other horses (ICGB, saline treatment; CGB, probiotic treatment) were too wide to record in one photograph impeding reliable measurements and calculations of the wound areas. The data of these 4 horses and wounds were included in the analysis except for the evaluation of the wound area.

### Wounds

**Fig 1:**
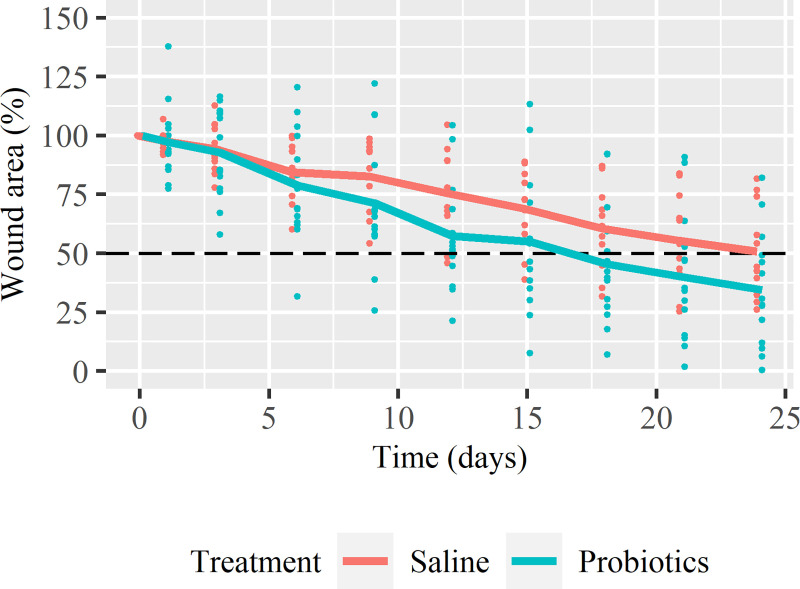
The relative wound area as a function of time. The relative wound area at day *x* is defined as the wound area measured at day *x* (cm^2^) expressed as a percentage of the wound area measured at day 0 (cm^2^). The dots represent the data of the individual wounds and the lines represent the mean relative wound area of all horses per treatment. The dashed line (- -) indicates the level of 50% wound repair. Data points of both groups are shifted a little for better visualization.

**Fig 2:**
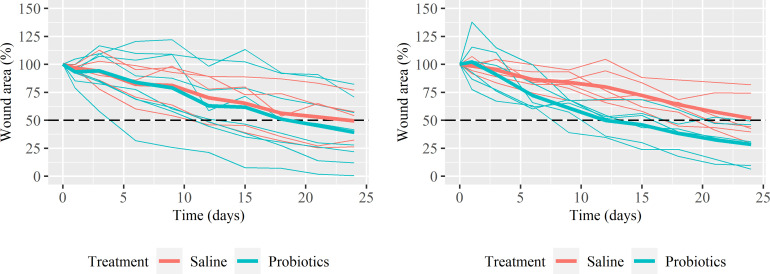
The relative wound area of the wounds as a function of time. Fig 2A represents wounds with Incomplete Granulation Bed (ICGB) and Fig 2B for Complete Granulation Bed (CGB). The thin lines represent the individual wounds and the bold lines represent the mean relative wound area per treatment. The dashed line (- -) indicates the level of 50% wound repair. Missing data are estimated by the mean of the previous and following measurement within horse.

The wound area (% of the initial wound area) of both the probiotic and saline treatment show a gradual decrease in course of time (Fig 1). The mean wound area of ICGB decreased to 39.1% (range: 0.4 to 82.1) with probiotic and to 49.4% (range: 26.2 to 76.8) with saline treatment after 24 days. Half of the ICGB wounds enlarged initially, before decreasing in size, irrespective of treatment (Fig 2A). The mean wound area of CGB decreased to 28.4% (range: 6.3 to 49.3) with probiotic and to 51.9% (range: 29.3 to 81.7) with saline treatment after 24 days (Fig 2B). CGB showed mainly a decrease in size from the beginning (Fig 2B), with only some enlargement as reaction on the debridement. The variation in the level of the healing curves is larger in ICGB compared to CGB.

The estimated effect sizes with 95% confidential intervals are presented in Table 2. The mean decrease in wound area of ICGB with probiotic treatment was very similar to the decrease of the mean wound area in the control group as the difference between the means is close to 0. The estimated mean percentage wound area with probiotic treatment in CGB was more than 20 percentage points smaller from day 9 onwards compared to the wound area in the saline treatment group (Table 2). The difference increased rapidly from the start of the study until day 12 up to 30 percentage points, and stabilized around a difference of 25 percentage points till the end of the observation period, when selecting the best statistical model only Time remained in the model.

**Table 2:** Estimates and 95% confidence intervals of the difference between the mean % of wound area of probiotic treated and saline treated wounds of ICGB and CGB respectively in course of time.

	ICGB			CGB		
	Wounds with defect in granulation bed			Wounds with intact granulation bed		
Day^1^	Estimate^2^	Confidence Interval		Estimate^2^	Confidence Interval	
		2.5%	97.5%		2.5%	97.5%
**1**	-4.6	-22.4	13.3	4.9	-13.1	22.9
**3**	0.3	-17.1	17.6	-4.1	-21.7	13.5
**6**	1.7	-16.3	19.8	-13.7	-32.2	4.7
**9**	-2.2	-21.4	17.0	-22.8	-42.3	-3.4
**12**	-7.2	-28.0	13.5	-30.0	-51.0	-9.0
**15**	-3.5	-26.0	19.1	-26.2	-49.0	-3.3
**18**	-5.3	-29.9	19.3	-25.9	-51.0	-0.8
**21**	-7.7	-34.6	19.2	-24.8	-52.2	2.6
**24**	-10.3	-39.6	19.0	-23.5	-53.1	6.1

The results are from the most extended model nevertheless the model including only Day was the best model based on the AIC.

^1^ Days after the start of treatment.

^2^ Estimated difference between mean_probiotic_ and mean_saline_ at each day.

**Fig 3:**
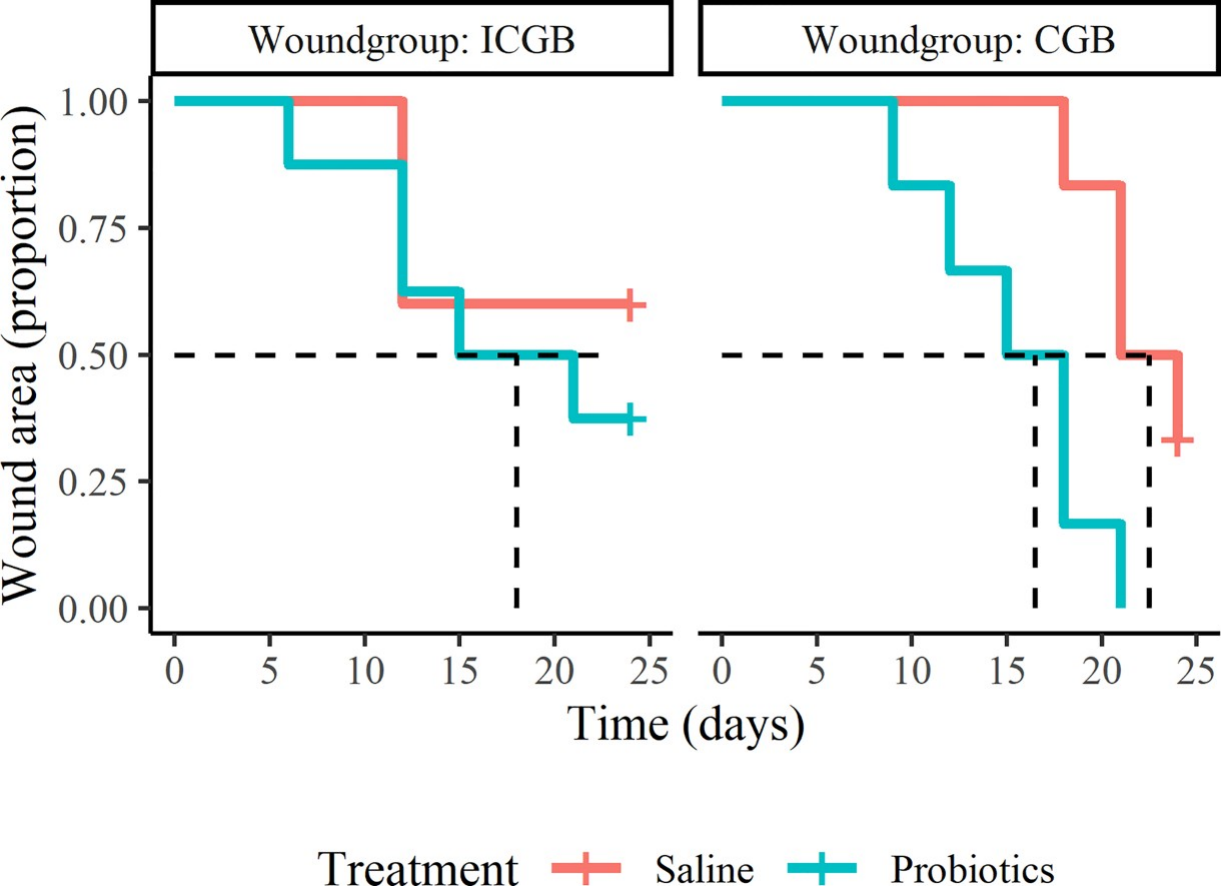
Kaplan-Meier graph showing the proportion of wounds with a wound area of more than 50 percent of the initial wound area. ICGB = group with incomplete granulation bed, CGB = group with complete granulation bed. The dashed line (- -) indicates the median time when the wounds have reached at least 50% healing of the initial wound area.

In CGB, the rate of time to 50% healing of the probiotic treated wounds is 3.4 (95% CI: 0.9–12.8) times larger compared to the saline treated wounds (Fig 3). In ICGB the rate of time to 50% healing with probiotic treatment is 1.9 (95% CI: 0.4–9.8) times larger compared to saline treatment. Variable treatment remained in the best fitting model showing a higher rate of time to 50% healing in the probiotic group compared to the saline treated group (HR = 2.2, 95%CI: 0.8 to 6.1).

### Haematology

The SAA levels in the blood of most horses were within the reference range (0–30 mg/L) at day 0, day 3 and day 9, and therefore statistical analysis was not performed. Only three horses had a level above the reference in one or two of their samples. The WBC of these three horses was also above reference value. One horse (treated with probiotics) had a normal SAA level at the day of inclusion and at day 3, but the SAA level increased to a remarkable high value at day 9. Two of these horses (treated with saline) had an elevated level at time of inclusion, for one horse the increased SAA level declined to zero at day 9, but for the other horse it increased further with time.

The mean WBC at day 0 was 10.3 ± 2.8 G/l for the horses in the probiotic treatment, with 5 horses having values above the reference value of 5–10 G/l. For the horses in the saline treatment the mean WBC was 9.4 ± 3.4 G/l with 4 horses above reference level. The mean WBC decreased from day 0 to day 9 for both probiotic and saline treatment, to resp. 9.4 ± 3.3 G/l and 7.7 ± 2.4 G/l, as well as the number of horses above reference level, to resp. 3 horses and 1 horse. The mean percentage neutrophils of horses treated with probiotics and saline were within the reference range for day 0, day 3 and day 9.

### Bacteriology

**Fig 4:**
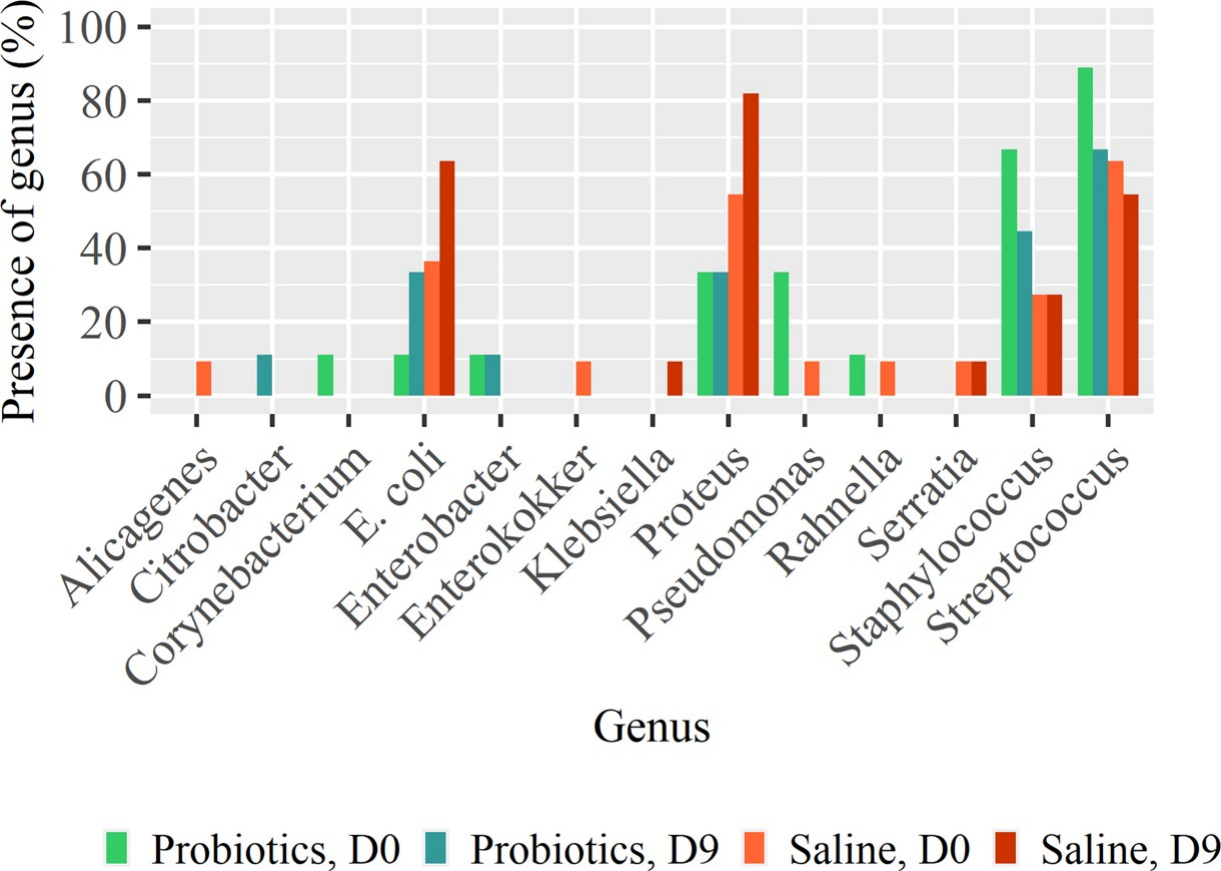
The percentage of probiotic- and saline treated wounds positive on culture of bacteria from various genus at day 0 and day 9.

Twenty-seven different bacterial species were cultured from the wound swabs obtained at day 0, day 3 and day 9 (S1 Table). At day 0, on average 2.7 (SD = 0.9) resp. 2.3 (SD = 0.8) different bacterial species were found in resp. the probiotic and the saline-treated wounds (range 1–4 spp) and the score for the quantification was 4.4 (SD = 1.9) resp 4.8 (SD = 2.0) (range 1–10). At day 9 the average number of bacterial species decreased to 2.0 (SD = 0.7) in the probiotic treated wounds, but remained 2.5 (SD = 1.1) in the saline treated wounds, whereas the score for quantification decreased in the probiotic treated wounds to 3.9 (SD = 2.1) but increased in the saline treated wounds to 5.2 (SD = 1.9). In the control group 3 of 11 horses showed a decreased bacterial load whereas in probiotic treated horses 6 of 9 horses did. The estimated OR was 4.9 (95% CI: 0.6 to 54.9, p = 0.17). The same results were observed for the decrease of the number of bacteria species. The bacteria species *Escherichia coli* and *Proteus mirabilis* were present initially in both treatment groups, but at day 9 in twice as many saline-treated wounds than probiotic-treated wounds. The bacteria species *Staphylococcae* and *Streptococcae* were present in more probiotic treated wounds at day 0, and the number reduced more than the number in the saline treated wounds. *Pseudomonas spp*. was cultured at day 0 from 3 probiotic-treated wounds and 1 saline-treated wound but not cultured anymore at day 9 (Fig 4).

### Qualitative scored outcomes

None of the horses showed overt signs of pain in relation to the wounds or lameness at the walk during the study. Slight skin irritation of the surrounding skin was noticed in a few cases, both probiotic and saline treated.

The average exudate level was similar in both treatment groups at inclusion. Thereafter, the exudate level decreased in the probiotic treated wounds, whereas it increased in the saline treated wounds to a peak at day 6, remaining higher for the rest of the study period. The amount of exudate was not significantly different between the treatments. The exudate of the probiotic treated wounds was less malodorous than that of the saline treated wounds throughout the study period. During the first 12 days, the difference was statistical significant (p_12_ = 0.05).

The surface of the wounds treated with probiotics was more regular from day 9 onwards compared to the controls, although the surface of the probiotic treated wounds had more clefts and were slightly deeper at the start of the study. EGT was seen more often at the start and during the first half of the study in the probiotic-treated wounds compared to the controls and these wounds were excised more often.

## Discussion

The present study showed that the mean relative wound area of the probiotic treated wounds decreased faster than that of the saline treated wounds. This difference was most obvious in the wounds with a complete granulation bed (CGB) at the start of the study where the mean wound area decreased to 28.4% (range: 6.3 to 49.3) with probiotic and to 51.9% (range: 29.3 to 81.7) with saline treatment at day 24. The estimated difference in CGB between the mean percentage wound area with probiotic treatment and saline treatment increased rapidly from the start of the study until day 12 up to 30 percentage points, and stabilized around a difference of 25 percentage points thereafter until the end of the observation period. Additionally, the rate of time to 50% healing in CGB was 3.4 (95% CI: 0.9–12.8) times larger with probiotic compared to saline treatment. Although the mentioned differences were not statistically significant, the clinical relevance of the results are evident but can be discussed. A difference in mean relative wound area between probiotic and saline treatment of more than 20 percentage points with a speed of healing of roughly 6 percentage points per week after day 12, when the curves of both treatments were more stabilized, means a decrease in treatment period of roughly 3 weeks. Also the time required to reach 50% healing visualizes the clinical relevance in CGB: 50% healing of the probiotic treated wounds is reached on average at day 12, whereas this was not completely reached for the saline treated wounds at day 24. This means that in the 24 days evaluation, the time for 50% reduction in wound area is reached in 50% of the time which certainly seems to be clinical relevant.

The differences in mean relative wound area of the probiotic treated wounds compared to the saline treated wounds was less obvious in the wounds that were not completely filled with granulation tissue at the start of the study (ICGB). These wounds were younger than the CGB wounds. Wounds healing by second intention in general increase in size straight after wounding. Small equine experimental wounds enlarge already up to 2 weeks [6]. This means that the younger wounds are expected to enlarge, whereas older wounds reduce in size from the beginning of treatment, and reduction is relatively fast during the contraction phase and slow during the final phase of epithelialization [6]. In the present study we have attempted to limit the variation in healing curves by the inclusion criteria and by making two wound groups: ICGB and CGB. These groups were determined by the phase of healing with tipping point the completeness of the granulation bed because a complete granulation bed means that contraction will principally occur. Half of the ICGB wounds enlarged in the beginning of the study (irrespective of treatment) which created more variation in the healing curves of ICGB compared to CGB. The fact that the effect of the probiotic dressing was most obvious in CGB wounds is likely to be the result of the more uniform healing curves within CGB: most wounds decreased in size from the start of the study, only some enlargement was seen at day 1 after debridement which was mainly reduced at day 3. Two control wounds from ICGB were excluded because of a necrotic skin flap and wound dehiscence of a partial suture, which caused an immediate enlargement of 165% resp. 300%. If these 2 wounds would remain in the data for analysis the estimated mean wound area in the saline treated group would be much larger consequently enlarging the difference with the mean area of the probiotic treated wounds. So this exclusion decreased the difference between the mean relative wound areas of probiotic and saline treatment.

The number of horses in this study was too small relative to the variation in wounds and healing curves to show significance. Despite this short-coming, the pattern of healing of the horses within the treatment groups appeared very similar. It is generally known that it is challenging to achieve enough power in clinical wound studies to prove statistical significance between treatments. The number of patients is usually limited because of expenses and the time duration to collect suitable patients. In the current study it took over 2 years to collect 30 horses with suitable wounds in 2 countries, which was partly due to the inclusion criteria. Control wounds should be treated properly because of ethical considerations and responsibility to patients, limiting the difference between a product and control treatment. In this study a Superabsorbent polyacrylate fibre dressings (Oasis SAF, Brightwake Ltd., United Kingdom) was used as base for both treatments because of its positive effect on healing [32], and its capacity to absorb and retain sufficient volume of the probiotic suspension. The non-woven material of the dressing gelates, when coming in contact with probiotic microorganisms, saline or exudate, proving a moist wound environment conducive for healing. It has been suggested, however, that the dressing exhibits an antimicrobial activity in vitro by absorbing and retaining bacteria between the fibers [32]. Such effect of the dressing in vivo may have made the additional effect of probiotics to reduce the number of wound bacteria in the probiotic group and the effect on healing harder to detect. It could even be disputed whether the dressing might have had some limiting effect on the working of the bacteria in the probiotic suspension by retaining them too strongly, although we have no indications for that.

Last but not least clinical wounds are very diverse which consequently affects the healing curves and rate of healing due to wound factors, patient factors and phase of healing. Wound factors such as location, depth, size, bacterial load [1] and microbial diversity of the wound influence the healing curve. Patient factors such as age and body condition can create variation. Additionally, healing varies dependent on the time duration of the wound and the phase of healing as mentioned before.

The present study showed that the topical use of probiotics was well-tolerated by all horses and no systemic inflammatory reaction could be observed: the horses did not show clinical symptoms, no increase of SAA levels and no elevation of WBC counts. The equine species reacts very sensitive to inflammatory insults by rapid induction of the inflammatory marker SAA, which is faster and more accurate than changes in values of WBC [26, 33]. The SAA level of 14 out of 15 horses treated with probiotics did not increase at day 3 and day 9 and remained within the reference values suggesting that the topical applied probiotic bacteria do not enter the body and do not trigger the immune system. The only horse treated with probiotics with an increased SAA level at day 9, had an upper respiratory tract infection with nasal discharge diagnosed the same day, which can explain the high SAA value. Two horses treated with saline had increased SAA levels at time of inclusion. For one horse this increased further with time which was most likely related to a developing wound infection that resulted in wound dehiscence of the partially sutured wound at day 7. Probiotic bacteria, and in particularly lactobacilli and bifidobacteria, are generally recognized as safe [14, 34–36], which is confirmed by this study.

The wound healing tendency of probiotics in the current study is supported by the reduction of the amount of exudate and malodour, as well as a lower score for the number of bacteria and number of bacteria species in the probiotic-treated compared to the saline-treated wounds.

For bacteriology, swabs were chosen because of their non-invasiveness, although culture may over-represent surface bacteria and underestimate isolates that resides deeper within the wound bed [37]. Twenty-seven bacterial species were identified from culture of the swabs obtained from the wounds at day 0, 3 and 9. The isolation of several bacterial strains per swab is in accordance with previous studies showing that equine wounds harbour a polymicrobial bacterial load [38]. The bacteria species *Escherichia coli* and *Proteus mirabilis* were present initially in both groups, but at day 9 in twice as many wounds treated with saline than wounds treated with probiotics. This is in accordance with Westgate *et al*. [2] who found high percentages of the bacterial genera *Proteus* and *Escherichia coli* isolated from chronic wounds of horses. The same authors also reported a significant biofilm forming potential of gram negative isolates of trauma wounds and that the genera *Proteus* isolated from wounds displayed stronger attachment, indicative of biofilm formation, than equivalent skin isolates. In vitro studies have demonstrated that probiotic bacteria are able to inhibit growth of clinical isolates by suppression of various virulence factors, such as biofilm forming potential [13, 25, 39]. We have found that *Pseudomonas spp*. disappeared from the probiotic treated wounds. This coincides with experimental studies on infected burns in rats where growth of *Pseudomonas aeruginosa* was inhibited by kefir products or *Lactobacillus plantarum* [22, 23]. The reduction of the score for quantification and the number of bacteria species in probiotic treated wounds coincides with other studies that found a reduction of the bacterial load by topical application of probiotics [22, 24]. The probiotic treated wounds of CGB may have profited most by the reduction of the bacterial load as this also decreases the chronic inflammatory response inherently present in the later phases of the equine healing process [8]. Chronic inflammation inhibits wound contraction and delays healing [1, 40].

In the present study total doses of 5x10^6^–10^9^ CFU/ml was used as this content interval could be guaranteed from the manufacturer (Biosa Aps, Denmark). For comparison, studies in humans and laboratory rodents investigating the effect of topical treatment with *Lactobacillus plantarum* on diabetic leg ulcers and burn wounds have used 10^5^−10^8^ CFU/ml [22, 24, 25]. *Lactobacillus acidophilus*, *Bifidobacterium animalis* subsp *lactis*, *Lactobacillus paracasei* subsp *paracasei* were used in this study as they are generally considered safe [14, 34–36], were available in standardized batches of guaranteed contents, and all have documented antimicrobial effect on clinical isolates when tested *in vitro* [15, 39]. The treatment regimen with application of the probiotic suspension every third day throughout the study period was selected empirically, partly to limit the expenses associated with daily bandage changes. In a clinical study of the effect of *Lactobacillus plantarum* on chronic infected leg ulcers in humans the application was once daily over a 10 day period [24]. Future studies are needed to investigate the effect of different treatment regimens and to determine optimal dosing and treatment intervals [35].

## Conclusions

The present study showed that the mean relative wound area of the probiotic treated wounds decreased faster than that of the saline treated wounds. This difference was most obvious in the wounds with a complete granulation bed at the start of the study, but the difference between the treatment groups was not statistically significant because of the large variation within the treatment groups. However, the clinical relevance of the effect of treatment in CGB wounds is evident, supported by the estimated differences and the time required to reach 50% healing. The probiotic treated wounds reached 50% healing at day 12, whereas this was not completely reached for the saline treated wounds at day 24. This means that within the 24 days evaluation, the probiotic treated wounds reach 50% reduction in wound area in half of the time of the saline treated wounds. The topical use of probiotics can be considered as safe as it did not cause a systemic inflammatory response.

## Supporting information

S1 Details Details of the probiotic bacterial strains used in this study. (PDF)

S1 Data. Data set used for preparing the manuscript (XLSX)

S1 Table Presence of bacterial species in the wounds. Number of wounds with presence of bacterial species at day 0, 3, and 9 per treatment group.
(DOCX)

## Abbreviations

CGB: complete granulation bed
EGT: exuberant granulation tissue
ICGB: incomplete granulation bed
SAA: serum amyloid A
WBC: white blood cell count
