# Supplementary material for: The evaluation of the effect of probiotics on the healing of equine distal limb wounds
Source: PLoS One. 2020 Jul 29;15(7):e0236761. doi: 10.1371/journal.pone.0236761 (PMC7390451; doi:10.1371/journal.pone.0236761)
Supplement: S1 Details — (PDF) [file pone.0236761.s001.pdf]

Product datasheet

Vita Biosa Probiotic Herbs

**Description**	Vita Biosa Probiotic Herbs contains an herbal extract of 19 organic herbs. It has been fermented with a combination of 7 probiotics and lactic acid bacteria cultures. Lactic and acetic acids are formed during fermentation. This gives a low pH value of about 3.5. The low pH prevents the development of harmful bacteria in the finished product.
**Appearance**	Liquid
**Colour**	Brown
**Smell**	Sour – sweet
**pH**	Approximately 3.5
**Ingredients**	Water, organic fermented molasses from sugarcane, organic fermented invert sugar = fructose + glucose, organic herbs and fermentation cultures.
**Herbs**	Herbs: The total content of herbs is approximately 0.4% of the total volume in the finished product. The percentages of herbs in the mixture are as follows:<br><br>Angelica archangelica (angelica) 4.5%<br>Anethum graveolens (dill) 4.5%<br>Anthriscus cerefolium (chervil) 4.5%<br>Foeniculum vulgare (fennel) 9%<br>Glycyrrhiza glabra (liquorice root) 9%<br>Juniperus communis (juniper) 4.5%<br>Matricaria recutita (chamomile) 4.5%<br>Mentha piperita (peppermint) 4.5%<br>Ocimum basilicum (basil) 4.5%<br>Origanum vulgare (oregano) 4.5%<br>Petroselium crispum (parsley) 4.5%<br>Pimpinella anisum (anise) 9%<br>Rosmarinus officinalis (rosemary) 4.5%<br>Salvia officinalis (sage) 4.5%<br>Sambucus nigra (elder) 4.5%<br>Thymus vulgaris (thyme) 4.5%<br>Trigonella foenum-graecum (fenugreek) 4.5%<br>Urtica dioica (nettle) 4.5%<br>Zingiber officinale (ginger root) 4.5%
**Lactic acid bacteria and probiotic fermentation cultures**	*Lactobacillus acidophilus* LA5®<br>*Bifidobacterium animalis* subsp. *lactis* BB12®<br>*Lactobacillus paracasei* subsp. *paracasei* L.casei 431®<br>*Streptococcus thermophilus*,<br>*Lactococcus lactis* subsp. *lactis*,<br>*Lactococcus lactis* subsp. *lactis biovar diacetylactis* and<br>*Leuconostoc pseudomesenteroides*<br>Other fermentation cultures may occur naturally from the raw materials.
**Sugar – sugar free**	During the fermentation the 7 cultures break down all the sugar and convert it into lactic and acetic acids. When all the sugar is used the fermentation is finish and the product is sugar free.
**GMO Declaration**	The product does not contain any Genetic Modified Organism (GMO - free).
**Organic Declaration**	The product is 100% organic. Certified by the Danish inspection system concerning organic food, Council Regulation (EC) no 834/2007.
**CFU/ml**	The amount of viable bacteria (CFU/ml) increases a lot during the production of Vita Biosa Probiotic, a development that enhances the quality of the product. The number decreases again during storage; hence the exact number will vary over time. At the date of production the CFU is approximately 100 mio/ml. At the end of the shelf life (one year) the CFU has decreased to approximately 20 mio/ml.
**Dose**	Vita Biosa Probiotic is a living product containing 20 million/ml viable bacteria at the end of shelf life. The recommended daily intake of Vita Biosa Probiotic is 20-50 ml. To disperse any sediment, shake gently before use. Vita Biosa Probiotic can be enjoyed at any time of the day – preferable with meals.

Biosa Danmark ApS, Sonnerupvej 41, DK- 3300 Frederiksværk
Phone: +45 4777 2270 Fax: +45 4772 4011
E-mail: info@biosa.dk Home page: www.biosa.dk

<table>
<tr><td>Storage</td><td>Before opening: Avoid frost. Keep out of direct sunlight.
After opening: Dark and cold. Max. 8°C.</td></tr>
<tr><td>Keeping qualities</td><td>Unopened - all packing: 12 months after production date.
After opening: Bottles - 2 month at max. 8°C.
Bag in box - 6 months after production by room temperature.
Best before date stated as dd.mm.yyyy is placed on the label of each bottle.</td></tr>
<tr><td>Nutritive value in 100 ml.</td><td>
<table>
<tr><td>Energy</td><td>50 kJ / 15 Kcal</td></tr>
<tr><td>Protein</td><td>0 – 1 g</td></tr>
<tr><td>Carbohydrate</td><td>1.5 g</td></tr>
<tr><td>Of which sugars</td><td>0 – 0.5 g</td></tr>
<tr><td>Fat</td><td>0 – 1 g</td></tr>
<tr><td>Of which saturated</td><td>n.a.</td></tr>
<tr><td>Fibre</td><td>0 – 1 g</td></tr>
<tr><td>Natrium</td><td>0 g</td></tr>
</table>
</td></tr>
<tr><td>Packaging</td><td>½ litre PET bottle.
1 litre PET bottle.
3 litre bag in box (EVOH film in cardboard box).</td></tr>
<tr><td>Control</td><td>Steins Laboratories Denmark controls each batch/lot.</td></tr>
<tr><td>Producer</td><td>Biosa Danmark ApS, Sonnerupvej 41, DK 3300 Frederiksværk
Production: Syrevej 51, DK - 3300 Frederiksværk
Organic control number: DK-ØKO-200</td></tr>
<tr><td>Date</td><td>February 2012
This datasheet replaces datasheets older than the above mentioned date.</td></tr>
</table>

CHR HANSEN

Improving food & health

Probio-Tec® BB-12® Blend-30 IF

Product Specification

Form: FD powder
Item no: 700330
Country of Origin: Denmark

**Performance**	**Specification**
Total cell count (cfu/g)<br>*DK-BIRA-QAm-065*	>=3.0E+10

**Physical properties**	**Specification**
Water activity (Aw)<br>*G-AM-Cul-012*	<=0.15
Visual control of powder<br>*Global-Prod-AM-Cul-040*	White to light beige fine powder

**Purity**	**Specification**
S. aureus (cfu/g)<br>*Ph Eur 2.6.13*	<10
Total Yeasts & Moulds (cfu/g)<br>*Ph. Eur 2.6.12 (modified)*	<=100
Bacillus cereus (cfu/g)<br>*DK-BIRA-QAm-037*	<100
Total aerobic bacterial count (cfu/g)<br>*Ph. Eur 2.6.13 (modified)*	<=2000
Enterobacteriaceae<br>*ISO 21528 (modified)*	Absent/10x10g
Ent. sakazakii<br>*ISO 21528 (modified)*	Absent/10x10g
Salmonella<br>*AOAC 996.08 VIDAS*	Absent/10x10g

Chr. Hansen A/S - Bøge Allé 10-12 - 2970 Hørsholm - Denmark - Phone: +45 45 74 74 74 / Fax: +45 45 74 88 89

CHR HANSEN

Probio-Tec® BB-12® Blend-30 IF

GMO statement

Material No: 700330

Version: 1 GMO-EN 03-09-2010

GMO statement concerning Probio-Tec® BB-12® Blend-30 IF

According to the legislation in the European Union genetic modification occurs if certain techniques have been used*. The same legislation also defines techniques, which will not result in genetic modification. In accordance with this legislation we can state that

Probio-Tec® BB-12® Blend-30 IF does not contain GMOs and does not contain GM labeled raw materials**.

GM labeling information concerning Probio-Tec® BB-12® Blend-30 IF

Legislation in the European Union states that a final food product must be labeled if it is a GMO itself, if it contains GMOs, or if it contains ingredients derived from GMOs**.

In accordance with European legislation on labeling of final food products we can inform that

The use of Probio-Tec® BB-12® Blend-30 IF does not trigger a GM labeling of the final food product.

Chr. Hansen's position on GMO can be found on:
www.chr-hansen.com/About us/Policies and positions/Quality and product safety.

* Directive 2001/18/EC of the European Parliament and of the Council of 12 March 2001 on the deliberate release into the environment of genetically modified organisms and repealing Council Directive 90/220/EEC.

** Regulation (EC) No 1829/2003 of the European Parliament and of the Council of 22 September 2003 on genetically modified food and feed.
Regulation (EC) No 1830/2003 of the European Parliament and of the Council of 22 September 2003 concerning the traceability and labeling of genetically modified organisms and the traceability of food and feed products produced from genetically modified organisms and amending Directive 2001/18/EC.

The information contained herein is to the best of our knowledge true and correct and presented in good faith. It may be subject to change without further notice. To the best of our knowledge this product does not infringe Intellectual Property Rights of any third party. This information is offered solely for your consideration and verification. Copyright© Chr. Hansen A/S. All rights reserved.

CHR HANSEN

Improving food & health

Probio-Tec® BB-12® Blend-30 IF

Certificate of Analysis

**Form:**	FD powder	**Batch no:**	3080492
**Item no:**	700330	**Date of Manufacture:**	05/2012
**Country of origin:**	Denmark	**Expiry date:**	05/2014

**Performance**	**Result**	**Specification**
Total cell count (cfu/g)<br>*DK-BIRA-QAm-065*	3.9E+10	>=3.0E+10
**Physical properties**	**Result**	**Specification**
Visual control of powder<br>*Global-Prod-AM-Cul-040*	White to light beige fine powder	White to light beige fine powder
Water activity (Aw)<br>*G-AM-Cul-012*	0.06	<=0.15
**Purity**	**Result**	**Specification**
Bacillus cereus (cfu/g)<br>*DK-BIRA-QAm-037*	<10	<100
Enterobacteriaceae<br>*ISO 21528 (modified)*	Absent/10x10g	Absent/10x10g
Ent. sakazakii<br>*ISO 21528 (modified)*	Absent/10x10g	Absent/10x10g
Salmonella<br>*AOAC 996.08 VIDAS*	Absent/10x10g	Absent/10x10g
S. aureus (cfu/g)<br>*Ph Eur 2.6.13*	<10	<10 *
Total aerobic bacterial count (cfu/g)<br>*Ph. Eur 2.6.13 (modified)*	<50	<=2000
Total Yeasts & Moulds (cfu/g)<br>*Ph. Eur 2.6.12 (modified)*	<5	<=100

*** Not detected in 0.1 g**

Søren Holm

Søren Holm

First Authorised 12/JUN/2012

Chr. Hansen A/S - Bøge Allé 10-12 - 2970 Hørsholm - Denmark - Phone: +45 45 74 74 74 / Fax: +45 45 74 88 89

CHR HANSEN

Improving food & health

La-5 Powder

Product Specification

Form: FD powder
Item no: 609854
Country of Origin: Denmark

Performance	Specification
Total cell count (cfu/g)<br>*DK-BIRA-QAm-017*	>=3.0E+10

Physical properties	Specification
Water activity (Aw)<br>*G-AM-Cul-012*	<0.15
Visual control of powder<br>*Global-Prod-AM-Cul-040*	(almost) White crystal. powder

Purity	Specification
Total Yeasts & Moulds (cfu/g)<br>*Ph. Eur 2.6.12 (modified)*	<=200
Total viable aerobic count (cfu/g)<br>*Ph. Eur 2.6.12*	<=2000
E. coli<br>*Ph Eur 2.6.13*	Absent/g

Chr. Hansen A/S - Bøge Allé 10-12 - 2970 Hørsholm - Denmark - Phone: +45 45 74 74 74 / Fax: +45 45 74 88 89

CHR HANSEN

Probio-Tec® FD La-5 Powder

GMO statement

Material No: 609854

Version: 4 GM0-EN 01-04-2010

GMO statement concerning Probio-Tec® FD La-5 Powder

According to the legislation in the European Union genetic modification occurs if certain techniques have been used*. The same legislation also defines techniques, which will not result in genetic modification. In accordance with this legislation we can state that

Probio-Tec® FD La-5 Powder does not contain GMOs and does not contain GM labeled raw materials**.

GM labeling information concerning Probio-Tec® FD La-5 Powder

Legislation in the European Union states that a final food product must be labeled if it is a GMO itself, if it contains GMOs, or if it contains ingredients derived from GMOs**.

In accordance with European legislation on labeling of final food products we can inform that

The use of Probio-Tec® FD La-5 Powder does not trigger a GM labeling of the final food product.

Chr. Hansen's position on GMO can be found on:
www.chr-hansen.com/About us/Policies and positions/Quality and product safety.

* Directive 2001/18/EC of the European Parliament and of the Council of 12 March 2001 on the deliberate release into the environment of genetically modified organisms and repealing Council Directive 90/220/EEC.

** Regulation (EC) No 1829/2003 of the European Parliament and of the Council of 22 September 2003 on genetically modified food and feed.
Regulation (EC) No 1830/2003 of the European Parliament and of the Council of 22 September 2003 concerning the traceability and labeling of genetically modified organisms and the traceability of food and feed products produced from genetically modified organisms and amending Directive 2001/18/EC.

The information contained herein is to the best of our knowledge true and correct and presented in good faith. It may be subject to change without further notice. To the best of our knowledge this product does not infringe Intellectual Property Rights of any third party. This information is offered solely for your consideration and verification. Copyright© Chr. Hansen A/S. All rights reserved.

CHR HANSEN

Improving food & health

La-5 Powder

Certificate of Analysis

**Form:**	FD powder	**Batch no:**	3079138
**Item no:**	609854	**Date of Manufacture:**	05/2012
**Country of origin:**	Denmark	**Expiry date:**	05/2014

**Performance**	**Result**	**Specification**
Total cell count (cfu/g)<br>*DK-BIRA-QAm-017*	3.8E+10	>=3.0E+10
**Physical properties**	**Result**	**Specification**
Visual control of powder<br>*Global-Prod-AM-Cul-040*	White to light red fine powder	White to light red fine powder
Water activity (Aw)<br>*G-AM-Cul-012*	0.02	<0.15
**Purity**	**Result**	**Specification**
E. coli<br>*Ph Eur 2.6.13*	Absent/g	Absent/g
Total viable aerobic count (cfu/g)<br>*Ph. Eur 2.6.12*	<5	<=2000
Total Yeasts & Moulds (cfu/g)<br>*Ph. Eur 2.6.12 (modified)*	<5	<=200

Søren Holm

Søren Holm

First Authorised 06/JUN/2012

www.chr-hansen.com

Version: 28/APR/2009

Chr. Hansen A/S - Bøge Allé 10-12 - 2970 Hørsholm - Denmark - Phone: +45 45 74 74 74 / Fax: +45 45 74 88 89

CHR HANSEN

Improving food & health

Probio-Tec® C-Powder-30

Product Specification

Form: FD powder
Item no: 687018
Country of Origin: Denmark

Performance	Specification
Total cell count (cfu/g) *DK-BIRA-QAm-017*	>=3.0E+10

Physical properties	Specification
Water activity (Aw) *G-AM-Cul-012*	<0.15
Visual control of powder *Global-Prod-AM-Cul-040*	White, light beige to light reddish fine powder

Purity	Specification
S. aureus (cfu/g) *Ph Eur 2.6.13*	<10
Total Yeasts & Moulds (cfu/g) *Ph. Eur 2.6.12 (modified)*	<=100
Bacillus cereus (cfu/g) *DK-BIRA-QAm-037*	<100
Total aerobic bacterial count (cfu/g) *Ph. Eur 2.6.13 (modified)*	<=2000
Enterobacteriaceae *ISO 21528 (modified)*	Absent/10x10g
Ent. sakazakii *ISO 21528 (modified)*	Absent/10x10g
Salmonella *AOAC 996.08 VIDAS*	Absent/10x10g

Chr. Hansen A/S - Bøge Allé 10-12 - 2970 Hørsholm - Denmark - Phone: +45 45 74 74 74 / Fax: +45 45 74 88 89

CHR HANSEN

Probio-Tec® C-Powder-30

GMO statement

Material No: 687018

Version: 3 GM0-EN 01-04-2010

GMO statement concerning Probio-Tec® C-Powder-30

According to the legislation in the European Union genetic modification occurs if certain techniques have been used*. The same legislation also defines techniques, which will not result in genetic modification. In accordance with this legislation we can state that

Probio-Tec® C-Powder-30 does not contain GMOs and does not contain GM labeled raw materials**.

GM labeling information concerning Probio-Tec® C-Powder-30

Legislation in the European Union states that a final food product must be labeled if it is a GMO itself, if it contains GMOs, or if it contains ingredients derived from GMOs**.

In accordance with European legislation on labeling of final food products we can inform that

The use of Probio-Tec® C-Powder-30 does not trigger a GM labeling of the final food product.

Chr. Hansen's position on GMO can be found on:
www.chr-hansen.com/About us/Policies and positions/Quality and product safety.

* Directive 2001/18/EC of the European Parliament and of the Council of 12 March 2001 on the deliberate release into the environment of genetically modified organisms and repealing Council Directive 90/220/EEC.

** Regulation (EC) No 1829/2003 of the European Parliament and of the Council of 22 September 2003 on genetically modified food and feed.
Regulation (EC) No 1830/2003 of the European Parliament and of the Council of 22 September 2003 concerning the traceability and labeling of genetically modified organisms and the traceability of food and feed products produced from genetically modified organisms and amending Directive 2001/18/EC.

The information contained herein is to the best of our knowledge true and correct and presented in good faith. It may be subject to change without further notice. To the best of our knowledge this product does not infringe Intellectual Property Rights of any third party. This information is offered solely for your consideration and verification. Copyright© Chr. Hansen A/S. All rights reserved.

CHR HANSEN

Improving food & health

Probio-Tec® C-Powder-30

Certificate of Analysis

**Form:**	FD powder	**Batch no:**	3072612
**Item no:**	687018	**Date of Manufacture:**	03/2012
**Country of origin:**	Denmark	**Expiry date:**	03/2014

**Performance**	**Result**	**Specification**
Total cell count (cfu/g)<br>*DK-BIRA-QAm-017*	5.2E+10	>=3.0E+10
**Physical properties**	**Result**	**Specification**
Visual control of powder<br>*Global-Prod-AM-Cul-040*	White, light beige to light reddish fine powder	White, light beige to light reddish fine powder
Water activity (Aw)<br>*G-AM-Cul-012*	0.09	<0.15
**Purity**	**Result**	**Specification**
Bacillus cereus (cfu/g)<br>*DK-BIRA-QAm-037*	<10	<100
Enterobacteriaceae<br>*ISO 21528 (modified)*	Absent/10x10g	Absent/10x10g
Ent. sakazakii<br>*ISO 21528 (modified)*	Absent/10x10g	Absent/10x10g
Salmonella<br>*AOAC 996.08 VIDAS*	Absent/10x10g	Absent/10x10g
S. aureus (cfu/g)<br>*Ph Eur 2.6.13*	<10	<10 *
Total aerobic bacterial count (cfu/g)<br>*Ph. Eur 2.6.13 (modified)*	<50	<=2000
Total Yeasts & Moulds (cfu/g)<br>*Ph. Eur 2.6.12 (modified)*	<5	<=100

*** Not detected in 0.1 g**

Søren Holm

Søren Holm

First Authorised 30/APR/2012

www.chr-hansen.com

Version: 24/APR/2012

Chr. Hansen A/S - Bøge Allé 10-12 - 2970 Hørsholm - Denmark - Phone: +45 45 74 74 74 / Fax: +45 45 74 88 89

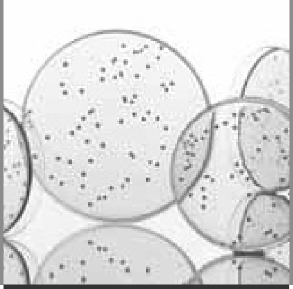

CHR HANSEN

Improving food & health

Probio-Tec®

Bifidobacterium BB-12®

Probio-Tec®
Bifidobacterium BB-12®
Chr. Hansen offers multiple application forms of one of our most clinically documented Probio-Tec® strains, Bifidobacterium BB-12®. BB-12® has been used since 1984 as an ingredient in food and dietary supplements worldwide. For over 20 years, BB-12® has been clinically studied for its health benefits and has demonstrated clinical efficacy in several areas, including Immune Health, Gastrointestinal Health, and relief of occasional diarrhea.

Benefits
Chr. Hansen's BB-12® multiple dosage forms offer the freedom to create a wide range of clinically documented probiotic products.

Benefits to the customer are:

- **Documented Effect:**
  BB-12® has extensively documented efficacy and safety
- **Recognized Strain:**
  BB-12® is one of the most recognized and documented probiotic strains available today in the global probiotic market
- **Multiple Applications and Dosage Forms:**
  BB-12® products are available in different dosage forms as well as customized solutions

Documented Effect
Probio-Tec® Bifidobacterium BB-12® has been used alone and in combination with other Chr. Hansen strains in over 200 studies to date, including human, in-vitro, and animal studies.

Specific BB-12® studies from Chr. Hansen's extensive study database include:

- Isolauri et al. (2000) demonstrates that BB-12® alleviates symptoms of atopic eczema
- Schiffrin et al. (1997) demonstrates that BB-12® has an immune modulating effect
- Sheu et al. (2002) demonstrates that BB-12® + LA-5® improves eradication of H. pylori and restores depletion of Bifidobacterium after triple therapy/antibiotic

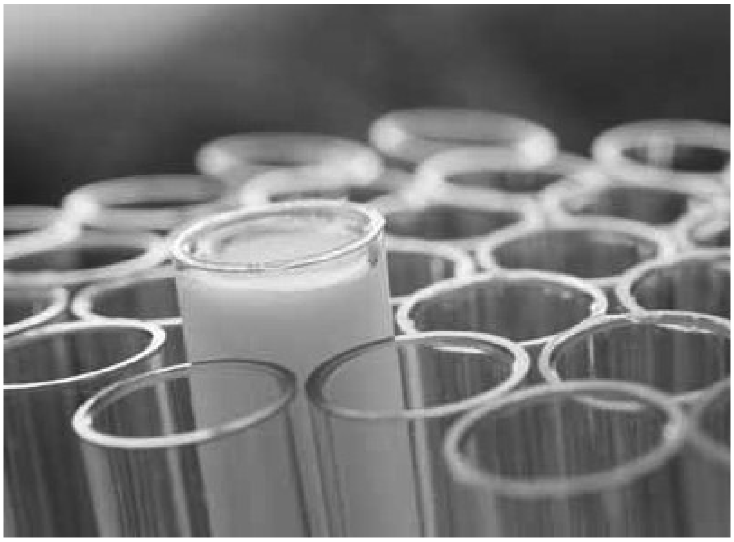

Probio-Tec®... when science matters

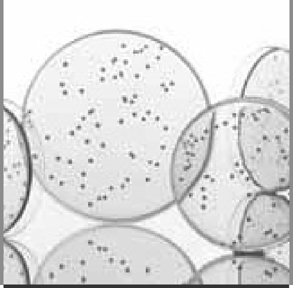

CHR HANSEN

Improving food & health

Product Positioning
Probio-Tec® Bifidobacterium BB-12® is well documented with many health benefits, with a focus on Gastrointestinal Health and more specifically, restoration of beneficial microbiota populations along the intestinal tract.

Depending on the final product design, dosage and dosage form, the product can be positioned according to market needs and targeted audience.

Some examples of available products are:

- BB-12® Bulk Powder Blends
- BB-12® Bulk Finished Capsules
- BB-12® Chewable Tablets
- BB-12® Stick Packs
- BB-12® Powder for Infant Formula

Customized BB-12® formulas are also available. Please contact your local Chr. Hansen Sales Manager for more information.

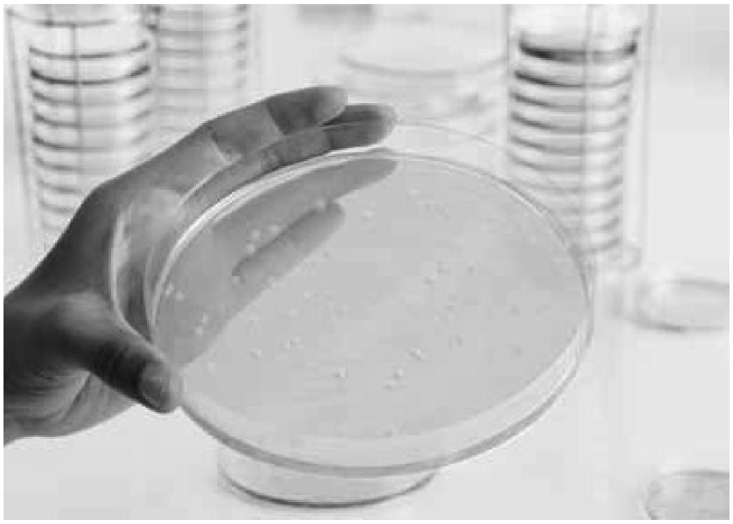

Probio-Tec®… when science matters
Chr. Hansen which was founded in 1874 by the Danish pharmacist Christian D.A. Hansen is a trusted supplier of clinically documented probiotic cultures for the dietary supplement, infant formula and pharmaceutical industries. Science and innovation are the cornerstone elements which allow Chr. Hansen to stand out as a trusted quality supplier of efficacious and safe probiotics.

Chr. Hansen Probiotics
Being the leading global manufacturer of clinically documented probiotics, Chr. Hansen's vast knowledge and experience within the probiotics industry ensure first quality, scientifically-based probiotic products. Our probiotics specialists provide the knowledge, inspiration, support, and customized solutions needed to create successful probiotic product portfolios.

Main References
Isolauri et al. Probiotics in the management of atopic eczema. Clinical and Experimental Allergy 2000;30:1604-1610

Schiffrin et al. Immune modulation of blood leucocytes in humans by lactic acid bacteria: criteria for strain selection. Am J Clin Nutr 1997;66:515S-520S

Sheu et al. Impact of supplement with Lactobacillus- and Bifidobacterium-containing yoghurt on triple therapy for Helicobacter pylori eradication. Aliment. Pharmacol. Ther. 2002;16:1669-1675

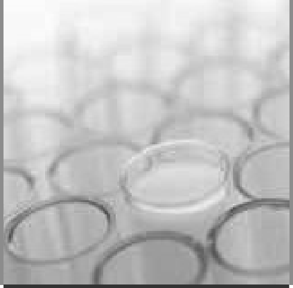

CHR HANSEN

Improving food & health

Probio-Tec®

Lactobacillus acidophilus LA-5®

Probio-Tec®
Lactobacillus acidophilus LA-5®
Chr. Hansen offers multiple application forms of one of our most clinically documented Probio-Tec® strains, Lactobacillus acidophilus LA-5®. LA-5® originates from Chr. Hansen's collection of dairy cultures and has been used since 1979 as an ingredient in food and dietary supplements worldwide with no reported adverse side effects. LA-5® has been clinically studied for its health benefits and has demonstrated clinical efficacy in several areas, including re-establishing gastrointestinal microbiota balance and relief of antibiotic-associated diarrhea.

Benefits
Chr. Hansen's LA-5® multiple dosage forms offer the freedom to create a wide range of clinically documented probiotic products.

Benefits to the customer are:

- **Documented Effect:**
  LA-5® has extensively documented efficacy and safety
- **Recognized Strain:**
  LA-5® is one of the most recognized and documented probiotic strains available today in the global probiotic market
- **Multiple Applications and Dosage Forms:**
  LA-5® products are available in different dosage forms as well as customized solutions

Documented Effect
Probio-Tec® Lactobacillus acidophilus LA-5® has been used alone and in combination with other Chr. Hansen strains in over 90 studies to date, including human, in-vitro, and animal studies.

Specific LA-5® studies from Chr. Hansen's extensive study database include:

- Nord et al. (1997) demonstrates that LA-5® + BB-12® enhances the re-colonization of the intestine after antibiotic treatment
- Sheu et al. (2002) demonstrates that LA-5® + BB-12® improves eradication of H. pylori and restores depletion of Bifidobacterium after triple therapy/antibiotic
- Shioya et al. (2000) demonstrates that LA-5® + BB-12® relieves constipation and improves intestinal conditions

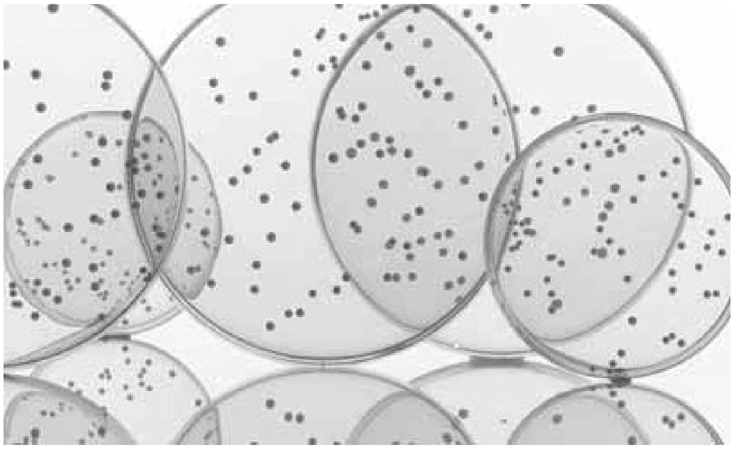

Probio-Tec®... when science matters

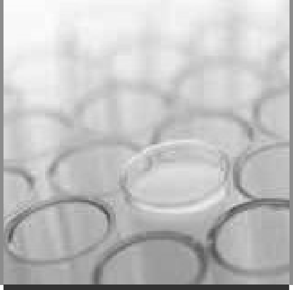

CHR HANSEN

Improving food & health

Product Positioning
Probio-Tec® Lactobacillus acidophilus LA-5® is well documented with many health benefits, with a focus on restoration of beneficial microbiota populations along the gastrointestinal tract which is often needed following antibiotic therapy or during periods of extended stress.

Depending on the final product design, dosage and dosage form, the product can be positioned according to market needs and targeted audience.

Some examples of available products are:

- LA-5® Bulk Powder Blends
- LA-5® Bulk Finished Capsules
- LA-5® Stick Packs
- LA-5® Chewable Tablets

Customized LA-5® formulas are also available. Please contact your local Chr. Hansen Sales Manager for more information.

Probio-Tec®… when science matters
Chr. Hansen which was founded in 1874 by the Danish pharmacist Christian D.A. Hansen is a trusted supplier of clinically documented probiotic cultures for the dietary supplement, infant formula and pharmaceutical industries. Science and innovation are the cornerstone elements which allow Chr. Hansen to stand out as a trusted quality supplier of efficacious and safe probiotics.

Chr. Hansen Probiotics
Being the leading global manufacturer of clinically documented probiotics, Chr. Hansen's vast knowledge and experience within the probiotics industry ensure first quality, scientifically-based probiotic products. Our probiotics specialists provide the knowledge, inspiration, support, and customized solutions needed to create successful probiotic product portfolios.

Main References

Nord et al. Oral supplementation with lactic acid bacteria during intake of clindamycin. Clinical Microbiology and Infection 1997;3(1):124-132

Sheu et al. Impact of supplement with Lactobacillus- and Bifidobacterium-containing yoghurt on triple therapy for Helicobacter pylori eradication. Aliment. Pharmacol. Ther. 2002;16:1669-1675

Shioya et al. Effect of fermented milk containing Bifidobacterium lactis FK 120 on the fecal flora and fecal properties in healthy female volunteers. Food Health and Nutrition Research, Journal of Nutritional Food 2000;3:7-18

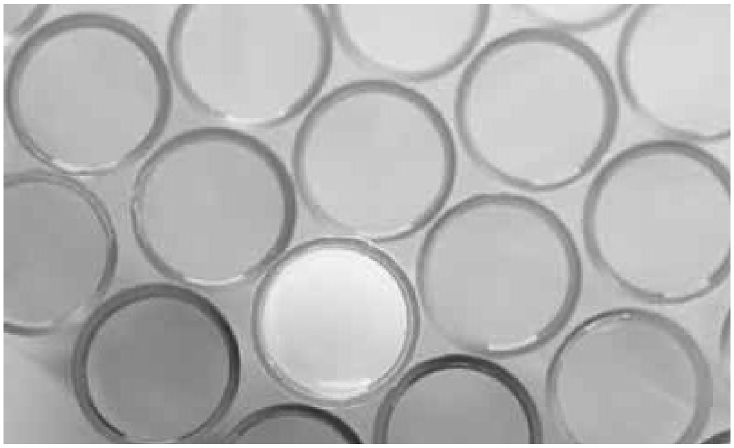

Probio-Tec®... when science matters

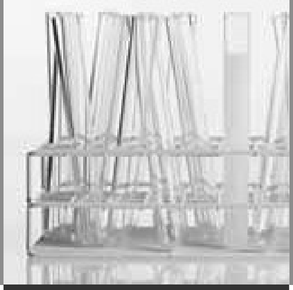

CHR HANSEN

Improving food & health

Probio-Tec®

Lactobacillus casei 431®

Probio-Tec®
Lactobacillus casei 431®
Chr. Hansen offers multiple application forms of one of our most clinically documented Probio-Tec® strains, Lactobacillus casei 431®. L. casei 431® was originally isolated from the stool of an infant and has been used since 1995 as an ingredient in food and dietary supplements worldwide with no reported adverse side effects. L. casei 431® has been clinically studied for its health benefits and has demonstrated clinical efficacy in boosting immunity by strengthening the body's natural defenses.

Benefits
Chr. Hansen's L. casei 431® multiple dosage forms offer the freedom to create a wide range of clinically documented probiotic products.

Benefits to the customer are:

- **Documented Effect:**
  L. casei 431® has extensively documented efficacy and safety
- **Multiple Applications and Dosage Forms:**
  L. casei 431® products are available in different dosage forms as well as customized solutions

Documented Effect
Probio-Tec® L. casei 431® has been used alone and in combination with other Chr. Hansen strains in over 50 studies to date, including human, in-vitro, and animal studies. Subjects have ranged from 5 months in age and upwards, with no reported adverse side effects.

Specific L. casei 431® studies from Chr. Hansen's extensive study database include:

- de Vrese et al. (2005) demonstrates that L. casei 431® enhance the immune response after vaccination
- Gaon et al. (2003) demonstrates that children treated with L. casei 431® had faster recovery from diarrheal episodes, illness and vomiting and L. casei 431® reduced the number of daily stools, diarrheal duration and vomiting
- Gonzalez et al. (1990) demonstrates that diarrhea was reduced and weight gain was improved in children who were supplemented with L. casei 431® and L. acidophilus

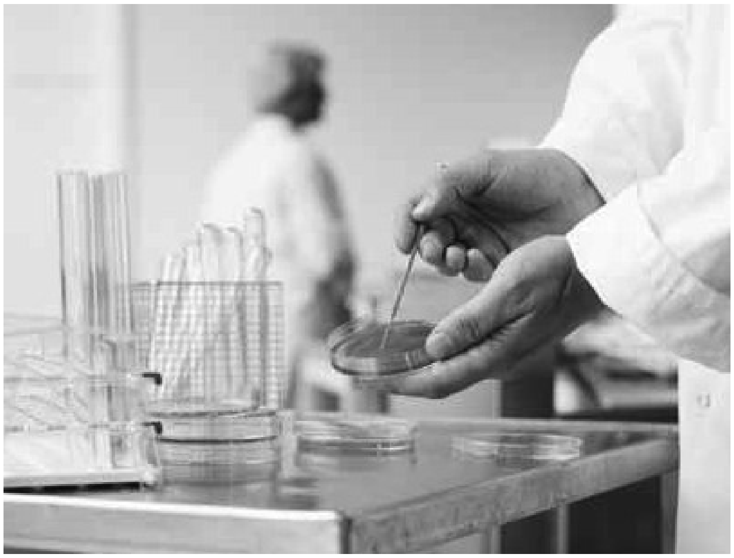

Probio-Tec®... when science matters

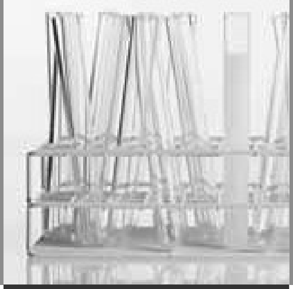

CHR HANSEN

Improving food & health

Product Positioning

Probio-Tec® L. casei 431® is well documented with many health benefits, with a focus on restoration of beneficial microbiota populations along the gastrointestinal tract which is often needed following antibiotic therapy or during periods of extended stress.

Depending on the final product design, dosage and dosage form, the product can be positioned according to market needs and targeted audience.

Some examples of available products are:

- L. casei 431® Bulk Powder Blends
- L. casei 431® Bulk Finished Capsules
- L. casei 431® Stick Packs

Customized L. casei 431® formulae are also available. Please contact your local Chr. Hansen Sales Manager for more information.

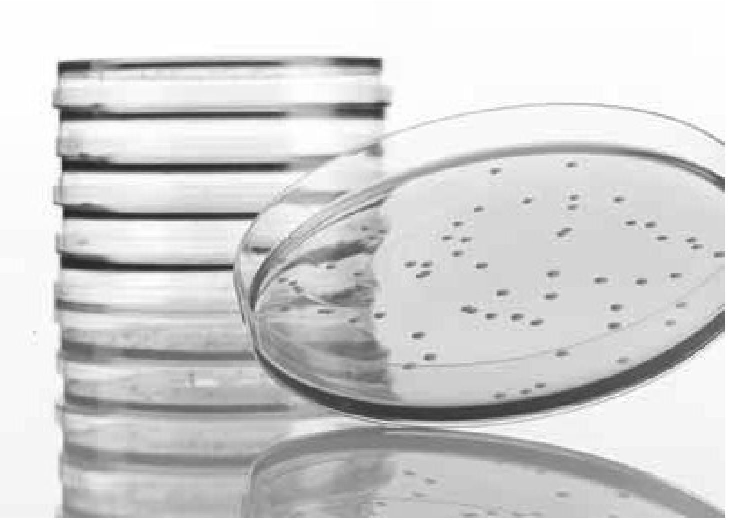

Probio-Tec®... when science matters

Chr. Hansen which was founded in 1874 by the Danish pharmacist Christian D.A. Hansen is a trusted supplier of clinically documented probiotic cultures for the dietary supplement, infant formula and pharmaceutical industries. Science and innovation are the cornerstone elements which allow Chr. Hansen to stand out as a trusted quality supplier of efficacious and safe probiotics.

Chr. Hansen Probiotics

Being the leading global manufacturer of clinically documented probiotics, Chr. Hansen's vast knowledge and experience within the probiotics industry ensure first quality, scientifically-based probiotic products. Our probiotics specialists provide the knowledge, inspiration, support, and customized solutions needed to create successful probiotic product portfolios.

Main References

de Vrese et al. Probiotic bacteria stimulate virus-specific neutralizing antibodies following a booster polio vaccination. Eur J Nutr 2005;44:406-413

Gaon et al. Effect of Lactobacillus strains and Saccharomyces boulardii on persistent diarrhea in children. Medicina 2003;63:293-298

Gonzalez et al. Prevention of infantile diarrhea by fermented milk. Microbiologie-Aliments-Nutrition 1990;8:349-354

CHR HANSEN

Study summaries BB-12®

This binder provides you with summaries of selected publications on *Bifidobacterium* BB-12® - one of the best documented probiotic strains.

The publications are clinical studies performed in humans documenting the effects in various conditions.

September 2011

Chr. Hansen A/S

Human Health & Nutrition

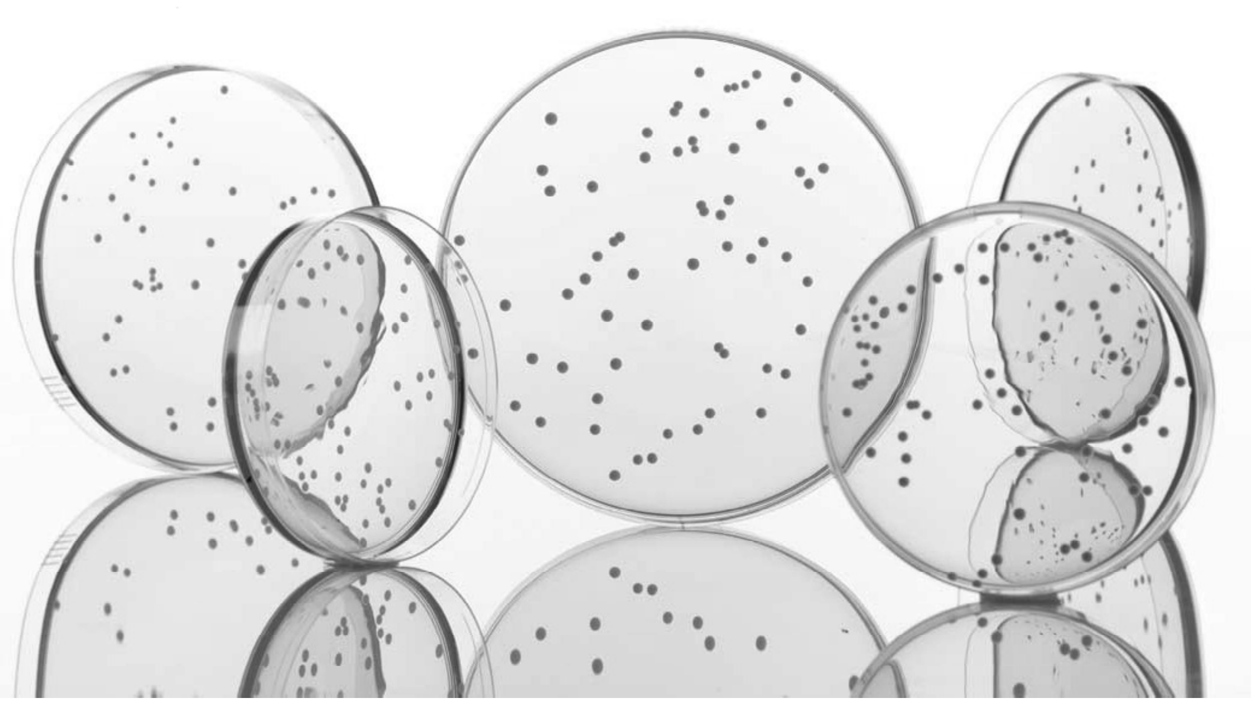

Chr. Hansen A/S -10-12 Bøge Allé – DK-2970 Hørsholm, Denmark - Phone: +45 45 74 74 74 - Fax: +45 45 74 88 88 - www.chr-hansen.com

The information contained herein is to the best of our knowledge true and correct and presented in good faith. It may be subject to change without further notice. To the best of our knowledge this product does not infringe Intellectual Property Rights of any third party. This information is offered solely for your consideration and verification. Copyright© Chr. Hansen A/S. All rights reserved.

CHR HANSEN

Research field: Other
Research subfield: Metabolic status
Study type: Human study
Probiotic strain: B. animalis subsp. lactis BB-12 and L. rhamnosus LGG
Dosage CFU/day: -
Product formulation: -
Reference number: 0756

Aaltonen, et al. Impact of maternal diet during pregnancy and breastfeeding on infant metabolic programming: a prospective randomized controlled study. Eur.J.Clin.Nutr. 2011;65:10

Abstract: Objectives:To evaluate the impact of maternal diet and intensive dietary counselling during pregnancy and breastfeeding on the infant's metabolic status.Subjects/Methods:At the first trimester of pregnancy, 256 women were randomized into a control/placebo group and two dietary counselling groups (diet/probiotics and diet/placebo). The counselling, with double-blind randomization to probiotics (Lactobacillus rhamnosus GG and Bifidobacterium lactis BB-12) or placebo, targeted excessive saturated fat and low fibre consumption. Maternal diet was evaluated repeatedly during pregnancy and postpartum by means of 3 days' food diaries. Metabolic markers, serum 32-33 split and intact proinsulin, leptin/adiponectin ratio, skinfold thickness and waist circumference were measured of 194 healthy infants at the age of 6 months, and the high levels were taken to mirror adverse metabolic status.Results:The proportion of infants with a high 32-33 split proinsulin was significantly lower in dietary counselling with probiotics (n=6/62, 9.7%) or placebo (n=7/69, 10.1%) compared with the control/placebo group (n=17/63, 27.0%). The high split proinsulin was associated with larger skinfold thickness, waist circumference and higher leptin/adiponectin ratio in the infants (P<0.05). With respect to maternal diet during pregnancy, the highest and lowest tertiles of fat intake increased the infant's risk of high split proinsulin, whereas those of butter associated correspondingly with the infant's waist circumference. Further, breastfed infants showed a reduced risk of high split proinsulin and leptin/adiponectin ratio compared with formula-fed infants.Conclusions:Modification of maternal diet during pregnancy and breastfeeding may benefit infant metabolic health. High split proinsulin reflects adverse metabolic status in infancy, which can be improved by early dietary counselling.

Chr. Hansen A/S -10-12 Bøge Allé – DK-2970 Hørsholm, Denmark - Phone: +45 45 74 74 74 - Fax: +45 45 74 88 88 - www.chr-hansen.com

The information contained herein is to the best of our knowledge true and correct and presented in good faith. It may be subject to change without further notice. To the best of our knowledge this product does not infringe Intellectual Property Rights of any third party. This information is offered solely for your consideration and verification. Copyright© Chr. Hansen A/S. All rights reserved.

Research field: Gastrointestinal Health
Research subfield: Diarrhea
Study type: Human study
Probiotic strain: BB-12 and LA-5
Dosage CFU/day: 4 billion
Product formulation: Capsules
Reference number: 1326

Bhalla. Randomized placebo-controlled, double blind, multicentric trial on Efficacy and Safety of Providac techsules (Lactobacillus acidophilus LA-5®, Bifidobacterium BB-12®) for prevention of Antibiotic-Associated Diarrhea in Indian patients. ACCP 2011

Abstract: Poster presented at the American College of Clinical Pharmacology (ACCP), 40th Annual Meeting October 2011

Chr. Hansen A/S -10-12 Bøge Allé – DK-2970 Hørsholm, Denmark - Phone: +45 45 74 74 74 - Fax: +45 45 74 88 88 - www.chr-hansen.com

The information contained herein is to the best of our knowledge true and correct and presented in good faith. It may be subject to change without further notice. To the best of our knowledge this product does not infringe Intellectual Property Rights of any third party. This information is offered solely for your consideration and verification. Copyright© Chr. Hansen A/S. All rights reserved.

CHR HANSEN

Research field: Immune Health
Research subfield: Immune stimulation
Study type: Human study
Probiotic strain: B. animalis subsp. lactis BB-12 and L. rhamnosus LGG
Dosage CFU/day: -
Product formulation: -
Reference number: 1319

Hoppu, et al. Probiotics and dietary counselling targeting maternal dietary fat intake modifies breast milk fatty acids and cytokines. Eur.J.Nutr. 2011

Abstract: PURPOSE: Breast milk fatty acids possess immunomodulatory properties, and new intervention strategies beyond supplementation of maternal diet with single oils are called for. The objective of the present study was to evaluate the effect of dietary intervention during pregnancy and breastfeeding on breast milk fatty acid and cytokine composition. METHODS: Pregnant women were randomised into three study groups: dietary intervention with probiotics (diet/probiotic) or with placebo (diet/placebo) and a control group (control/placebo). Dietary intervention included dietary counselling and provision of rapeseed oil-based food products. The probiotics used were Lactobacillus rhamnosus GG and Bifidobacterium lactis Bb12 in combination. Dietary intake was evaluated by food records at every trimester of pregnancy and 1 month postpartum. Breast milk samples were collected after birth (colostrum) and 1 month after delivery for fatty acid and cytokine analysis (n = 125). RESULTS: Dietary intervention improved the quality of fat in the diet. In breast milk, the proportion of α-linolenic acid and total n-3 fatty acids was higher in both dietary intervention groups compared with control group ($p < 0.05$). In the diet/probiotic group, the γ-linolenic acid content was higher compared with the diet/placebo group ($p < 0.05$). The concentrations of TNF-α, IL-10, IL-4 and IL-2 were higher in both dietary intervention groups compared with controls, and furthermore, long-chain n-3 fatty acids were associated with several cytokines in colostrum samples. CONCLUSION: The present intervention demonstrated the possibility of modifying breast milk immunomodulatory factors by dietary means.

The information contained herein is to the best of our knowledge true and correct and presented in good faith. It may be subject to change without further notice. To the best of our knowledge this product does not infringe Intellectual Property Rights of any third party. This information is offered solely for your consideration and verification. Copyright© Chr. Hansen A/S. All rights reserved.

CHR HANSEN

Research field: Immune Health
Research subfield: Immune stimulation
Study type: Human study
Probiotic strain: BB-12 and L. casei 431
Dosage CFU/day: 1 billion
Product formulation: Fermented milk (acidified milk) or capsules
Reference number: 1325

Rizzardini, et al. Evaluation of the immune benefits of two probiotic strains Bifidobacterium animalis ssp. lactis, BB-12(R) and Lactobacillus paracasei ssp. paracasei, L. casei 431(R) in an influenza vaccination model: a randomised, double-blind, placebo-controlled study Br.J.Nutr. 2011:1-9

Abstract: The present study investigated the ability of *Bifidobacterium animalis* ssp. *lactis* (BB-12®) and *Lactobacillus paracasei* ssp. *paracasei* (L. casei 431®) to modulate the immune system using a vaccination model in healthy subjects. A randomised, double-blind, placebo-controlled, parallel-group study was conducted in 211 subjects (56 % females, mean age 33·2 (sd 13·1) years). Subjects consumed a minimum of 10^9 colony-forming units of BB-12® (capsule) or L. casei 431® (dairy drink) or a matching placebo once daily for 6 weeks. After 2 weeks, a seasonal influenza vaccination was given. Plasma and saliva samples were collected at baseline and after 6 weeks for the analysis of antibodies, cytokines and innate immune parameters. Changes from baseline in vaccine-specific plasma IgG, IgG1 and IgG3 were significantly greater in both probiotic groups *v.* the corresponding placebo group (L. casei 431®, *P* = 0·01 for IgG; *P* < 0·001 for remaining comparisons). The number of subjects obtaining a substantial increase in specific IgG (defined as ≥ 2-fold above baseline) was significantly greater in both probiotic groups *v.* placebo (BB-12®, *P* < 0·001 for IgG, IgG1 and IgG3; L. casei 431®, *P* < 0·001 for IgG1 and IgG3). Significantly greater mean fold increases for vaccine-specific secretory IgA in saliva were observed in both probiotic groups *v.* placebo (BB-12®, *P* = 0·017; L. casei 431®, *P* = 0·035). Similar results were observed for total antibody concentrations. No differences were found for plasma cytokines or innate immune parameters. Data herein show that supplementation with BB-12® or L. casei 431® may be an effective means to improve immune function by augmenting systemic and mucosal immune responses to challenge.

The information contained herein is to the best of our knowledge true and correct and presented in good faith. It may be subject to change without further notice. To the best of our knowledge this product does not infringe Intellectual Property Rights of any third party. This information is offered solely for your consideration and verification. Copyright© Chr. Hansen A/S. All rights reserved.

CHR HANSEN

Research field: Gastrointestinal Health
Research subfield: Microbiota
Study type: Human study
Probiotic strain: B. animalis subsp. lactis BB-12 and L. acidophilus LA-5
Dosage CFU/day: -
Product formulation: -
Reference number: 1296

Savard, et al. Impact of Bifidobacterium animalis subsp. lactis BB-12 and, Lactobacillus acidophilus LA-5-containing yoghurt, on fecal bacterial counts of healthy adults. Int.J.Food Microbiol. 2011

Abstract: This randomized, placebo-controlled, double blind, parallel dose-response study investigated the impact of 4-week commercial yoghurt consumption supplemented with Bifidobacterium animalis subsp. lactis (BB-12) and Lactobacillus acidophilus (LA-5) on fecal bacterial counts of healthy adults. Fifty-eight volunteers were randomly assigned to three different groups: 1. placebo (no probiotic, no starter and no green tea extract); 2. Yoptimal (10(9)cfu/100g of BB-12 and LA-5 and 40mg of green tea extract) and 3. Yoptimal-10 (10(10)cfu/100g of BB-12, 10(9)cfu/100g of LA-5 and 40mg of green tea extract). These yoghurt products also contained Lactobacillus delbrueckii subsp. bulgaricus (10(7)cfu/100g) and Streptococcus thermophilus (10(10)cfu/100g). The quantitative PCR (qPCR) results showed that there were significant increases (P=0.02) in bifidobacteria counts with the Yoptimal treatment as compared to baseline. The fecal numbers of B. animalis subsp. lactis and LA-5 significantly increased in the two probiotic treatments compared to the placebo treatment. Viable counts of fecal lactobacilli were significantly higher (P=0.05) and those of enterococci were significantly lower (P=0.04) after the intervention when compared to placebo. No significant difference was observed between treatments in volunteers' weight, waist girth, blood pressure, fasting plasma triglyceride and HDL-C concentrations, as well as cholesterol/HDL-cholesterol ratio. However, a significant increase in plasma cholesterol levels was observed in the placebo group (P=0.0018) but the levels remained stable in the two probiotic yoghurt groups. These results show that probiotic strains supplemented in the form of yoghurt remain active during gut transit and are associated with an increase in beneficial bacteria and a reduction in potentially pathogenic bacteria. This trial was registered at clinicaltrials.gov as NCT00730626.

Chr. Hansen A/S -10-12 Bøge Allé – DK-2970 Hørsholm, Denmark - Phone: +45 45 74 74 74 - Fax: +45 45 74 88 88 - www.chr-hansen.com

The information contained herein is to the best of our knowledge true and correct and presented in good faith. It may be subject to change without further notice. To the best of our knowledge this product does not infringe Intellectual Property Rights of any third party. This information is offered solely for your consideration and verification. Copyright© Chr. Hansen A/S. All rights reserved.

CHR HANSEN

Research field: Other
Research subfield: Oral health
Study type: Human study
Probiotic strain: B. animalis subsp. lactis BB-12 and L. acidophilus LA-5
Dosage CFU/day: -
Product formulation: Other
Reference number: 1304

Singh, et al. Salivary mutans streptococci and lactobacilli modulations in young children on consumption of probiotic ice-cream containing Bifidobacterium lactis Bb12 and Lactobacillus acidophilus La5. Acta Odontol.Scand. 2011

Abstract: Abstract Objectives. To compare the levels of mutans streptococci and lactobacilli in saliva of school children, before and after consumption of probiotic and control ice-cream. Materials and methods. A double-blind, cross-over, placebo-controlled trial was carried out in forty, 12-14 year-old children, with no clinically detectable caries. The selected children were randomized equally into two groups I and II. Following an initial run-in period of 1 week, children in group I and II were given ice-creams 'A' and 'B', respectively, for 10 days. Being a cross-over study, the ice-creams were interchanged in the two groups after a 2-week wash-out period. Saliva samples at baseline and follow-up were assessed using Dentocult SM and Dentocult LB kits. Results. On statistical evaluation, it was seen that probiotic ice-cream brought about a statistically significant reduction (p-value = 0.003) in salivary mutans streptococci levels with no significant effect on lactobacilli levels. Conclusion. In conclusion, probiotic ice-cream containing Bifidobacterium lactis Bb-12 ATCC27536 and Lactobacillus acidophilus La-5 can reduce the levels of certain caries-associated micro-organisms in saliva.

The information contained herein is to the best of our knowledge true and correct and presented in good faith. It may be subject to change without further notice. To the best of our knowledge this product does not infringe Intellectual Property Rights of any third party. This information is offered solely for your consideration and verification. Copyright© Chr. Hansen A/S. All rights reserved.

Research field: Immune Health
Research subfield: Infections
Study type: Human study
Probiotic strain: B. animalis subsp. lactis BB-12
Dosage CFU/day: 10 billion
Product formulation: Other
Reference number: 1213

Taipale, et al. Bifidobacterium animalis subsp. lactis BB-12 in reducing the risk of infections in infancy. Br.J.Nutr. 2011;105:409-16

Abstract: The impact of controlled administration of Bifidobacterium animalis subsp. lactis BB-12 (BB-12) on the risk of acute infectious diseases was studied in healthy newborn infants. In this double-blind, placebo-controlled study, 109 newborn 1-month-old infants were assigned randomly to a probiotic group receiving a BB-12-containing tablet (n 55) or to a control group receiving a control tablet (n 54). Test tablets were administered to the infants twice a day (daily dose of BB-12 10 billion colony-forming units) from the age of 1-2 months to 8 months with a novel slow-release pacifier or a spoon. Breastfeeding habits, pacifier use, dietary habits, medications and all signs and symptoms of acute infections were registered. At the age of 8 months, faecal samples were collected for BB-12 determination (quantitative PCR method). The baseline characteristics of the two groups were similar, as was the duration of exclusive breastfeeding. BB-12 was recovered (detection limit log 5) in the faeces of 62 % of the infants receiving the BB-12 tablet. The daily duration of pacifier sucking was not associated with the occurrence of acute otitis media. No significant differences between the groups were observed in reported gastrointestinal symptoms, otitis media or use of antibiotics. However, the infants receiving BB-12 were reported to have experienced fewer respiratory infections (65 v. 94 %; risk ratio 0.69; 95 % CI 0.53, 0.89; P = 0.014) than the control infants. Controlled administration of BB-12 in early childhood may reduce respiratory infections.

The information contained herein is to the best of our knowledge true and correct and presented in good faith. It may be subject to change without further notice. To the best of our knowledge this product does not infringe Intellectual Property Rights of any third party. This information is offered solely for your consideration and verification. Copyright© Chr. Hansen A/S. All rights reserved.

CHR HANSEN

Research field: Immune Health
Research subfield: Atopic diseases
Study type: Human study
Probiotic strain: B. animalis subsp. lactis BB-12 and L. acidophilus LA-5 and L. rhamnosus LGG
Dosage CFU/day: -
Product formulation: Fermented milk
Reference number: 0855

Dotterud, et al. Probiotics in pregnant women to prevent allergic disease: a randomized, double-blind trial. Br.J.Dermatol. 2010;163

Abstract: Summary Background Previous reports have suggested that certain probiotics given to mothers and children at risk of atopy halves the incidence of atopic dermatitis (AD) at 2 years of age. Objectives To examine if probiotics given to pregnant women in a nonselected population could prevent atopic sensitization or allergic diseases during the child's first 2 years. Methods In a randomized, double-blind trial of children from a nonselected maternal population (ClinicalTrials.gov identifier: NCT00159523), women received probiotic milk or placebo from 36 weeks of gestation to 3 months postnatally during breastfeeding. The probiotic milk contained Lactobacillus rhamnosus GG, L. acidophilus La-5 and Bifidobacterium animalis subsp. lactis Bb-12. Children with an itchy rash for more than 4 weeks were assessed for AD. At 2 years of age, all children were assessed for atopic sensitization, AD, asthma and allergic rhinoconjunctivitis. The intention-to-treat (ITT) analysis was enabled by multiple imputations. Results Four hundred and fifteen pregnant women were computer randomized. At 2 years, 138 and 140 children in the probiotic and the placebo groups, respectively, were assessed. In the ITT analysis, the odds ratio (OR) for the cumulative incidence of AD was 0.51 in the probiotic group compared with the placebo [95% confidence interval (CI) 0.30-0.87; P = 0.013]. There were no significant effects on asthma (OR 0.68, 95% CI 0.26-1.80; P = 0.437) or atopic sensitization (OR 1.52, 95% CI 0.74-3.14; P = 0.254). Conclusions Probiotics given to nonselected mothers reduced the cumulative incidence of AD, but had no effect on atopic sensitization.

The information contained herein is to the best of our knowledge true and correct and presented in good faith. It may be subject to change without further notice. To the best of our knowledge this product does not infringe Intellectual Property Rights of any third party. This information is offered solely for your consideration and verification. Copyright© Chr. Hansen A/S. All rights reserved.

CHR HANSEN

Research field: Weight Management
Research subfield: Weight management
Study type: Human study
Probiotic strain: B. animalis subsp. lactis BB-12 and L. rhamnosus LGG
Dosage CFU/day: -
Product formulation: -
Reference number: 0960

Ilmonen, et al. Impact of dietary counselling and probiotic intervention on maternal anthropometric measurements during and after pregnancy: A randomized placebo-controlled trial. Clin.Nutr. 2010

Abstract: BACKGROUND & AIMS: To establish whether probiotic supplemented dietary counselling influences maternal anthropometric measurements during and after pregnancy.METHODS: At the first trimester of pregnancy 256 women were randomly assigned to receive nutrition counselling to modify dietary intake according to current recommendations or as controls; dietary intervention groups were further randomized to receive probiotics Lactobacillus rhamnosus GG (ATCC 53103) and Bifidobacterium lactis (diet/probiotics) or placebo (diet/placebo) capsules in a double-blind manner, whilst the controls received placebo (control/placebo). The intervention lasted until the end of exclusive breastfeeding for up to six months.RESULTS: The risk of central adiposity defined as waist circumference 80 cm or more was lowered in women in the diet/probiotics group compared with the control/placebo group (OR 0.30, 95%CI 0.11-0.85, p = 0.023 adjusted for baseline BMI), whilst the diet/placebo group did not differ from the controls (OR 1.00, 95% CI 0.38-2.68, p = 0.994) at 6 months postpartum. The number needed to treat (NNT) with diet/probiotics to prevent one woman from developing a waist circumference of 80 cm or more was 4. Healthy eating pattern at 12 months postpartum (p = 0.001) and BMI prior to pregnancy (p < 0.001) were strong determinants of BMI at 12 months postpartum when adjusted for dietary intervention and exercise.CONCLUSION: The impact of probiotics-supplemented dietary counselling on central adiposity, may offer a novel means for the prevention and management of obesity. This trial was registered at clinicaltrials.gov as NCT 00167700, section 3.

The information contained herein is to the best of our knowledge true and correct and presented in good faith. It may be subject to change without further notice. To the best of our knowledge this product does not infringe Intellectual Property Rights of any third party. This information is offered solely for your consideration and verification. Copyright© Chr. Hansen A/S. All rights reserved.

CHR HANSEN

Research field: Other
Research subfield: Growth
Study type: Human study
Probiotic strain: B. animalis subsp. lactis BB-12 and L. rhamnosus LGG
Dosage CFU/day: -
Product formulation: -
Reference number: 1043

Luoto, et al. Impact of maternal probiotic-supplemented dietary counselling on pregnancy outcome and prenatal and postnatal growth: a double-blind, placebo-controlled study. Br.J.Nutr. 2010;103(12):1792-1799

Abstract: The perinatal nutritional environment impacts upon the health and well-being of mother and child also in the long term. The aim of the present study was to determine the safety and efficacy of perinatal probiotic-supplemented dietary counselling by evaluating pregnancy outcome and fetal and infant growth during the 24 months' follow-up. Altogether, 256 women were randomised at their first trimester of pregnancy into a control and a dietary intervention group. The intervention group received intensive dietary counselling provided by a nutritionist and were further randomised, double-blind to receive probiotics (Lactobacillus rhamnosus GG and Bifidobacterium lactis Bb12; diet/probiotics) or placebo (diet/placebo). Firstly, probiotic intervention reduced the frequency of gestational diabetes mellitus (GDM); 13 % (diet/probiotics) v. 36 % (diet/placebo) and 34 % (control); P = 0.003. Secondly, the safety of this approach was attested by normal duration of pregnancies with no adverse events in mothers or children. No significant differences in prenatal or postnatal growth rates among the study groups were detected. Thirdly, distinctive effects of the two interventions were detected; probiotic intervention reduced the risk of GDM and dietary intervention diminished the risk of larger birth size in affected cases; P = 0.035 for birth weight and P = 0.028 for birth length. The results of the present study show that probiotic-supplemented perinatal dietary counselling could be a safe and cost-effective tool in addressing the metabolic epidemic. In view of the fact that birth size is a risk marker for later obesity, the present results are of significance for public health in demonstrating that this risk is modifiable.

The information contained herein is to the best of our knowledge true and correct and presented in good faith. It may be subject to change without further notice. To the best of our knowledge this product does not infringe Intellectual Property Rights of any third party. This information is offered solely for your consideration and verification. Copyright© Chr. Hansen A/S. All rights reserved.

Research field: Other
Research subfield: Glucose regulation
Study type: Human study
Probiotic strain: B. animalis subsp. lactis BB-12 and L. rhamnosus LGG
Dosage CFU/day: -
Product formulation: -
Reference number: 1012

Laitinen, et al. Probiotics and dietary counselling contribute to glucose regulation during and after pregnancy: a randomised controlled trial. Br.J.Nutr. 2009;101(11):1679-1687

Abstract: Balanced glucose metabolism ensures optimal fetal growth with long-term health implications conferred on both mother and child. We examined whether supplementation of probiotics with dietary counselling affects glucose metabolism in normoglycaemic pregnant women. At the first trimester of pregnancy 256 women were randomised to receive nutrition counselling to modify dietary intake according to current recommendations or as controls; the dietary intervention group was further randomised to receive probiotics (Lactobacillus rhamnosus GG and Bifidobacterium lactis Bb12; diet/probiotics) or placebo (diet/placebo) in a double-blind manner, whilst the control group received placebo (control/placebo). Blood glucose concentrations were lowest in the diet/probiotics group during pregnancy (baseline-adjusted means 4.45, 4.60 and 4.56 mmol/l in diet/probiotics, diet/placebo and control/placebo, respectively; P = 0.025) and over the 12 months' postpartum period (baseline-adjusted means 4.87, 5.01 and 5.02 mmol/l; P = 0.025). Better glucose tolerance in the diet/probiotics group was confirmed by a reduced risk of elevated glucose concentration compared with the control/placebo group (OR 0.31 (95 % CI 0.12, 0.78); P = 0.013) as well as by the lowest insulin concentration (adjusted means 7.55, 9.32 and 9.27 mU/l; P = 0.032) and homeostasis model assessment (adjusted means 1.49, 1.90 and 1.88; P = 0.028) and the highest quantitative insulin sensitivity check index (adjusted means 0.37, 0.35 and 0.35; P = 0.028) during the last trimester of pregnancy. The effects observed extended over the 12-month postpartum period. The present study demonstrated that improved blood glucose control can be achieved by dietary counselling with probiotics even in a normoglycaemic population and thus may provide potential novel means for the prophylactic and therapeutic management of glucose disorders.

Chr. Hansen A/S -10-12 Bøge Allé – DK-2970 Hørsholm, Denmark - Phone: +45 45 74 74 74 - Fax: +45 45 74 88 88 - www.chr-hansen.com

The information contained herein is to the best of our knowledge true and correct and presented in good faith. It may be subject to change without further notice. To the best of our knowledge this product does not infringe Intellectual Property Rights of any third party. This information is offered solely for your consideration and verification. Copyright© Chr. Hansen A/S. All rights reserved.

Research field: Immune Health
Research subfield: Infections
Study type: Human study
Probiotic strain: B. animalis subsp. lactis BB-12 and L. rhamnosus LGG
Dosage CFU/day: 10 billion of each
Product formulation: Milk powder
Reference number: 0494

Rautava, et al. Specific probiotics in reducing the risk of acute infections in infancy--a randomised, double-blind, placebo-controlled study. Br.J.Nutr. 2009;101(11):1722-1726

Abstract: A randomised, double-blind, placebo-controlled study was conducted to determine whether probiotics might be effective in reducing the risk of infections in infancy. Infants requiring formula before the age of 2 months were recruited from community well-baby clinics. Infant formula supplemented with the probiotics Lactobacillus rhamnosus GG and Bifidobacterium lactis Bb-12 or placebo was administered daily until the age of 12 months. Incidence of early infections (before the age of 7 months) and incidence of recurrent (three or more) infections during the first year of life were recorded as the main outcome measures of the study. During the first 7 months of life, seven out of thirty-two (22 %) infants receiving probiotics and twenty out of forty (50 %) infants receiving placebo experienced acute otitis media (risk ratio (RR) 0.44 (95 % CI 0.21, 0.90); P = 0.014) and antibiotics were prescribed for ten out of thirty-two (31 %) infants receiving probiotics and twenty-four out of forty (60 %) infants receiving placebo (RR 0.52 (95 % CI 0.29, 0.92); P = 0.015). During the first year of life, nine out of thirty-two (28 %) infants receiving probiotics and twenty-two out of forty (55 %) infants receiving placebo encountered recurrent respiratory infections (RR 0.51 (95 % CI 0.27, 0.95); P = 0.022). These data suggest that probiotics may offer a safe means of reducing the risk of early acute otitis media and antibiotic use and the risk of recurrent respiratory infections during the first year of life. Further clinical trials are warranted.

Chr. Hansen A/S -10-12 Bøge Allé – DK-2970 Hørsholm, Denmark - Phone: +45 45 74 74 74 - Fax: +45 45 74 88 88 - www.chr-hansen.com

The information contained herein is to the best of our knowledge true and correct and presented in good faith. It may be subject to change without further notice. To the best of our knowledge this product does not infringe Intellectual Property Rights of any third party. This information is offered solely for your consideration and verification. Copyright© Chr. Hansen A/S. All rights reserved.

CHR HANSEN

Research field: Other
Research subfield: Chronic fatigue syndrome
Study type: Human study
Probiotic strain: B. animalis subsp. lactis BB-12 and L. paracasei subsp. paracasei F19
Dosage CFU/day: 40 billion
Product formulation: -
Reference number: 0720

Sullivan, et al. Effect of supplement with lactic-acid producing bacteria on fatigue and physical activity in patients with chronic fatigue syndrome. Nutr.J. 2009

Abstract: Disturbances in intestinal microbial ecology and in the immune system of the host have been implicated as a part of the pathogenesis in chronic fatigue syndrome. Probiotic lactic acid producing bacteria have been shown to prevent and alleviate gastrointestinal disturbances and to normalize the cytokine profile which might be of an advantage for patients suffering from chronic fatigue syndrome. The aim of the study was to evaluate the effect of Lactobacillus paracasei ssp. paracasei F19, Lactobacillus acidophilus NCFB 1748 and Bifidobacterium lactis Bb12 on fatigue and physical activity in CFS patients. Fifteen patients fulfilling the criteria set by international researchers in the field at the US Centre for Disease Control and Prevention in 1994 for chronic fatigue syndrome, were included in the study. The patients had high fatigue severity scores and high disability scores. During the first two weeks baseline observations without treatment were assessed, succeeded by four weeks of intake of a probiotic product and a four-week follow-up period. The fatigue, health and physical activity was assessed by the use of the Visual Analogue Scales and the SF-12 Health Survey. Faecal samples were collected and the normal microflora was analysed. Neurocognitive functions improved during the study period while there were no significant changes in fatigue and physical activity scores. No major changes occurred in the gastrointestinal microflora. At the end of the study 6 of 15 patients reported that they had improved according to the assessment described. The findings in this study that improvement of health is possible to achieve should encourage further studies with interventions with probiotics in patients with CFS.

The information contained herein is to the best of our knowledge true and correct and presented in good faith. It may be subject to change without further notice. To the best of our knowledge this product does not infringe Intellectual Property Rights of any third party. This information is offered solely for your consideration and verification. Copyright© Chr. Hansen A/S. All rights reserved.

CHR HANSEN

Research field: Other
Research subfield: Safety
Study type: Human study
Probiotic strain: B. animalis subsp. lactis BB-12 and L. paracasei subsp. paracasei L. casei 431
Dosage CFU/day: -
Product formulation: -
Reference number: 1253

Vlieger, et al. Tolerance and safety of Lactobacillus paracasei ssp. paracasei in combination with Bifidobacterium animalis ssp. lactis in a prebiotic-containing infant formula: a randomised controlled trial. Br.J.Nutr. 2009;102:869-875

Abstract: The addition of probiotics to infant formula has been shown to be an efficient way to increase the number of beneficial bacteria in the intestine in order to promote a gut flora resembling that of breast-fed infants. The objective of the present study was to evaluate the safety and tolerance of a combination of two probiotic strains in early infancy. A group of 126 newborns were randomised to receive a prebiotic-containing starter formula supplemented with Lactobacillus paracasei ssp. paracasei and Bifidobacterium animalis ssp. lactis or the same formula without probiotics for the first 3 months of life. A total of eighty infants continued the study until they were aged 6 months. Growth measurements were taken monthly at healthy baby clinics. Diaries were used to monitor behaviour, infections, use of antibiotics, as well as stool characteristics. Normal growth occurred in all infants and no statistically significant differences were detected between the probiotics group and the control group for gain in weight, length and head circumference. Infants in the probiotics group produced softer and more frequent stools during the first 3 months of life. No differences were found in crying and sleeping hours, number of parent-diagnosed infections, antibiotic use, visits to the general practitioner and number of adverse events. The use of a prebiotic-containing starter formula supplemented with L. paracasei ssp. paracasei and B. animalis ssp. lactis in early infancy is safe, well tolerated and has no adverse effects on growth and infant behaviour.

The information contained herein is to the best of our knowledge true and correct and presented in good faith. It may be subject to change without further notice. To the best of our knowledge this product does not infringe Intellectual Property Rights of any third party. This information is offered solely for your consideration and verification. Copyright© Chr. Hansen A/S. All rights reserved.

Research field: Other
Research subfield: Blood pressure
Study type: Human study
Probiotic strain: B. animalis subsp. lactis BB-12 and L. rhamnosus LGG
Dosage CFU/day: 1 billion
Product formulation: Fermented milk
Reference number: 0690

Aaltonen, et al. Evidence of infant blood pressure programming by maternal nutrition during pregnancy: a prospective randomized controlled intervention study. J.Pediatr. 2008;152:79-84

Abstract: OBJECTIVES: To evaluate the impact of maternal nutrition during pregnancy on infant blood pressure. STUDY DESIGN: Pregnant women (n = 256) were randomized into 3 groups: modified dietary intake according to current recommendations and probiotics (diet/probiotics), placebo (diet/placebo), and a control/placebo group. In the infants born to these women, blood pressure was recorded at age 6 months using an automated oscillometric DINAMAP R. RESULTS: Despite significant differences in maternal dietary intakes between the study groups, the intervention focusing on maternal fat intake showed no direct impact on infants' blood pressure. Instead, a complex U-shaped interrelationship was uncovered; the highest and lowest quartiles of intakes of specific nutrients, carbohydrate (P = .006 for systolic pressure and P = .015 for diastolic pressure), and monounsaturated fatty acids (P = .029 for diastolic pressure) compared with the middle quartiles resulted in higher blood pressure at age 6 months. The pattern between maternal carbohydrate intake during pregnancy and infants' blood pressure remained significant even after adjustment for breastfeeding and body length. A reverse U-shaped trend again was observed between maternal intake of fruits and infants' systolic blood pressure (P = .077). CONCLUSION: With a view toward programming blood pressure to adulthood, our results suggest an opportunity for dietary counseling to promote child health.

The information contained herein is to the best of our knowledge true and correct and presented in good faith. It may be subject to change without further notice. To the best of our knowledge this product does not infringe Intellectual Property Rights of any third party. This information is offered solely for your consideration and verification. Copyright© Chr. Hansen A/S. All rights reserved.

CHR HANSEN

Research field: Oral Health
Research subfield: Caries
Study type: Human study
Probiotic strain: B. animalis subsp. lactis BB-12
Dosage CFU/day: -
Product formulation: Fermented milk
Reference number: 0800

Caglar, et al. Short-term effect of ice-cream containing Bifidobacterium lactis Bb-12 on the number of salivary mutans streptococci and lactobacilli. Acta Odontol.Scand. 2008;66:154-158

Abstract: OBJECTIVE: Probiotic bacteria are thought to reduce the risk of some infectious diseases. The aim of the present study was to examine whether or not short-term consumption of ice-cream containing bifidobacteria can affect the salivary levels of mutans streptococci and lactobacilli in young adults. MATERIAL AND METHODS: A double-blind, randomized crossover study was performed and 24 healthy subjects (mean age 20 years) were followed over 4 periods. During periods 2 and 4 (10 days each), they ingested 100 ml (53 g) ice-cream containing Bifidobacterium lactis Bb-12 once daily or a control ice-cream without viable bacteria. Periods 1 and 3 were run-in and washout periods, respectively. Salivary mutans streptococci and lactobacilli were enumerated with chair-side kits at baseline and immediately after the intervention period. RESULTS: A statistically significant reduction ($p<0.05$) of salivary mutans streptococci was recorded after consumption of the probiotic ice-cream. A decline of high mutans streptococci counts was also seen after intake of the control ice-cream, but the difference compared to baseline was not statistically significant. The salivary lactobacilli levels were unaltered after both regimes. CONCLUSIONS: Daily consumtion of ice-cream containing probiotic bifidobacteria may reduce the salivary levels of mutans streptococci in young adults.

Chr. Hansen A/S -10-12 Bøge Allé – DK-2970 Hørsholm, Denmark - Phone: +45 45 74 74 74 - Fax: +45 45 74 88 88 - www.chr-hansen.com

The information contained herein is to the best of our knowledge true and correct and presented in good faith. It may be subject to change without further notice. To the best of our knowledge this product does not infringe Intellectual Property Rights of any third party. This information is offered solely for your consideration and verification. Copyright© Chr. Hansen A/S. All rights reserved.

CHR HANSEN

Research field: Gastrointestinal Health
Research subfield: Irritable bowel syndrome
Study type: Human study
Probiotic strain: B. animalis subsp. lactis
Dosage CFU/day: 2.4 billion
Product formulation: -
Reference number: 0696

Kajander, et al. Clinical trial: multispecies probiotic supplementation alleviates the symptoms of irritable bowel syndrome and stabilizes intestinal microbiota. Aliment.Pharmacol.Ther. 2008;27:48-57

Abstract: BACKGROUND: Irritable bowel syndrome is the most common diagnosis in gastroenterology. Trials suggest certain probiotics to be beneficial. AIM: To investigate the effects of multispecies probiotic supplementation (Lactobacillus rhamnosus GG, L. rhamnosus Lc705, Propionibacterium freudenreichii ssp. shermanii JS and Bifidobacterium animalis ssp. lactis Bb12) on abdominal symptoms, quality of life, intestinal microbiota and inflammatory markers in irritable bowel syndrome. METHODS: Eighty-six irritable bowel syndrome patients (Rome II criteria) participated in this randomized, placebo-controlled 5-month intervention. Patients were randomized to receive daily either multispecies probiotic supplementation or placebo. Irritable bowel syndrome symptoms, quality of life, microarray-based intestinal microbiota stability (n = 20), serum cytokines and sensitive C-reactive protein were monitored. RESULTS: The composite irritable bowel syndrome score had at 5 months decreased 14 points (95% CI: -19 to -9) from baseline with the multispecies probiotic vs. three points (95% CI: -8 to 1) with placebo (P = 0.0083). Especially, distension and abdominal pain were affected. A stabilization of the microbiota was observed, as the microbiota similarity index increased with the probiotic supplementation (1.9 +/- 3.1), while it decreased with placebo (-2.9 +/- 1.7). No differences were seen in C-reactive protein. CONCLUSIONS: This multispecies probiotic seems to be an effective and safe option to alleviate symptoms of irritable bowel syndrome, and to stabilize the intestinal microbiota.

The information contained herein is to the best of our knowledge true and correct and presented in good faith. It may be subject to change without further notice. To the best of our knowledge this product does not infringe Intellectual Property Rights of any third party. This information is offered solely for your consideration and verification. Copyright© Chr. Hansen A/S. All rights reserved.

CHR HANSEN

Research field: Immune Health
Research subfield: Inflammation
Study type: Human study
Probiotic strain: B. animalis subsp. lactis BB-12 and L. rhamnosus LGG
Dosage CFU/day: -
Product formulation: -
Reference number: 0985

Kekkonen, et al. Probiotic intervention has strain-specific anti-inflammatory effects in healthy adults. World J.Gastroenterol. 2008;14(13):2029-2036

Abstract: AIM: To evaluate the effects of three potentially anti-inflammatory probiotic bacteria from three different genera on immune variables in healthy adults in a clinical setting based on previous in vitro characterization of cytokine responses. METHODS: A total of 62 volunteers participated in this randomized, double-blind and placebo-controlled parallel group intervention study. The volunteers were randomized to receive a milk-based drink containing either Lactobacillus rhamnosus GG (LGG), Bifidobacterium animalis ssp. lactis Bb12 (Bb12), or Propionibacterium freudenreichii ssp. shermanii JS (PJS) or a placebo drink for 3 wk. Venous blood and saliva samples were taken at baseline and on d 1, 7 and 21. Fecal samples were collected at baseline and at the end of intervention. RESULTS: The serum hsCRP expressed as the median AUC(0-21) (minus baseline) was 0.018 mg/L in the placebo group, -0.240 mg/L in the LGG group, 0.090 mg/L in the Bb12 group and -0.085 mg/L in the PJS group (P = 0.014). In vitro production of TNF-alpha from in vitro cultured peripheral blood mononuclear cells (PBMC) was significantly lower in subjects receiving LGG vs placebo. IL-2 production from PBMC in the Bb12 group was significantly lower compared with the other groups. CONCLUSION: In conclusion, probiotic bacteria have strain-specific anti-inflammatory effects in healthy adults.

Chr. Hansen A/S -10-12 Bøge Allé – DK-2970 Hørsholm, Denmark - Phone: +45 45 74 74 74 - Fax: +45 45 74 88 88 - www.chr-hansen.com

The information contained herein is to the best of our knowledge true and correct and presented in good faith. It may be subject to change without further notice. To the best of our knowledge this product does not infringe Intellectual Property Rights of any third party. This information is offered solely for your consideration and verification. Copyright© Chr. Hansen A/S. All rights reserved.

CHR HANSEN

Research field: Other
Research subfield: Diarrhea
Study type: Human study
Probiotic strain: B. animalis subsp. lactis BB-12 and L. acidophilus LA-5 and L. rhamnosus LGG
Dosage CFU/day: 25 billion LGG, 2.5 billion LA-5, 25 billion BB-12
Product formulation: Fermented milk
Reference number: 0627

Wenus, et al. Prevention of antibiotic-associated diarrhoea by a fermented probiotic milk drink. Eur.J.Clin.Nutr. 2008;62:299-301

Abstract: OBJECTIVE: To study the preventive effect of a milk drink fermented with multistrain probiotics on antibiotic associated diarrhoea (AAD). DESIGN: Double-blind placebo controlled study. SETTING: University Hospital of North Norway. SUBJECTS AND METHODS: Of 853 patients treated with antibiotics, 87 met the inclusion criteria, and were randomized to ingestion of a fermented milk drink containing LGG, La-5 and Bb-12 (n=46) or placebo with heat-killed bacteria (n=41), during a period of 14 days. A diary was recorded, and stool samples were collected for microbiological analyses. RESULTS: Sixty-three patients completed the study according to the protocol; two patients (5.9%) in the treatment group and eight (27.6%) in the placebo group developed AAD (P=0.035). The relative risk of developing AAD was 0.21 (95% confidence interval: 0.05-0.93) when given probiotic milk drink. CONCLUSION: A fermented multistrain probiotic milk drink may prevent four of five cases of AAD in adult hospitalized patients. SPONSORSHIP: TINE BA, Oslo, Norway.

The information contained herein is to the best of our knowledge true and correct and presented in good faith. It may be subject to change without further notice. To the best of our knowledge this product does not infringe Intellectual Property Rights of any third party. This information is offered solely for your consideration and verification. Copyright© Chr. Hansen A/S. All rights reserved.

CHR HANSEN

Research field: Other
Research subfield: Other
Study type: Human study
Probiotic strain: B. animalis subsp. lactis
Dosage CFU/day: 2 billion
Product formulation: -
Reference number: 0689

Kaplas, et al. Dietary counseling and probiotic supplementation during pregnancy modify placental phospholipid fatty acids. Lipids 2007;42:865-870

Abstract: It has previously been shown that maternal nutrition affects the fetal environment, with consequences for the infant's health. From early pregnancy onwards participants here received a combination of dietary counseling and probiotics (Lactobacillus GG and Bifidobacterium lactis Bb12; n = 10), dietary counseling with placebo (n = 12), or placebo alone (n = 8). The major differences in placental fatty acids were attributable to a higher concentration of n-3 polyunsaturated fatty acids in both intervention arms than in controls. Further, dietary counseling with probiotics resulted in higher concentrations of linoleic (18:2n-6) and dihomo-gamma-linolenic acids (20:3n-6) compared with dietary counseling with placebo or controls.

The information contained herein is to the best of our knowledge true and correct and presented in good faith. It may be subject to change without further notice. To the best of our knowledge this product does not infringe Intellectual Property Rights of any third party. This information is offered solely for your consideration and verification. Copyright© Chr. Hansen A/S. All rights reserved.

CHR HANSEN

Research field: Immune Health
Research subfield: Atopic eczema
Study type: Human study
Probiotic strain: B. animalis subsp. lactis BB-12
Dosage CFU/day: 5.2 billion
Product formulation: Fermented milk
Reference number: 0546

Matsumoto, et al. LKM512 yogurt consumption improves the intestinal environment and induces the T-helper type 1 cytokine in adult patients with intractable atopic dermatitis. Clin.Exp.Allergy 2007;37:358-370

Abstract: BACKGROUND: In atopic dermatitis (AD) patients, the intestinal mucosal barrier function is weakened, permiting frequent invasion by antigens. Polyamines and short-chain fatty acids (SCFA) produced by intestinal bacteria are involved in the promotion of intestinal mucosal barrier functions. AIM: Our aim was to investigate the effect of pro-biotic yogurt containing Bifidobacterium animalis subsp. lactis LKM512 (LKM512 yogurt) on subjective symptoms, intestinal microbiota, intestinal bacterial metabolites (polyamines and SCFA), and T-helper type 1 (Th1)/Th2 balance in intractable AD patients. METHODS: In a double-blind, placebo-controlled, crossover study, LKM512 yogurt was given for 4 weeks to 10 adult AD patients who were diagnosed with moderate AD (four males and six females; average age, 22.1 years). The subjective symptoms were recorded after each intervention. The dynamics of fecal microbiota were analysed by the terminal-restriction fragment length polymorphism method. The effects of LKM512 yogurt on fecal polyamines, SCFA, and serum cytokines were evaluated. RESULTS: Scores of itch and burning tended to improve to a greater extent by LKM512 yogurt consumption than by placebo consumption. LKM512 yogurt ($P<0.005$) and placebo consumption ($P<0.05$) significantly increased the serum IFN-gamma concentration by six- and threefold, respectively. Fecal microbiota was altered dynamically by LKM512 yogurt consumption, in particular, the bacterial species and phylotypes of Bifidobacterium, Clostridium cluster IV and subcluster XIVa were increased in number. In addition, fecal spermidine concentration was significantly ($P<0.05$) increased, while fecal butyrate also tended to be increased by LKM512 yogurt consumption. CONCLUSION: We conclude that LKM512 yogurt consumption may be effective against intractable adult-type AD and this effect depends on the recovery of the intestinal mucosal barrier function and the induction of the Th1-type cytokine by polyamines and SCFA, particularly, butyrate, produced by the altered intestinal microbiota.

The information contained herein is to the best of our knowledge true and correct and presented in good faith. It may be subject to change without further notice. To the best of our knowledge this product does not infringe Intellectual Property Rights of any third party. This information is offered solely for your consideration and verification. Copyright© Chr. Hansen A/S. All rights reserved.

CHR HANSEN

Research field: Gastrointestinal Health
Research subfield: H. pylori
Study type: Human study
Probiotic strain: B. animalis subsp. lactis BB-12 and L. rhamnosus LGG and L. rhamnosus LC705 and P. freudenreichii JS
Dosage CFU/day: 2.5 billion
Product formulation: -
Reference number: 0634

Myllyluoma, et al. Probiotic intervention decreases serum gastrin-17 in Helicobacter pylori infection. Dig.Liver Dis. 2007;39:516-523

Abstract: BACKGROUND: Previously we showed that a probiotic combination with L. rhamnosus GG was beneficial as an adjuvant therapy during H. pylori eradication. AIM: To evaluate whether probiotic combination with LGG adheres to the upper gastrointestinal mucosa and modifies H. pylori colonisation and H. pylori induced inflammation. METHODS: Thirteen patients referred for gastroduodenoscopy received a drink consisting of equal doses (2.5x10(9)CFU) of LGG, L. rhamnosus LC705, Propionibacterium freudenreichii JS and Bifidobacterium lactis Bb12 daily. Recovery of probiotics in biopsies (antrum, corpus, duodenum) and faecal samples was evaluated by strain-specific quantitative polymerase chain reaction. H. pylori colonization and gastric inflammation was investigated by urease activity ((13)C-urea breath test), histology and serum pepsinogen I, II and gastrin-17 measurements. RESULTS: Twelve patients were fully investigated; of these three of the patients had LGG adhering to the biopsies at end of the intervention. Other probiotic strains were not detected, even though the recovery of all individual probiotic strains from the faeces was significantly increased (p<0.01). After the treatment, the level of (13)C-urea breath test (p=0.063) and gastrin-17 (p=0.046) decreased. CONCLUSIONS: The decreases in (13)C-urea breath test and gastrin-17 indicate that the probiotic combination exerts a beneficial effect on gastric mucosa in H. pylori infected patients. LGG showed marginal ability to adhere to the upper gastrointestinal tract mucosa.

The information contained herein is to the best of our knowledge true and correct and presented in good faith. It may be subject to change without further notice. To the best of our knowledge this product does not infringe Intellectual Property Rights of any third party. This information is offered solely for your consideration and verification. Copyright© Chr. Hansen A/S. All rights reserved.

Research field: Gastrointestinal Health
Research subfield: Irregular bowel movements
Study type: Human study
Probiotic strain: B. animalis subsp. lactis BB-12
Dosage CFU/day: 1 billion
Product formulation: Fermented milk
Reference number: 0653

Pitkala, et al. Fermented cereal with specific bifidobacteria normalizes bowel movements in elderly nursing home residents. A randomized, controlled trial. J.Nutr.Health Aging 2007;11:305-311

Abstract: OBJECTIVE: To assess how fermented oat drink with two selected Bifidobacterium longum strains influences bowel movements among elderly nursing home residents. DESIGN: A randomized, double-blind, placebo-controlled trial. SETTING: 12 wards in two nursing homes in Finland. PARTICIPANTS: 209 residents. INTERVENTION: Wards were randomized to receive daily a fermented oat drink with 1) 109 CFU/day Bifidobacterium longum strains or 2) 109 CFU/day Bifidobacterium lactis Bb12 or 3) without viable bacteria (placebo) for 7 months. MEASUREMENTS: Regularity of bowel movements (no movements or functioning) and consistency of stools (normal, soft or diarrhoea) were recorded for each resident on a daily basis. RESULTS: The fermented oat drinks were well taken by the subjects, compliance being 85%. The groups receiving active products had more frequent bowel movements than did the placebo group (B. longum group normal functioning 28.5% of follow-up days, B.lactis group 26.9%, and placebo group 20.0%, respectively). The differences between the B. longum and the placebo group (mean 7.1, 95% CI 2.3 - 11.9, p=0.004) and between the B.lactis group and the placebo (mean 6.7, 95% CI 2.5 - 10.9, p = 0.002) were significant even when diarrhoea and constipation in the 3 months prior to the study were used as covariates. Conclusions: It is possible to normalize bowel movements in frail nursing home.

Chr. Hansen A/S -10-12 Bøge Allé – DK-2970 Hørsholm, Denmark - Phone: +45 45 74 74 74 - Fax: +45 45 74 88 88 - www.chr-hansen.com

The information contained herein is to the best of our knowledge true and correct and presented in good faith. It may be subject to change without further notice. To the best of our knowledge this product does not infringe Intellectual Property Rights of any third party. This information is offered solely for your consideration and verification. Copyright© Chr. Hansen A/S. All rights reserved.

CHR HANSEN

Research field: Immune Health
Research subfield: Cancer
Study type: Human study
Probiotic strain: B. animalis subsp. lactis BB-12 and L. rhamnosus LGG
Dosage CFU/day: 10 billion of each
Product formulation: NA
Reference number: 0544

Rafter, et al. Dietary synbiotics reduce cancer risk factors in polypectomized and colon cancer patients. Am.J.Clin.Nutr. 2007;85:488-496

Abstract: BACKGROUND: Animal studies suggest that prebiotics and probiotics exert protective effects against tumor development in the colon, but human data supporting this suggestion are weak. OBJECTIVE: The objective was to verify whether the prebiotic concept (selective interaction with colonic flora of nondigested carbohydrates) as induced by a synbiotic preparation-oligofructose-enriched inulin (SYN1) + Lactobacillus rhamnosus GG (LGG) and Bifidobacterium lactis Bb12 (BB12)-is able to reduce the risk of colon cancer in humans. DESIGN: The 12-wk randomized, double-blind, placebo-controlled trial of a synbiotic food composed of the prebiotic SYN1 and probiotics LGG and BB12 was conducted in 37 colon cancer patients and 43 polypectomized patients. Fecal and blood samples were obtained before, during, and after the intervention, and colorectal biopsy samples were obtained before and after the intervention. The effect of synbiotic consumption on a battery of intermediate bio-markers for colon cancer was examined. RESULTS: Synbiotic intervention resulted in significant changes in fecal flora: Bifidobacterium and Lactobacillus increased and Clostridium perfringens decreased. The intervention significantly reduced colorectal proliferation and the capacity of fecal water to induce necrosis in colonic cells and improve epithelial barrier function in polypectomized patients. Genotoxicity assays of colonic biopsy samples indicated a decreased exposure to genotoxins in polypectomized patients at the end of the intervention period. Synbiotic consumption prevented an increased secretion of interleukin 2 by peripheral blood mononuclear cells in the polypectomized patients and increased the production of interferon gamma in the cancer patients. CONCLUSIONS: Several colorectal cancer biomarkers can be altered favorably by synbiotic intervention.

The information contained herein is to the best of our knowledge true and correct and presented in good faith. It may be subject to change without further notice. To the best of our knowledge this product does not infringe Intellectual Property Rights of any third party. This information is offered solely for your consideration and verification. Copyright© Chr. Hansen A/S. All rights reserved.

CHR HANSEN

Research field: Gastrointestinal Health
Research subfield: Barrier function
Study type: Human study
Probiotic strain: B. animalis subsp. lactis BB-12 and L. acidophilus LA-5 and L. bulgaricus LBY-27 and S. thermophilus STY-31
Dosage CFU/day: 12 billion
Product formulation: Other
Reference number: 0526

Reddy, et al. Randomized clinical trial of effect of synbiotics, neomycin and mechanical bowel preparation on intestinal barrier function in patients undergoing colectomy. Br.J.Surg. 2007;94:546-554

Abstract: BACKGROUND: The aim of this study was to investigate whether it is possible to modulate gut microflora and preserve intestinal barrier function during elective colorectal surgery by using combinations of oral antibiotics, synbiotics and mechanical bowel preparation (MBP). METHODS: Ninety-two patients were randomly assigned to one of four groups. Group 1 had MBP only, group 2 had neomycin + MBP, group 3 had synbiotics + neomycin + MBP, and group 4 had synbiotics + neomycin but no MBP. Changes in gut microflora were assessed by culturing nasogastric aspirates and polymerase chain reaction-denaturing gradient gel electrophoresis of faecal samples. Intestinal barrier function was determined by microbiological confirmation of bacterial translocation and measurement of intestinal permeability. The inflammatory response was monitored by measurement of serum C-reactive protein and interleukin 6, and septic morbidity was recorded prospectively. RESULTS: Four patients were excluded owing to protocol violation, leaving 24 patients in group 1, 22 in group 2, 20 in group 3 and 22 in group 4 for analysis. There was a significant decrease in Enterobacteriaceae in group 3 compared with the other groups. Group 3 had a significantly lower incidence of translocation after bowel mobilization ($P < 0.001$). There was no significant difference between the groups in intestinal permeability, inflammatory response or septic morbidity. CONCLUSION: The combination of MBP, neomycin and synbiotics reduces the prevalence of faecal Enterobacteriaceae and bacterial translocation; however, this was not associated with a reduction in inflammatory response or septic morbidity in this study. Larger trials are needed before a change in practice can be recommended.

The information contained herein is to the best of our knowledge true and correct and presented in good faith. It may be subject to change without further notice. To the best of our knowledge this product does not infringe Intellectual Property Rights of any third party. This information is offered solely for your consideration and verification. Copyright© Chr. Hansen A/S. All rights reserved.

Research field: Immune Health
Research subfield: Cancer
Study type: Human study
Probiotic strain: B. animalis subsp. lactis BB-12 and L. rhamnosus LGG
Dosage CFU/day: 10 billion LGG , 10 billion BB-12, 10 g inulin enriched with oligofructose
Product formulation: NA
Reference number: 0538

Roller, et al. Consumption of prebiotic inulin enriched with oligofructose in combination with the probiotics Lactobacillus rhamnosus and Bifidobacterium lactis has minor effects on selected immune parameters in polypectomised and colon cancer patients. Br.J.Nutr. 2007;97:676-684

Abstract: Probiotics (PRO) modulate immunity in humans, while the effect of prebiotics (PRE) and synbiotics (SYN) on the human immune system are not well studied yet. The objective of this study was to investigate whether daily intake of a SYN modulates immune functions. In a randomised double-blind, placebo-controlled trial, thirty-four colon cancer patients who had undergone 'curative resection' and forty polypectomised patients participated. Subjects of the SYN group daily received encapsulated bacteria (1 x 10(10) colony-forming units of Lactobacillus rhamnosus GG (LGG) and 1 x 10(10) colony-forming units of Bifidobacterium lactis Bb12 (Bb12)) and 10 g of inulin enriched with oligofructose. Controls received encapsulated maltodextrin and 10 g of maltodextrin. Prior to intervention (T1), and 6 (T2) and 12 weeks after the start of the intervention (T3), phagocytic and respiratory burst activity of neutrophils and monocytes, lytic activity of natural killer cells and production of interleukin (IL)-2, IL-10 and IL-12, as well as tumour necrosis factor-alpha and interferon-gamma (IFN-gamma) by activated peripheral blood mononuclear cells (PBMC) were measured. In faeces, the concentrations of transforming growth factor-beta1 and prostaglandin E2 were measured. IL-2 secretion by activated PBMC from the polyp group increased significantly between T1 or T2 and T3 (P < 0.05). In the cancer group, SYN treatment resulted in an increased capacity of PBMC to produce IFN-gamma at T3 (P < 0.05). Other immunity-related parameters were not affected by SYN treatment, neither in the cancer nor in the polyp group. In conclusion, supplementation with this SYN has minor stimulatory effects on the systemic immune system of the two study groups. Further studies in humans should aim to focus on the gut-associated immune system.

The information contained herein is to the best of our knowledge true and correct and presented in good faith. It may be subject to change without further notice. To the best of our knowledge this product does not infringe Intellectual Property Rights of any third party. This information is offered solely for your consideration and verification. Copyright© Chr. Hansen A/S. All rights reserved.

CHR HANSEN

Research field: Immune Health
Research subfield: Immune stimulation
Study type: Human study
Probiotic strain: B. animalis subsp. lactis BB-12 and L. paracasei subsp. paracasei L. casei 431
Dosage CFU/day: 0.1 billion, 1 billion, 10 billion, 100 billion
Product formulation: Capsules
Reference number: 0501

Christensen, et al. Immunomodulating potential of supplementation with probiotics: a dose-response study in healthy young adults. FEMS Immunol.Med.Microbiol. 2006;47:380-390

Abstract: Certain probiotic microorganisms have been found beneficial in the treatment of immune-related diseases and may also affect immune function in healthy people. Intervention studies of probiotics in healthy humans are urgently required. Here, the immunomodulating potential of Bifidobacterium animalis ssp. lactis (BB-12) and Lactobacillus paracasei ssp. paracasei (CRL-431) was studied in a double-blind placebo-controlled parallel dose-response trial (n=71) based on five randomly assigned groups of young healthy adults supplemented for 3 weeks with 0, 10(8), 10(9), 10(10) and 10(11) CFU day(-1), respectively, of a mixture of BB-12 and CRL-431. No statistically significant dose-dependent effect was found for phagocytic activity in blood leukocytes, fecal immunoglobulin A (IgA) concentrations or production of interferon-gamma and interleukin-10 in blood cells. When evaluating data according to the amount of viable BB-12 recovered from faeces, the interferon-gamma production in blood cells was significantly reduced. In conclusion, no solid effect on the immune function of young healthy adults supplemented with even high doses of B. animalis ssp. lactis BB-12 and L. paracasei ssp. paracasei CRL-431 was demonstrated in this study.

The information contained herein is to the best of our knowledge true and correct and presented in good faith. It may be subject to change without further notice. To the best of our knowledge this product does not infringe Intellectual Property Rights of any third party. This information is offered solely for your consideration and verification. Copyright© Chr. Hansen A/S. All rights reserved.

CHR HANSEN

Research field: Gastrointestinal Health
Research subfield: Recovery
Study type: Human study
Probiotic strain: B. animalis subsp. lactis BB-12 and L. paracasei subsp. paracasei L. casei 431
Dosage CFU/day: 0.1 billion, 1 billion, 10 billion, 100 billion
Product formulation: Capsules
Reference number: 0500

Larsen, et al. Dose-response study of probiotic bacteria Bifidobacterium animalis subsp lactis BB-12 and Lactobacillus paracasei subsp paracasei CRL-341 in healthy young adults. Eur.J.Clin.Nutr. 2006;60(11):1284-1293

Abstract: OBJECTIVE: This study was performed to investigate the dose-response effects of supplementation with Bifidobacterium animalis subsp lactis (BB-12) and Lactobacillus paracasei subsp paracasei (CRL-431) on blood lipids, recovery from feces and bowel habits. Changes of the fecal microflora was analyzed in the 10(10) CFU/day probiotic and placebo group. DESIGN: The study was designed as a randomized, placebo-controlled, double-blinded, parallel dose-response study. SUBJECTS: Healthy young adults (18-40 years) were recruited by advertising in local newspapers. Of the 75 persons enrolled, 71 (46 women, 25 men, mean age 25.6 years (range 18-40 years)) completed the study. INTERVENTION: The volunteers were randomly assigned into five groups receiving either placebo or a mixture of the two probiotics in the concentration of 10(8), 10(9), 10(10) or 10(11) CFU/day in 2 weeks run-in period, 3 weeks intervention and 2 weeks wash-out. Diary reporting bowel habits and well being (abdominal bloating, flatulence and headache) was kept for all 7 weeks and blood lipids, fecal recovery of BB-12 and CRL-431, as well as fecal microflora was tested before, immediately and 2 weeks after intervention. RESULTS: The fecal recovery of BB-12 increased significantly ($P < 0.001$) with increasing dose. In the group receiving 10(11) CFU/day BB-12 was recovered from 13 out of 15 volunteers. CRL-431 was not recovered in any of the fecal samples. Supplementation with probiotics did not change the fecal bacterial composition. A significant linear increase in fecal consistency (looser stool) with increasing probiotic dose ($P = 0.018$) was observed. No overall dose-response effect was found on the blood lipids. High doses of probiotics were well tolerated. CONCLUSION: A dose-related recovery of BB-12 from feces was observed.

Chr. Hansen A/S -10-12 Bøge Allé – DK-2970 Hørsholm, Denmark - Phone: +45 45 74 74 74 - Fax: +45 45 74 88 88 - www.chr-hansen.com

The information contained herein is to the best of our knowledge true and correct and presented in good faith. It may be subject to change without further notice. To the best of our knowledge this product does not infringe Intellectual Property Rights of any third party. This information is offered solely for your consideration and verification. Copyright© Chr. Hansen A/S. All rights reserved.

CHR HANSEN

Research field: Gastrointestinal Health
Research subfield: Recovery
Study type: Human study
Probiotic strain: B. animalis subsp. lactis BB-12 and L. paracasei subsp. paracasei F19
Dosage CFU/day: 100 billion BB-12 and 20 billion F19
Product formulation: Fermented milk
Reference number: 0513

Matto, et al. Intestinal survival and persistence of probiotic Lactobacillus and Bifidobacterium strains administered in triple-strain yoghurt. Int.Dairy J. 2006;16(10):1174-1180

Abstract: The aim of the present study was to investigate the intestinal survival and persistence of probiotic strains Lactobacillus F19, Lactobacillus acidophilus NCFB 1748, and Bifidobacterium animalis subsp. lactis Bb-12 consumed in a yoghurt (ABC product), and also their effect on the intestinal microbiota. Based on the results of culture studies and strain-level analysis by randomly amplified polymorphic DNA (RAPD) fingerprinting Lactobacillus F19 and B. animalis subsp. lactis Bb-12 survived well through the human gastrointestinal tract; they were detected in reasonable numbers in the faeces of 100% and 79% of the study subjects, respectively. Ingestion of the probiotic yoghurt increased transiently the numbers of bifidobacteria and lactobacilli. For lactobacilli the increase was due to the detection of the ingested probiotic strains in faeces, while in bifidobacteria the increase was likely caused by the increase of indigenous bifidobacteria since the ingested Bifidobacterium strain did not comprise the predominant part of bifidobacterial population during the intervention. Probiotic strains were infrequently detected in mucosal biopsy samples. The present study indicates that developing probiotic food products with multiple probiotic strains is feasible.

Chr. Hansen A/S -10-12 Bøge Allé – DK-2970 Hørsholm, Denmark - Phone: +45 45 74 74 74 - Fax: +45 45 74 88 88 - www.chr-hansen.com

The information contained herein is to the best of our knowledge true and correct and presented in good faith. It may be subject to change without further notice. To the best of our knowledge this product does not infringe Intellectual Property Rights of any third party. This information is offered solely for your consideration and verification. Copyright© Chr. Hansen A/S. All rights reserved.

CHR HANSEN

Research field: Gastrointestinal Health
Research subfield: Microbiota
Study type: Human study
Probiotic strain: B. animalis subsp. lactis BB-12
Dosage CFU/day: 1.6 billion BB-12 day 1-3 and 4.8 billion BB-12 from day 4 onwards.
Product formulation: Milk powder
Reference number: 0511

Mohan, et al. Effects of Bifidobacterium lactis BB12 supplementation on intestinal microbiota of preterm infants: a double-blind placebo controlled randomized study. J.Clin.Microbiol. 2006;44(11):4025-4031

Abstract: The gastrointestinal microbiota of preterm infants in a neonatal intensive care unit differs from that of term infants. In particular, the colonization of preterm infants by bifidobacteria is delayed. A double-blind, placebo-controlled, randomized clinical study was performed on 69 preterm infants to investigate the role of Bifidobacterium lactis Bb12 supplementation in modifying the gut microbiota. Both culture-dependent and culture-independent approaches were used to study the gut microbiota. Bifidobacterial numbers, determined by fluorescence in situ hybridization, were significantly higher in the probiotic than in the placebo group (log(10) values per g of fecal wet weight: probiotic, 8.18 + 0.54 [standard error of the mean]; placebo, 4.82 + 0.51; $P < 0.001$). A similar trend for bifidobacterial numbers was also obtained with the culture-dependent method. The infants supplemented with Bb12 also had lower viable counts of Enterobacteriaceae (log(10) values of CFU per g of fecal wet weight: probiotic, 7.80 + 0.34; placebo, 9.03 + 0.35; $P = 0.015$) and Clostridium spp. (probiotic, 4.89 + 0.30; placebo, 5.99 + 0.32; $P = 0.014$) than the infants in the placebo group. Supplementation of B. lactis Bb12 did not reduce the colonization by antibiotic-resistant organisms in the study population. However, the probiotic supplementation increased the cell counts of bifidobacteria and reduced the cell counts of enterobacteria and clostridia.

The information contained herein is to the best of our knowledge true and correct and presented in good faith. It may be subject to change without further notice. To the best of our knowledge this product does not infringe Intellectual Property Rights of any third party. This information is offered solely for your consideration and verification. Copyright© Chr. Hansen A/S. All rights reserved.

CHR HANSEN

Research field: Gastrointestinal Health
Research subfield: Irregular bowel movements
Study type: Human study
Probiotic strain: B. animalis subsp. lactis BB-12
Dosage CFU/day: 0.9 billion
Product formulation: Fermented milk
Reference number: 0654

Murakami, et al. Safety and effect of yoghurt containing Bifidobacterium lactis Bb-12 on improvement of defecation and fecal microflora in healthy volunteers. J Nutr Food 2006:12-12

Abstract: DESIGN: In an 7-week double-blind crossover study, 36 subjects (mean age 28 y) were randomised to receiving yoghurt with BB-12 or placebo. Defecation condition and fecal properties were evaluated and fecal samples were examined for microflora. RESULTS: Human Clinical trial. BB-12 was safe. The defecation frequency during the BB-12 intake period was increased compared to the no-intake period for group who tended to be constipated (P<0.05). Comfort after defecation improved significantly after intake of placebo yoghurt (P<0.01). During the BB-12 period, the Bifidobacerium count increased (P<0.05), and the detection rate for Clostridium perfringens was lower (P<0.05) than during the placebo period.

The information contained herein is to the best of our knowledge true and correct and presented in good faith. It may be subject to change without further notice. To the best of our knowledge this product does not infringe Intellectual Property Rights of any third party. This information is offered solely for your consideration and verification. Copyright© Chr. Hansen A/S. All rights reserved.

CHR HANSEN

Research field: Immune Health
Research subfield: Immune stimulation
Study type: Human study
Probiotic strain: B. animalis subsp. lactis BB-12 and L. rhamnosus LGG
Dosage CFU/day: 10 billion BB-12 and 10 billion LGG
Product formulation: Milk powder
Reference number: 0509

Rautava, et al. Specific probiotics in enhancing maturation of IgA responses in formula-fed infants. Pediatr.Res. 2006;60:221-224

Abstract: The first months of life represent a critical period for the maturation of the infant's immune system and, thus, a window of opportunity for measures to reduce the risk of disease. We hypothesized that specific probiotics might promote mucosal immunologic maturation in formula-fed infants. The numbers of cow's milk-specific and total IgA-secreting cells were measured at 3, 7, and 12 mo of age in a double-blind placebo-controlled study of 72 infants with early artificial feeding. The infants consumed infant formula supplemented with specific probiotics (Lactobacillus GG and Bifidobacterium lactis Bb-12) or placebo during the first year of life. Further analyses of the serum concentrations of the IgA-inducing cytokine TGF-beta2 and the soluble innate microbial receptor sCD14 were conducted. The numbers of cow's milk-specific IgA secreting cells were significantly higher in infants receiving probiotics compared with those receiving placebo (p = 0.045, ANOVA for repeated measures). At 12 mo of age, the serum concentrations of sCD14 were 1479 pg/mL [95% confidence interval (CI) 1373-1592] in infants receiving probiotics and 1291 pg/mL (95% CI 1152-1445) in infants receiving placebo (p = 0.046). Administration of the probiotics Lactobacillus GG and Bifidobacterium lactis Bb-12 at the time of introduction of cow's milk in the infant's diet results in cow's milk-specific IgA antibody responsiveness that may be the result of increased production of sCD14.

Chr. Hansen A/S -10-12 Bøge Allé – DK-2970 Hørsholm, Denmark - Phone: +45 45 74 74 74 - Fax: +45 45 74 88 88 - www.chr-hansen.com

The information contained herein is to the best of our knowledge true and correct and presented in good faith. It may be subject to change without further notice. To the best of our knowledge this product does not infringe Intellectual Property Rights of any third party. This information is offered solely for your consideration and verification. Copyright© Chr. Hansen A/S. All rights reserved.

CHR HANSEN

Research field: Gastrointestinal Health
Research subfield: Diarrhea
Study type: Human study
Probiotic strain: B. animalis subsp. lactis BB-12 and L. acidophilus LA-5
Dosage CFU/day: > 400 billion of each bacteria
Product formulation: Fermented milk
Reference number: 0508

Sheu, et al. Pretreatment with Lactobacillus- and Bifidobacterium-containing yogurt can improve the efficacy of quadruple therapy in eradicating residual Helicobacter pylori infection after failed triple therapy. Am.J.Clin.Nutr. 2006;83:864-869

Abstract: Background: Lactobacillus- and Bifidobacterium-containing yogurt (AB-yogurt) can suppress Helicobacter pylori. Improvement of the eradication rate by quadruple therapy of residual H. pylori after failed triple therapy is needed. /// Objective: We tested whether prior treatment with AB-yogurt improved the efficacy of quadruple therapy in eradicating residual H. pylori after failed triple therapy. /// Design: One hundred thirty-eight patients in whom triple therapy failed were enrolled for a culture study of H. pylori to assess antimicrobial resistance. These patients were then randomly assigned in equal numbers to either a yogurt-plus-quadruple therapy group or a quadruple therapy-only group. The patients received 1 wk of quadruple therapy with or without a 4-wk pretreatment with AB-yogurt (400 mL/d). In the yogurt-plus-quadruple group, excessive ÃŽÂ´13CO2/mL values of the 13C-urea breath test were collected before and every 2 wk during the 4-wk ingestion of yogurt. For both groups, a 13C-urea breath test was conducted Ã¢â€°Â¥6 wk after the quadruple therapy to assess the outcome of residual H. pylori eradication. /// Results: For the patients in the yogurt-plus-quadruple therapy group infected with either antibiotic-sensitive or -resistant H. pylori, the excessive ÃŽÂ´13CO2/mL values of the 13C-urea breath test were significantly decreased after the 4-wk ingestion of AB-yogurt (P < 0.0001). The yogurt-plus-quadruple therapy group had a higher H. pylori eradication rate than did the quadruple therapy-only group (intention-to-treat analysis: 85% compared with 71.1%, P < 0.05; per-protocol analysis: 90.8% compared with 76.6%, P < 0.05). /// Conclusion: A 4-wk pretreatment with AB-yogurt can decrease H. pylori loads despite antimicrobial resistance, thus improving the efficacy of quadruple therapy in eradicating residual H. pylori.

The information contained herein is to the best of our knowledge true and correct and presented in good faith. It may be subject to change without further notice. To the best of our knowledge this product does not infringe Intellectual Property Rights of any third party. This information is offered solely for your consideration and verification. Copyright© Chr. Hansen A/S. All rights reserved.

CHR. HANSEN

Research field: Other
Research subfield: Safety
Study type: Human study
Probiotic strain: B. animalis subsp. lactis BB-12
Dosage CFU/day: 0.8 billion
Product formulation: Milk powder
Reference number: 0512

Weizman, Alsheikh. Safety and tolerance of a probiotic formula in early infancy comparing two probiotic agents: a pilot study. J.Am.Coll.Nutr. 2006;25:415-419

Abstract: OBJECTIVE: To compare the safety and tolerance of two formulas, supplemented with different probiotic agents, in early infancy. DESIGN: Prospective randomized placebo-controlled trial. SETTING: Clinics of a University Medical Center. SUBJECTS: Full-term healthy infants aged less than 4 months. INTERVENTION: Infants were randomly assigned for 4 weeks to a standard milk-based formula supplemented with either Bifidobacterium lactis (BB-12), Lactobacillus reuteri (ATCC 55730) or a probiotics-free formula. MEASURES OF OUTCOME: Growth parameters, daily characteristics of feeding, stooling and behavior, and side effects. RESULTS: Fifty-nine infants, aged 3-65 days, were included. Subjects in all three groups were similar at entry in terms of gestational age, birth weight, sex, growth parameters and breast feeding rate prior to the study. The supplemented formulas were well accepted and did not reveal any adverse effects. A comparison of growth parameters, and variables of feeding, stooling and crying and irritability did not reveal any significant differences between groups. CONCLUSIONS: The use of formula supplemented with either Lactobacillus reuteri or Bifidobacterium lactis in early infancy, was safe, well tolerated and did not adversely affect growth, stooling habits or infant behavior.

The information contained herein is to the best of our knowledge true and correct and presented in good faith. It may be subject to change without further notice. To the best of our knowledge this product does not infringe Intellectual Property Rights of any third party. This information is offered solely for your consideration and verification. Copyright© Chr. Hansen A/S. All rights reserved.

CHR HANSEN

Research field: Gastrointestinal Health
Research subfield: Diarrhea
Study type: Human study
Probiotic strain: B. animalis subsp. lactis BB-12 and L. acidophilus LA-5
Dosage CFU/day: 40 billion
Product formulation: Capsules
Reference number: 0499

Wildt, et al. Probiotic treatment of collagenous colitis: a randomized, double-blind, placebo-controlled trial with Lactobacillus acidophilus and Bifidobacterium animalis subsp. Lactis. Inflamm.Bowel Dis. 2006;12:395-401

Abstract: BACKGROUND: Probiotic treatment may be effective in diseases involving gut microflora and intestinal inflammation. In collagenous colitis (CC), a potential pathogenic role of the gut microflora has been proposed. The effect of probiotic treatment in CC is unknown. Our aim was to investigate the clinical effect of treatment with Lactobacillus acidophilus LA-5 and Bifidobacterium animalis subsp. lactis BB-12 (AB-Cap-10) in patients with CC. MATERIALS AND METHODS: Patients with CC and diarrhea were in a double-blind placebo-controlled study randomized (2:1) to AB-Cap-10 or placebo for 12 weeks. The primary end point was reduction in bowel frequency per week of >or=50%. Secondary end points were changes in bowel frequencies, stool consistency, stool weight, histopathology, and abdominal bloating and pain. RESULTS: Twenty-nine patients were randomized: 21 to probiotics and 8 to placebo. Reduction in bowel frequency per week of >or=50% occurred in 6 of 21 (29%) and in 1 of 8 (13%) patients receiving probiotic and placebo, respectively (P = 0.635). No differences between treatments were observed regarding the secondary end points. Post hoc analysis showed a median reduction in bowel frequency per week from 32 (range 18-84) to 23 (range 11-56; P < 0.005), a reduction in number of days with liquid stools per week from 6 days (range 0-7 days) to 1 day (range 0-7 days; P < 0.005), and an increase in number of days with solid stools per week (P < 0.05) in the AB-Cap-10 group. CONCLUSIONS: AB-Cap-10 had no significant effect on the chosen end points. Post hoc analysis demonstrated amelioration of clinical symptoms in the AB-Cap-10 group, indicating that probiotic treatment may potentially influence the disease course of CC.

The information contained herein is to the best of our knowledge true and correct and presented in good faith. It may be subject to change without further notice. To the best of our knowledge this product does not infringe Intellectual Property Rights of any third party. This information is offered solely for your consideration and verification. Copyright© Chr. Hansen A/S. All rights reserved.

Research field: Immune Health
Research subfield: Necrotizing enterocolitis
Study type: Human study
Probiotic strain: BB-12 and BB-02 and CH-3
Dosage CFU/day: 1 billion
Product formulation: NA
Reference number: 0495

Bin-Nun, et al. Oral probiotics prevent necrotizing enterocolitis in very low birth weight neonates. J.Pediatr. 2005;147:192-196

Abstract: OBJECTIVE: To test the hypothesis that normalizing the intestinal flora by administration of prophylactic probiotics would provide a natural defense, thereby reducing both the incidence and severity of necrotizing enterocolitis (NEC) in preterm neonates. STUDY DESIGN: Neonates < or =1500 g birth weight were randomized to either receive a daily feeding supplementation with a probiotic mixture (Bifidobacteria infantis, Streptococcus thermophilus, and Bifidobacteria bifidus; Solgar, Israel) of 10(9) colony forming units (CFU)/day or to not receive feed supplements. NEC was graded according to Bell's criteria. RESULTS: For 72 study and 73 control infants, respectively, birth weight (1152 +/- 262 g vs 1111 +/- 278 g), gestational age (30 +/- 3 weeks vs 29 +/- 4 weeks), and time to reach full feeds (14.6 +/- 8.7 days vs 17.5 +/- 13.6 days) were not different. The incidence of NEC was reduced in the study group (4% vs 16.4%; P=.03). NEC was less severe in the probiotic-supplemented infants (Bell's criteria 2.3 +/- 0.5 vs 1.3 +/- 0.5; P=.005). Three of 15 babies who developed NEC died, and all NEC-related deaths occurred in control infants. CONCLUSION: Probiotic supplementation reduced both the incidence and severity of NEC in our premature neonatal population.

The information contained herein is to the best of our knowledge true and correct and presented in good faith. It may be subject to change without further notice. To the best of our knowledge this product does not infringe Intellectual Property Rights of any third party. This information is offered solely for your consideration and verification. Copyright© Chr. Hansen A/S. All rights reserved.

CHR. HANSEN

Research field: Immune Health
Research subfield: Cancer
Study type: Human study
Probiotic strain: B. animalis subsp. lactis BB-12 and L. rhamnosus LGG
Dosage CFU/day: NA
Product formulation: NA
Reference number: 0497

Collins, et al. The influence of synbiotic consumption on cancer risk biomarkers in previously resected colon cancer subjects. Journal of Biotechnology 2005

Abstract: 37 colon cancer subjects who had undergone curative resection were supplemented with BB-12, L.GG and raftilose for 12 weeks. Fecal and blood samples were obtained before, midway through (6 weeks) and following intervention (12 weeks). Rectal biopsies were obtained at T1 and T3. Fecal flora was determined using plating techniques. Genotoxic and cytotoxic potential of Fecal Water was determined. Cytokine production was estimated in vitro. Flow cytometry was used to determine NK cell cytotoxic activity as well as the phagocytic and respiratory burst activity of monocytes. In the synbiotic group fecal numbers of bifidobacteria increased significantly ($p>0.001$), lactobacilli increased (ns $p=0.0674$) while coliforms decreased ($p<0.05$). In the placebo group bifidobacteria decreased ($p<0.001$), the other bacterial groups were unaffected. Genotoxic damage was increased in placebo biopsies but unchanged in the probiotic group. The geno- and cytotoxic potential of FW was unaltered. IFN-gamma production was significantly increased in synbiotic group but IL-2, IL-10, IL-12 and TNF-alpha production was unaffected. In conclusion synbiotic consumption of BB-12, L.GG and raftilose beneficially altered the composition of the gut flora and protected against genotoxic damage in vivo suggesting a protective effect against colon carcinogenesis.

The information contained herein is to the best of our knowledge true and correct and presented in good faith. It may be subject to change without further notice. To the best of our knowledge this product does not infringe Intellectual Property Rights of any third party. This information is offered solely for your consideration and verification. Copyright© Chr. Hansen A/S. All rights reserved.

Research field: Gastrointestinal Health
Research subfield: Various
Study type: Human study
Probiotic strain: B. animalis subsp. lactis BB-12 and L. acidophilus LA-5 and L. bulgaricus LBY-27 and S. thermophilus STY-31
Dosage CFU/day: 12 billion
Product formulation: Capsules
Reference number: 0490

Gatt, et al. Randomized clinical trial of multimodal optimization of surgical care in patients undergoing major colonic resection. Br.J.Surg. 2005;92(11):1354-1362

Abstract: BACKGROUND: The aim of this trial was to compare multimodal optimization with conventional perioperative management in a consecutive series of patients undergoing a wide range of colorectal procedures. METHODS: Thirty-nine patients undergoing major elective colonic resection were recruited prospectively. Patients were randomized to receive a ten-point multimodal optimization package or conventional perioperative care. All patients were administered epidural analgesia and opiates were avoided. Outcome measures recorded related to length of hospital stay, physical and mental function, and gut function. RESULTS: Optimization was associated with a significantly shorter median (interquartile range) hospital stay compared with conventional care (5 (4-9) versus 7.5 (6-10) days; P = 0.027). Duration of catheterization (P = 0.022) and duration of intravenous infusion (P = 0.007) were also less. Optimization was associated with a quicker recovery of gut function (P = 0.042). Grip strength was maintained in the postoperative period in the optimized group (P = 0.241) but not in the control group (P = 0.049). There were no differences in morbidity or mortality between the groups. CONCLUSION: Optimization is safe and results in a significant reduction in postoperative stay along with other improved endpoints. This cannot be directly attributed to improvement in any single outcome measure or to the use of epidural analgesia. Improvements are more likely to be multifactorial and may relate to an earlier return of gut function.

The information contained herein is to the best of our knowledge true and correct and presented in good faith. It may be subject to change without further notice. To the best of our knowledge this product does not infringe Intellectual Property Rights of any third party. This information is offered solely for your consideration and verification. Copyright© Chr. Hansen A/S. All rights reserved.

CHR HANSEN

Research field: Gastrointestinal Health
Research subfield: Microbiota
Study type: Human study
Probiotic strain: B. animalis subsp. lactis BB-12
Dosage CFU/day: 25 billion
Product formulation: -
Reference number: 0660

Jernberg, et al. Monitoring of antibiotic-induced alterations in the human intestinal microflora and detection of probiotic strains by use of terminal restriction fragment length polymorphism. Appl.Environ.Microbiol. 2005;71:501-506

Abstract: Terminal restriction fragment length polymorphism (T-RFLP) was investigated as a tool for monitoring the human intestinal microflora during antibiotic treatment and during ingestion of a probiotic product. Fecal samples from eight healthy volunteers were taken before, during, and after administration of clindamycin. During treatment, four subjects were given a probiotic, and four subjects were given a placebo. Changes in the microbial intestinal community composition and relative abundance of specific microbial populations in each subject were monitored by using viable counts and T-RFLP fingerprints. T-RFLP was also used to monitor specific bacterial populations that were either positively or negatively affected by clindamycin. Some dominant bacterial groups, such as Eubacterium spp., were easily monitored by T-RFLP, while they were hard to recover by cultivation. Furthermore, the two probiotic Lactobacillus strains were easily tracked by T-RFLP and were shown to be the dominant Lactobacillus community members in the intestinal microflora of subjects who received the probiotic.

The information contained herein is to the best of our knowledge true and correct and presented in good faith. It may be subject to change without further notice. To the best of our knowledge this product does not infringe Intellectual Property Rights of any third party. This information is offered solely for your consideration and verification. Copyright© Chr. Hansen A/S. All rights reserved.

CHR HANSEN

Research field: Gastrointestinal Health
Research subfield: Other
Study type: Human study
Probiotic strain: B. animalis subsp. lactis BB-12 and L. acidophilus LA-5
Dosage CFU/day: 50 billion
Product formulation: Fermented milk
Reference number: 0473

Laake, et al. Outcome of four weeks' intervention with probiotics on symptoms and endoscopic appearance after surgical reconstruction with a J-configurated ileal-pouch-anal-anastomosis in ulcerative colitis. Scand.J.Gastroenterol. 2005;40:43-51

Abstract: OBJECTIVE: Pouchitis is a common and troublesome condition in patients operated on with ileal-pouch-anal-anastomosis (IPAA). A disturbed microecology in the pouch has been suggested as one possible explanation. In a previous double-blind, randomized, controlled study we demonstrated clinical improvement of symptoms in patients with ulcerative colitis (UC) operated on with IPAA, during intervention with live probiotic microbes Lactobacilli and Bifidobacteriae. The aim of the present study was to confirm our previous results in a much larger material, including clinical symptoms, faecal flora and endoscopic evaluation, and to compare the results in UC/IPAA patients with those of patients with familial adenomatous polyposis (FAP) with IPAA and UC patients with ileorectal anastomosis (IRA). MATERIAL AND METHODS: Five hundred millilitres of a fermented milk product (Cultura) containing live lactobacilli (La-5) and bifidobacteriae (Bb-12) was given daily for 4 weeks to 51 UC patients and 10 patients with FAP, operated on with IPAA, and six UC patients operated on for IRA. Stool samples were cultured for examination of lactobacilli, bifidobacteriae, fungi and pH before, during and after intervention. Before, during and after intervention, endoscopic evaluation was performed. Categorized symptomatology was examined prospectively using diary cards in addition to an interview, before and on the last day of intervention. RESULTS: The number of lactobacilli and bifidobacteriae increased significantly during intervention in the UC patients operated on with IPAA and remained significantly increased one week after intervention. Involuntary defecation, leakage, abdominal cramps and the need for napkins (category I), faecal number and consistency (category II) and mucus and urge to evacuate stools (category III) were significantly decreased during intervention in the UC/IPAA group. In the FAP group there was a significant decrease in faecal leakage, abdominal cramps and use of napkins (category I) during intervention. The median endoscopic score of inflammation was significantly decreased during intervention in the UC/IPAA patients. Blood tests, faecal fungi and faecal pH did not change significantly during intervention. CONCLUSIONS: Results of this extended study, showing an effect of probiotics on symptoms and endoscopic inflammation in UC patients operated on with IPAA confirm our previously reported effect of probiotics on clinical symptoms and endoscopic score in a smaller, double-blind, randomized, controlled study. The significantly higher response to probiotics in families with increased risk of IBD will have to be repeated in future studies.

The information contained herein is to the best of our knowledge true and correct and presented in good faith. It may be subject to change without further notice. To the best of our knowledge this product does not infringe Intellectual Property Rights of any third party. This information is offered solely for your consideration and verification. Copyright© Chr. Hansen A/S. All rights reserved.

CHR HANSEN

Research field: Gastrointestinal Health
Research subfield: Microbiota
Study type: Human study
Probiotic strain: B. animalis subsp. lactis BB-12
Dosage CFU/day: NA
Product formulation: Other
Reference number: 0469

Rinne, et al. Similar bifidogenic effects of prebiotic-supplemented partially hydrolyzed infant formula and breastfeeding on infant gut microbiota. FEMS Immunol.Med.Microbiol. 2005;43:59-65

Abstract: The aim of the study was to assess the quantitative and qualitative differences of the gut microbiota in infants. We evaluated gut microbiota at the age of 6 months in 32 infants who were either exclusively breast-fed, formula-fed, nursed by a formula supplemented with prebiotics (a mixture of fructo- and galacto-oligosaccharides) or breast-fed by mothers who had been given probiotics. The Bifidobacterium, Bacteroides, Clostridium and Lactobacillus/Enterococcus microbiota were assessed by the fluorescence in situ hybridization, and Bifidobacterium species were further characterized by PCR. Total number of bifidobacteria was lower among the formula-fed group than in other groups (P=0.044). Total amounts of the other bacteria were comparable between the groups. The specific Bifidobacterium microbiota composition of the breast-fed infants was achieved in infants receiving prebiotic supplemented formula. This would suggest that early gut Bifidobacterium microbiota can be modified by special diets up to the age of 6 months.

The information contained herein is to the best of our knowledge true and correct and presented in good faith. It may be subject to change without further notice. To the best of our knowledge this product does not infringe Intellectual Property Rights of any third party. This information is offered solely for your consideration and verification. Copyright© Chr. Hansen A/S. All rights reserved.

CHR HANSEN

Research field: Gastrointestinal Health
Research subfield: Irregular bowel movements
Study type: Human study
Probiotic strain: B. animalis subsp. lactis BB-12
Dosage CFU/day: Test 1: 1 billion, test 2: 3 billion
Product formulation: Fermented milk
Reference number: 0658

Uchida, et al. Effect of fermented milk containing Bifidobacterium lactis Bb-12 on stool frequency, defecation, fecal microbiota and safety of excessive ingestion in healthy female students -2nd report. J Nutr Food 2005:39-51

Abstract: DESIGN: Test 1: With the objective to elucidate the effect of fermented milk containing BB-12 on defecation and intestinal flora, 41 healthy female volunteers (mean age 21 y) with constipation tendency (== 5 times / week) participated in an 8-week double blind placebo controlled crossover study with four 2-week periods; control period with no intake (1. and 3. period), and intake period (2. and 4. period) with intake of fermented milk with BB-12/placebo. Test 2: With the objective to examine the safety of excessive ingestion of fermented milk containing BB-12, 18 other healthy female volunteers (mean age 21 y) consumed 300 g of fermented milk with BB-12 in a 3-week study with a no intake period (1 wk), intake period (1 wk), and no intake period (1 wk). RESULTS: Test 1: Stool frequency increased in the BB-12 period compared with the placebo period for all subjects and for the constipation tendency group ($p<0.05$). Amount of defecation also increased during the BB-12 period ($p<0.05$). The color, odor and shape of feces were improved by BB-12. Bifidobacterium count on the total bacterial count was higher in the BB-12 period (34.5%) than in the placebo period (24.3%, $p<0.05$) and the no intake period (22.8%, $p<0.05$). Test 2: There were no difference in abdominal symptoms, such as abdominal pain, abdominal distension, gas, diarrhea, abdominal rumbling, and desire to defecate without defecation.

The information contained herein is to the best of our knowledge true and correct and presented in good faith. It may be subject to change without further notice. To the best of our knowledge this product does not infringe Intellectual Property Rights of any third party. This information is offered solely for your consideration and verification. Copyright© Chr. Hansen A/S. All rights reserved.

CHR HANSEN

Research field: Other
Research subfield: Gut barrier function
Study type: Human study
Probiotic strain: B. animalis subsp. lactis BB-12 and L. acidophilus LA-5 and L. bulgaricus LBY-27 and S. thermophilus STY-31
Dosage CFU/day: 12 billion
Product formulation: Capsules
Reference number: 0434

Anderson, et al. Randomised clinical trial of synbiotic therapy in elective surgical patients. Gut 2004;53:241-245

Abstract: BACKGROUND: It is possible to manipulate the composition of the gastrointestinal microflora by administration of pre- and probiotics. This may help to preserve gut barrier function and reduce the incidence of septic morbidity. AIMS: To assess the effects of a combination of pre- and probiotics (synbiotic) on bacterial translocation, gastric colonisation, systemic inflammation, and septic morbidity in elective surgical patients. PATIENTS: Patients were enrolled two weeks prior to elective abdominal surgery. Seventy two patients were randomised to the synbiotic group and 65 to the placebo group. Patients were well matched regarding age and sex distribution, diagnoses, and POSSUM scores. METHODS: Patients in the synbiotic group received a two week preoperative course of Lactobacillus acidophilus La5, Bifidobacterium lactis Bb-12, Streptococcus thermophilus, and Lactobacillus bulgaricus, together with the prebiotic oligofructose. Patients in the placebo group received placebo capsules and sucrose powder. At surgery, a nasogastric aspirate, mesenteric lymph node, and scrapings of the terminal ileum were harvested for microbiological analysis. Serum was collected preoperatively and on postoperative days 1 and 7 for measurement of C reactive protein, interleukin 6, and antiendotoxin antibodies. Septic morbidity and mortality were recorded. RESULTS: There were no significant differences between the synbiotic and control groups in bacterial translocation (12.1% v 10.7%; p = 0.808, chi(2)), gastric colonisation (41% v 44%; p = 0.719), systemic inflammation, or septic complications (32% v 31%; p = 0.882). CONCLUSIONS: In this study, synbiotics had no measurable effect on gut barrier function in elective surgical patients. Further studies investigating the place of pre- and probiotics in clinical practice are required.

The information contained herein is to the best of our knowledge true and correct and presented in good faith. It may be subject to change without further notice. To the best of our knowledge this product does not infringe Intellectual Property Rights of any third party. This information is offered solely for your consideration and verification. Copyright© Chr. Hansen A/S. All rights reserved.

CHR HANSEN

Research field: Gastrointestinal Health
Research subfield: Diarrhea
Study type: Human study
Probiotic strain: B. animalis subsp. lactis BB-12
Dosage CFU/day: > 100 million
Product formulation: Milk powder
Reference number: 0437

Chouraqui, et al. Acidified milk formula supplemented with bifidobacterium lactis: impact on infant diarrhea in residential care settings. J.Pediatr.Gastroenterol.Nutr. 2004;38:288-292

Abstract: OBJECTIVES: Probiotics may be useful in preventing acute infectious diarrhea. Bifidobacteria are particularly attractive as probiotics agent because they constitute the predominant colonic flora of breastfed infants and are thought to play a role in the decreased incidence of diarrhea in breastfed infants. METHODS: This was a multicenter, double-blind, controlled study to evaluate the efficacy of a milk formula supplemented with viable Bifidobacterium lactis strain Bb 12 (BbF) in the prevention of acute diarrhea in infants younger than 8 months living in residential nurseries or foster care centers. RESULTS: Ninety healthy children received either the BbF or a conventional formula (CF) daily. The mean duration of the stay in the residential center was similar (137 v 148 days). At enrollment, there were no differences between the two groups with respect to age (3.7 +/- 2.1 months), gender, anthropometric data, history of allergy or gastrointestinal disease, frequency of breast-feeding in the neonatal period or timing of introduction of solid food. Altogether, 28.3% of the BbF infants had diarrhea during the study compared with 38.7% of controls (NS). There was a statistically insignificant trend for shorter episodes of diarrhea in the BbF group (5.1 +/- 3.3 days v 7 +/- 5.5 days, NS). The number of days with diarrhea was 1.15 +/- 2.5 in the BbF group with a daily probability of diarrhea of 0.84 versus 2.3 +/- 4.5 days and 1.55, respectively, in the CF group (P = 0.0002 and 0.0014). Feeding infants with the BbF reduced their risk of getting diarrhea by a factor of 1.9 (range, 1.33-2.6). Analysis of the cumulative incidence of diarrheal episodes showed a trend that the first onset of diarrhea occurred later in the BbF group. CONCLUSION: These results provide some evidence that viable Bifidobacterium lactis strain Bb 12, added to an acidified infant formula, has some protective effect against acute diarrhea in healthy children.

Chr. Hansen A/S -10-12 Bøge Allé – DK-2970 Hørsholm, Denmark - Phone: +45 45 74 74 74 - Fax: +45 45 74 88 88 - www.chr-hansen.com

The information contained herein is to the best of our knowledge true and correct and presented in good faith. It may be subject to change without further notice. To the best of our knowledge this product does not infringe Intellectual Property Rights of any third party. This information is offered solely for your consideration and verification. Copyright© Chr. Hansen A/S. All rights reserved.

CHR HANSEN

Research field: Other
Research subfield: Various
Study type: Human study
Probiotic strain: B. animalis subsp. lactis BB-12 and L. acidophilus LA-5 and L. bulgaricus LBY-27 and S. thermophilus STY-31
Dosage CFU/day: 12 billion
Product formulation: Capsules
Reference number: 0449

Jain, et al. Influence of synbiotic containing Lactobacillus acidophilus La5, Bifidobacterium lactis Bb 12, Streptococcus thermophilus, Lactobacillus bulgaricus and oligofructose on gut barrier function and sepsis in critically ill patients: a randomised controlled trial. Clin.Nutr. 2004;23:467-475

Abstract: BACKGROUND & AIMS: Infective complications are a common cause of mortality and morbidity in critically ill patients. Many factors affect sepsis, one of which is gut barrier function. The aim of this study was to determine whether the oral administration of a synbiotic preparation could alter gut barrier function in critically ill patients and thus reduce sepsis. METHODS: A total of 90 patients admitted to an intensive care unit (ICU) were randomised to receive either synbiotic or placebo preparations (45 into each group). The synbiotic preparation consisted of Lactobacillus acidophilus La5, Bifidobacterium lactis Bb 12, Streptococcus thermophilus and Lactobacillus bulgaricus (probiotics) with oligofructose (prebiotic). Gut barrier function was assessed by measurement of intestinal permeability (lactulose/rhamnose test) and culture of nasogastric aspirate on days 1 and 8. All septic complications and mortality were recorded. RESULTS: There were no differences between the groups in terms of age, sex, APACHE II or POSSUM scores. After 1 week of therapy, patients in the synbiotic group had a significantly lower incidence of potentially pathogenic bacteria (43% versus 75%, P = 0.05) and multiple organisms (39% versus 75%, P = 0.01) in their nasogastric aspirates than controls. There were no significant differences between the groups in terms of intestinal permeability, septic complications or mortality. CONCLUSIONS: The administration of synbiotic in critically ill patients favourably altered the microbial composition of the upper gastrointestinal tract but had no effect on intestinal permeability and was not associated with measurable clinical benefit.

Chr. Hansen A/S -10-12 Bøge Allé – DK-2970 Hørsholm, Denmark - Phone: +45 45 74 74 74 - Fax: +45 45 74 88 88 - www.chr-hansen.com

The information contained herein is to the best of our knowledge true and correct and presented in good faith. It may be subject to change without further notice. To the best of our knowledge this product does not infringe Intellectual Property Rights of any third party. This information is offered solely for your consideration and verification. Copyright© Chr. Hansen A/S. All rights reserved.

CHR HANSEN

Research field: Gastrointestinal Health
Research subfield: Inflammatory bowel disease
Study type: Human study
Probiotic strain: B. animalis subsp. lactis BB-12 and L. acidophilus LA-5
Dosage CFU/day: Approx. 50 billion of each bacteria
Product formulation: Fermented milk
Reference number: 0466

Laake, et al. Assessment of mucosal inflammation and blood flow in response to four weeks' intervention with probiotics in patients operated with a J-configurated ileal-pouch-anal-anastomosis (IPAA). Scand.J.Gastroenterol. 2004;39(12):1228-1235

Abstract: BACKGROUND: Pouchitis is a common and troublesome condition in patients operated on with ileal-pouch-anal-anastomosis (IPAA). A disturbed mucosal perfusion in the pouch has been suggested as a possible cause. Laser Doppler flowmetry (LDF) has been used successfully to measure gastric and colonic mucosal perfusion in humans. In a previous study, we demonstrated a reduced mucosal perfusion in the distal part of the pouch, during probiotic intervention, examined by LDF measurement. The aim of the present study was to confirm our previous results in a much larger material, and to compare the results of LDF measurements and inflammatory activity in ulcerative colitis (UC) patients with those in familial adenomatous polyposis (FAP) patients. METHODS: Five hundred millilitres of a fermented milk product (Cultura), containing live lactobacilli (La-5) and bifidobacteria (Bb-12), was given daily for 4 weeks to 41 UC and 10 patients with FAP, operated on with IPAA. Mucosal perfusion was measured with LDF and the degree of inflammation was examined at predefined levels of the distal bowel by histology and faecal calprotectin measurements both before and after intervention. We also evaluated the applicability of a Pouchitis Disease Activity Index (PDAI). RESULTS: The LDF measurements were reproducible in the pelvic pouch at each of the predefined levels, but did not change during intervention. Mucosal perfusion was significantly reduced in the distal compared to the proximal part of the pouch in the UC group ($P < 0.05$). The perfusion levels were higher in the FAP patients compared to the UC patientsat all predefined levels ($P < 0.05$). Calprotectin levels and histological score did not change significantlyafter intervention in any of the groups. The calprotectin level was significantly lower in the FAP compared to the UC group both before and after intervention. The PDAI decreased in both groups from alevel considered diagnostic for pouchitis to a level considered as not active pouchitis. The decreasewas significant for the UC patients. CONCLUSIONS: The results did not demonstrate an effect of probiotics on histology, although a significant effect on the PDAI was achieved, which concurs with the previously reported effect on symptoms and endoscopic score. The significantly reduced blood flow in the UC group compared to the FAP group, operated on with the same procedure, and the significantly increased calprotectin levels in the UC group, are original findings. Both findings may be related to an increased risk for pouchitis among UC patients. The lack of effect of intervention on mucosal perfusion does not exclude a role for reduced circulation as a cause of pouchitis based on the reduced LDF measurements in the distal part of the pouch.

The information contained herein is to the best of our knowledge true and correct and presented in good faith. It may be subject to change without further notice. To the best of our knowledge this product does not infringe Intellectual Property Rights of any third party. This information is offered solely for your consideration and verification. Copyright© Chr. Hansen A/S. All rights reserved.

CHR HANSEN

Research field: Immune Health
Research subfield: Cancer
Study type: Human study
Probiotic strain: B. animalis subsp. lactis BB-12
Dosage CFU/day: 5.2 billion
Product formulation: Fermented milk
Reference number: 0455

Matsumoto, Benno. Consumption of Bifidobacterium lactis LKM512 yogurt reduces gut mutagenicity by increasing gut polyamine contents in healthy adult subjects. Mutat.Res. 2004;568:147-153

Abstract: The possible role of probiotic metabolites on human health effects of probiotics has received little research attention. In this study, we investigated the effects of consumption of Bifidobacterium lactis LKM512-containing yogurt (LKM512 yogurt) on fecal probiotic metabolites (polyamines, lactate, and acetate) and mutagenicity in seven healthy adults (one male and six females; average age: 30.5 years). Each volunteer was provided with 100g/day of LKM512 yogurt or placebo for 2 weeks. Fecal polyamines and mutagenicity were measured by HPLC and the umu-test, respectively. Consumption of LKM512 yogurt increased fecal spermidine levels, but not fecal lactate and acetate contents. The mutagenicity level significantly reduced to 79.2% (10-91.1%) and 47.9% (0-86.8%) following consumption of LKM512 yogurt (P=0.0293) and placebo (P=0.0314), respectively. LKM512 yogurt consumption significantly reduced the mutagenicity level compared with consumption of a placebo (P=0.0489). These results suggest that increased gut spermidine level by LKM512 yogurt was responsible for the reduction of mutagenicity in the gut of healthy adults. We suggest that spermidine produced by LKM512 yogurt consumption contributes to host health as a bioantimutagenic factor; to our knowledge, these substances have not been previously reported as antimutagens from probiotics or fermented milk.

The information contained herein is to the best of our knowledge true and correct and presented in good faith. It may be subject to change without further notice. To the best of our knowledge this product does not infringe Intellectual Property Rights of any third party. This information is offered solely for your consideration and verification. Copyright© Chr. Hansen A/S. All rights reserved.

Research field: Gastrointestinal Health
Research subfield: Irregular bowel movements
Study type: Human study
Probiotic strain: B. animalis subsp. lactis BB-12
Dosage CFU/day: Test 1: 3.2 billion or 6.0 billion, test 2: 18 billion
Product formulation: Fermented milk
Reference number: 0656

Nishida, et al. Improvement effect of Yoghurt Containing Bifidobacterium lactis strain BB-12 on Defecation and Fecal Microflora and its Safety to Healthy Human Adults. Milk Science 2004;53:133-140

Abstract: DESIGN: With the objective to elucidate the influence of quantity of yoghurt containing BB-12 on the improvement of defecation and fecal microflora, 2 tests were conducted; test 1) 15 healthy adults (mean age 40 y), received either 80g/d or 150g/d of BB-12 yoghurt for 14 days, and test 2) 10 healthy adults (mean age 40 y), received either 450g/d of BB-12 yoghurt for 14 days. RESULTS: In test 1, there were no differences in defecation frequency before and during BB-12 intake, but the quantity of feces increased in the 150g group from 30.5 to 37.4 sticks/11 days ($p<0.05$). Improvement of fecal microflora (higher levels of Bifidobacterium [$p<0.05$] in both groups and lower levels of Clostridium [$p<0.05$] and Streptococcus [$p<0.05$] in 150g/d group), and decrease of fecal ammonia ($p<0.05$) were observed in both 80g/d and 150g/d groups. This suggests that BB-12 yoghurt is efficient in improving the defecation, fecal microflora, and fecal environment depending on amount consumed. In test 2, both stool frequency and fecal quantity were larger during intake than after the intake period ($p<0.05$). There wereno abnormal observations and the safety was considred to be confirmed.

Chr. Hansen A/S -10-12 Bøge Allé – DK-2970 Hørsholm, Denmark - Phone: +45 45 74 74 74 - Fax: +45 45 74 88 88 - www.chr-hansen.com

The information contained herein is to the best of our knowledge true and correct and presented in good faith. It may be subject to change without further notice. To the best of our knowledge this product does not infringe Intellectual Property Rights of any third party. This information is offered solely for your consideration and verification. Copyright© Chr. Hansen A/S. All rights reserved.

Research field: Gastrointestinal Health
Research subfield: Irregular bowel movements
Study type: Human study
Probiotic strain: B. animalis subsp. lactis BB-12
Dosage CFU/day: 4 billion
Product formulation: Fermented milk
Reference number: 0657

Nishida, et al. Effect of Yogurt Containing Bifidobacterium lactis BB-12 on Improvement of Defecation and Fecal Microflora of Healthy Female Adults. Milk Science 2004;53:71-80

Abstract: DESIGN: With the objective to elucidate the effect of yoghurt containing BB-12 on defecation and intestinal flora, 29 healthy adult females (mean age 22 y) with mild (== 5 times / week) constipation consumed 100 g/d of yoghurt with or without BB-12 in a 9 week placebo controlled crossover study consisting of a pre-administration period (2 wk), BB-12/placebo administration period (2 wk), rest period (3 wk) and placebo/BB-12 administration period (2 wk). RESULTS: BB-12 yoghurt improved defecation, intestinal microflora and intestinal environment. In subjects with mild constipation, defecation frequency increased during the BB-12 yoghurt period compared to the pre-administration period ($p<0.01$) and compared to the placebo yoghurt period ($p<0.05$). During the BB-12 yoghurt period, the ratio of Bifidobacterium to total bacteria increased ($p<0.05$), and the ratio of Bacteroidaceae to total bacteria decreased ($p<0.01$) compared to the placebo period. Compared to the pre-administration period, the fecal shape was improved during the BB-12 yoghurt administration period ($p<0.05$) in subjects with mild constipation, and the fecal moisture increased in all subjects ($p<0.05$).

Chr. Hansen A/S -10-12 Bøge Allé – DK-2970 Hørsholm, Denmark - Phone: +45 45 74 74 74 - Fax: +45 45 74 88 88 - www.chr-hansen.com

The information contained herein is to the best of our knowledge true and correct and presented in good faith. It may be subject to change without further notice. To the best of our knowledge this product does not infringe Intellectual Property Rights of any third party. This information is offered solely for your consideration and verification. Copyright© Chr. Hansen A/S. All rights reserved.

Research field: Gastrointestinal Health
Research subfield: Microbiota / recovery
Study type: Human study
Probiotic strain: B. animalis subsp. lactis BB-12
Dosage CFU/day: 5 billion
Product formulation: Other
Reference number: 0444

Ouwehand, et al. Use of a probiotic Bifidobacterium in a dry food matrix, an in vivo study. Int.J.Food Microbiol. 2004;95:103-106

Abstract: Probiotics are commonly included in dairy products. These products require cold storage and transportation, which limits their use. Here, we describe the inclusion of the probiotic strain Bifidobacterium lactis Bb-12 in a dry food matrix, an oat-based cereal bar, and its detection in faeces after consumption of this product. One week after cessation of B. lactis Bb-12 feeding, it could be identified in the faeces of five of the nine subjects.

The information contained herein is to the best of our knowledge true and correct and presented in good faith. It may be subject to change without further notice. To the best of our knowledge this product does not infringe Intellectual Property Rights of any third party. This information is offered solely for your consideration and verification. Copyright© Chr. Hansen A/S. All rights reserved.

CHR HANSEN

Research field: Gastrointestinal Health
Research subfield: Various
Study type: Human study
Probiotic strain: B. animalis subsp. lactis BB-12 and S. thermophilus TH-4
Dosage CFU/day: Mean daily consumption of BB-12 was 41 million per kg bodyweight of child in the high supplementation group and 3.7 million in the low supplemented group.
Product formulation: Milk powder
Reference number: 0435

Saavedra, et al. Long-term consumption of infant formulas containing live probiotic bacteria: tolerance and safety. Am.J.Clin.Nutr. 2004;79:261-267

Abstract: BACKGROUND: Nonpathogenic live bacteria are consumed as food by many children, particularly in the form of yogurt. The tolerance and safety of long-term consumption of specific types and strains of probiotic bacteria are not well documented. OBJECTIVE: The goal was to evaluate tolerance to formulas containing 2 levels of probiotic supplementation and effects on growth, general clinical status, and intestinal health in free-living healthy infants. DESIGN: This was a prospective, double-blind, randomized, placebo-controlled study of healthy infants aged 3-24 mo. Infants were assigned to receive a standard milk-based formula containing 1 x 10(7) colony-forming units (CFU)/g each of Bifidobacterium lactis and Streptococcus thermophilus, formula containing 1 x 10(6) CFU/g each of B. lactis and S. thermophilus, or unsupplemented formula. Clinical outcomes included formula intake, gastrointestinal tolerance, anthropometric measures, daycare attendance, and history of illness. RESULTS: One hundred eighteen infants aged (+/- SD) 7.0 +/- 2.9 mo at enrollment consumed formula for 210 +/- 127 d. There were no significant differences in age, sex, formula consumption, or length of study between groups. The supplemented formulas were well accepted and were associated with a lower frequency of reported colic or irritability (P < 0.001) and a lower frequency of antibiotic use (P < 0.001) than was the unsupplemented formula. There were no significant differences between groups in growth, health care attention seeking, daycare absenteeism, or other health variables. CONCLUSION: Long-term consumption of formulas supplemented with B. lactis and S. thermophilus was well tolerated and safe and resulted in adequate growth, reduced reporting of colic or irritability, and a lower frequency of antibiotic use.

The information contained herein is to the best of our knowledge true and correct and presented in good faith. It may be subject to change without further notice. To the best of our knowledge this product does not infringe Intellectual Property Rights of any third party. This information is offered solely for your consideration and verification. Copyright© Chr. Hansen A/S. All rights reserved.

CHR. HANSEN

Research field: Gastrointestinal Health
Research subfield: Diarrhea
Study type: Human study
Probiotic strain: B. animalis subsp. lactis BB-12 and L. acidophilus LA-5
Dosage CFU/day: > 4.6 billion
Product formulation: Fermented milk
Reference number: 0447

Wang, et al. Effects of ingesting Lactobacillus- and Bifidobacterium-containing yogurt in subjects with colonized Helicobacter pylori. Am.J.Clin.Nutr. 2004;80:737-741

Abstract: Background: Evidence suggests that ingesting lactic acid bacteria exerts a suppressive effect on Helicobacter pylori infection in both animals and humans. Supplementing with Lactobacillus- and Bifidobacterium-containing yogurt (AB-yogurt) was shown to improve the rates of eradication of H. pylori in humans. /// Objective: We administered AB-yogurt to subjects with asymptomatic H. pylori to test whether the yogurt could inhibit H. pylori growth. /// Design: The in vitro inhibition of H. pylori growth was determined by inoculating Lactobacillus acidophilus La5 or Bifidobacterium lactis Bb12 on plates that were inoculated with H. pylori. Assessment of the viability of H. pylori was performed by the mixed culture method with La5 or Bb12. In an intervention study, 59 adult volunteers infected with H. pylori were given AB-yogurt (107 colony-forming units of both La5 and Bb12/mL) twice daily after a meal for 6 wk. Eleven subjects positive for H. pylori infection were treated with milk placebo as control subjects. H. pylori bacterial loads were determined with use of the 13C-urea breath test, which was performed before and 4 and 8 wk after the start of AB-yogurt supplementation. /// Results: Bb12 exerted an in vitro inhibitory effect against H. pylori, whereas La5 did not show an effect. Administration of AB-yogurt decreased the urease activity of H. pylori after 6 wk of therapy (P < 0.0001). /// Conclusion: Regular intake of yogurt containing Bb12 and La5 effectively suppressed H. pylori infection in humans.

The information contained herein is to the best of our knowledge true and correct and presented in good faith. It may be subject to change without further notice. To the best of our knowledge this product does not infringe Intellectual Property Rights of any third party. This information is offered solely for your consideration and verification. Copyright© Chr. Hansen A/S. All rights reserved.

CHR HANSEN

Research field: Other
Research subfield: Various
Study type: Human study
Probiotic strain: B. animalis subsp. lactis BB-12 and L. acidophilus LA-5 and L. bulgaricus LBY-27 and S. thermophilus STY-31
Dosage CFU/day: 12 billion
Product formulation: Capsules
Reference number: 0448

Anderson, et al. Randomized clinical trial of multimodal optimization and standard perioperative surgical care. Br.J.Surg. 2003;90(12):1497-1504

Abstract: Background: Multimodal optimization of surgical care has been associated with reduced hospital stay and improved physical function. The aim of this randomized trial was to compare multimodal optimization with standard care in patients undergoing colonic resection. Methods: Twenty-five patients requiring elective right or left hemicolectomy were randomized to receive a ten-point optimization programme (14 patients) or conventional care (11). The groups were similar in terms of age (64 versus 68 years), male : female sex ratio (6 : 8 versus 5 : 6) and Physiological and Operative Severity Score for the enUmeration of Mortality and morbidity (POSSUM) score (both 26). Outcome measures were recorded before operation and on postoperative days 1, 7 and 30. They included hand grip strength, lung spirometry, and pain and fatigue scores. Further outcome measures included time to achieve a predetermined mobilization target, time to resumption of normal diet, and length of stay. Results: Optimization was associated with maintained grip strength, earlier mobilization (46 versus 69 h; P = 0·043), and significantly lower pain and fatigue scores. Patients in the optimization group tolerated a regular hospital diet significantly earlier than controls (48 versus 76 h; P < 0·001). Optimization significantly reduced the median length of hospital stay (3 versus 7 days; P = 0·002). Conclusion: Optimization of surgical care significantly improved patients' physical and psychological function in the early postoperative period and facilitated early hospital discharge.

The information contained herein is to the best of our knowledge true and correct and presented in good faith. It may be subject to change without further notice. To the best of our knowledge this product does not infringe Intellectual Property Rights of any third party. This information is offered solely for your consideration and verification. Copyright© Chr. Hansen A/S. All rights reserved.

CHR HANSEN

Research field: Gastrointestinal Health
Research subfield: Inflammatory bowel disease / microbiota
Study type: Human study
Probiotic strain: B. animalis subsp. lactis BB-12 and L. acidophilus LA-5
Dosage CFU/day: 50 billion
Product formulation: Fermented milk
Reference number: 0420

Laake, et al. Assessment of mucosal inflammation and circulation in response to probiotics in patients operated with ileal pouch anal anastomosis for ulcerative colitis. Scand.J.Gastroenterol. 2003;38:409-414

Abstract: BACKGROUND: Pouchitis is a common and troublesome condition, and a disturbed microbiological flora and mucosal blood flow in the pouch have been suggested as possible causes. Laser Doppler flowmetry (LDF) has been used successfully to measure gastric and colonic mucosal perfusion in humans. The aim of this study was to evaluate the effect of intervention with probiotics on ileal pouch inflammation and perfusion in the pouch, assessed by endoscopy, histology, fecal calprotectin and LDF. METHODS: A fermented milk product (Cultura; 500 ml) containing live lactobacilli (La-5) and bifidobacteria (Bb-12) was given daily for 4 weeks to 10 patients operated with ileal-pouch-anal anastomosis (IPAA) for ulcerative colitis (UC). Mucosal perfusion was measured with LDF and the degree of inflammation was examined at predefined levels of the distal bowel by endoscopy and histology. Stool samples were cultured for lactobacilli and bifidobacteria and calprotectin were measured before and after intervention. RESULTS: The LDF measurements were reproducible in the pelvic pouch at each of the predefined levels, but did not change after intervention. The mucosal perfusion was reduced in the distal compared to the proximal part of the pouch. Calprotectin levels did not change significantly after intervention. The median endoscopic score for inflammation was significantly reduced by 50% after intervention, whereas the histological score did not change significantly. CONCLUSION: The results suggest that probiotics primarily act superficially, with change of gross appearance of the mucosa at endoscopy, but without significant effect on histological picture, mucosal perfusion or faecal calprotectin, during a relatively short period of 4 weeks.

The information contained herein is to the best of our knowledge true and correct and presented in good faith. It may be subject to change without further notice. To the best of our knowledge this product does not infringe Intellectual Property Rights of any third party. This information is offered solely for your consideration and verification. Copyright© Chr. Hansen A/S. All rights reserved.

CHR HANSEN

Research field: Gastrointestinal Health
Research subfield: Antibiotic side effects
Study type: Human study
Probiotic strain: B. animalis subsp. lactis BB-12 and L. paracasei subsp. paracasei F19
Dosage CFU/day: 50 billion
Product formulation: Fermented milk
Reference number: 0422

Sullivan, et al. Lactobacillus acidophilus, Bifidobacterium lactis and Lactobacillus F19 prevent antibiotic-associated ecological disturbances of Bacteroides fragilis in the intestine. J.Antimicrob.Chemother. 2003;52:308-311

Abstract: OBJECTIVE: The objective of this study was to compare the effect of clindamycin on the intestinal microflora in subjects ingesting yogurt with added probiotic microorganisms with the microflora in subjects ingesting placebo yogurt. MATERIALS AND METHODS: Twenty-four healthy subjects were included in the study. All subjects received 150 mg clindamycin four times daily for 7 days and 250 ml yogurt twice daily for 14 days. Faecal samples were collected before, during and after administration of clindamycin. RESULTS: In the aerobic intestinal microflora, the numbers of enterococci increased after treatment in both groups, whereas other Gram-positive microorganisms decreased. In both groups, the numbers of Escherichia coli also decreased, whereas there was a concomitant increase in numbers of other Gram-negative bacilli. In the anaerobic microflora in subjects receiving yogurt with added microorganisms, the numbers of lactobacilli and bacteroides remained at the same levels throughout the study, whereas the numbers decreased in the placebo group. Other anaerobic bacteria decreased in both groups. The minimum inhibitory concentration of clindamycin against strains of bacteroides increased in both groups during the study. CONCLUSIONS: The probiotic microorganisms evaluated in this study prevented ecological disturbances in the numbers of intestinal Bacteroides fragilis group species during clindamycin administration.

The information contained herein is to the best of our knowledge true and correct and presented in good faith. It may be subject to change without further notice. To the best of our knowledge this product does not infringe Intellectual Property Rights of any third party. This information is offered solely for your consideration and verification. Copyright© Chr. Hansen A/S. All rights reserved.

CHR HANSEN

Research field: Immune Health
Research subfield: Atopic eczema
Study type: Human study
Probiotic strain: B. animalis subsp. lactis BB-12
Dosage CFU/day: 500 billion
Product formulation: Milk powder
Reference number: 0401

Kankaanpaa, et al. Influence of probiotic supplemented infant formula on composition of plasma lipids in atopic infants. J.Nutr.Biochem. 2002;13:364-369

Abstract: Probiotic therapy is a new, successful approach to alleviating allergic symptoms. In this study, our aim was to investigate whether the positive results obtained with probiotic therapy would be associated with the differential absorption and utilization of dietary PUFA. 15 infants referred to a pediatric clinic on the basis of atopic eczema were weaned to Bifidobacterium Bb-12 or Lactobacillus GG supplemented infant formula, or to the same formula without probiotics (randomized, placebo-controlled, double blind study design). In plasma neutral lipids, alpha-linolenic acid (18:3 n-3) proportions were reduced by the probiotic supplementation. In phospholipids, Lactobacillus GG supplemented formula did not influence alpha-linolenic acid proportions, while Bifidobacterium Bb-12 supplemented formula increased the proportion of alpha-linolenic acid; from 0.13 +/- 0.03 to 0.24 +/- 0.03 (mean +/- SEM) (P = 0.002). These results show that some physiological effects of probiotics may be associated with physiological interactions between probiotics and dietary PUFA.

The information contained herein is to the best of our knowledge true and correct and presented in good faith. It may be subject to change without further notice. To the best of our knowledge this product does not infringe Intellectual Property Rights of any third party. This information is offered solely for your consideration and verification. Copyright© Chr. Hansen A/S. All rights reserved.

CHR HANSEN

Research field: Gastrointestinal Health and Immune Health
Research subfield: Various
Study type: Human study
Probiotic strain: B. animalis subsp. lactis BB-12
Dosage CFU/day: 80 billion/day/kg bodyweight
Product formulation: Milk powder
Reference number: 0400

Kirjavainen, et al. Aberrant composition of gut microbiota of allergic infants: a target of bifidobacterial therapy at weaning? Gut 2002;51:51-55

Abstract: BACKGROUND: Recent data have outlined a relationship between the composition of the intestinal microflora and allergic inflammation, and demonstrated the competence of probiotics in downregulation of such inflammation. AIMS: Our aims were to characterise the relationship between gut microbes and the extent of allergic sensitisation and to assess whether the efficacy of bifidobacterial supplementation in the treatment of allergy could relate to modulation of the intestinal microbiota. METHODS: This randomised study included 21 infants with early onset atopic eczema of whom eight were intolerant (highly sensitised group (HSG)) and 13 tolerant (sensitised group (SG)) to extensively hydrolysed whey formula (EHF). In the SG, six were weaned to EHF without (placebo group (PG)) and seven to EHF with Bifidobacterium lactis Bb-12 supplementation (bifidobacteria treated group (BbG)). The faecal microflora of infants in the HSG was analysed only before weaning whereas in the SG the faecal microflora was analysed both before and after weaning. RESULTS: Infants in the HSG had greater numbers of lactobacilli/enterococci than those in the SG. Serum total IgE concentration correlated directly with Escherichia coli counts in all infants and with bacteroides counts in the HSG, indicating that the presence of these bacteria is associated with the extent of atopic sensitisation. The effect of supplementation was characterised as a decrease in the numbers of Escherichia coli and protection against an increase in bacteroides numbers during weaning. CONCLUSIONS: These data indicate that bifidobacterial supplementation appears to modify the gut microbiota in a manner that may alleviate allergic inflammation. Further studies are needed to confirm this conclusion.

The information contained herein is to the best of our knowledge true and correct and presented in good faith. It may be subject to change without further notice. To the best of our knowledge this product does not infringe Intellectual Property Rights of any third party. This information is offered solely for your consideration and verification. Copyright© Chr. Hansen A/S. All rights reserved.

CHR HANSEN

Research field: Gastrointestinal Health
Research subfield: Microbiota
Study type: Human study
Probiotic strain: B. animalis subsp. lactis BB-12
Dosage CFU/day: 30 billion
Product formulation: Fermented milk
Reference number: 0417

Malinen, et al. PCR-ELISA II: Analysis of Bifidobacterium populations in human faecal samples from a consumption trial with Bifidobacterium lactis Bb-12 and a galacto-oligosaccharide preparation. Syst.Appl.Microbiol. 2002;25:249-258

Abstract: A PCR-ELISA method was extended for detection of most common Bifidobacterium species in humans and applied to a feeding trial including administration of Bifidobacterium lactis Bb-12 and galacto-oligosaccharide (GOS)-containing syrup as probiotic and prebiotic preparations, respectively. For PCR-ELISA, oligonucleotide probes based on 16S rDNA sequences were designed and tested for specificity and sensitivity with nine different bifidobacterial species followed by analysis of faecal samples. Bifidobacteria were monitored for their fluctuations during and after the feeding trial. Bifidobacterium longum was the most common species found in the faecal samples, followed by B. adolescentis and B. bifidum. During ingestion of the probiotic B. lactis Bb-12, the strain appeared in the faeces but was absent again one week after finishing of the trial. The species that were observed in the faecal samples taken prior to the feeding experiments persisted also in samples derived from the pre-feeding and feeding periods. The most consistent change observed was the decrease in the relative amount of B. longum in the test group ingesting either B. lactis Bb-12 alone or in combination with GOS-syrup. Since the amounts of B. longum increased again in the post-feeding sample with these subjects, it may suggest that to some extent B. lactis Bb-12 is able to transiently replace B. longum.

The information contained herein is to the best of our knowledge true and correct and presented in good faith. It may be subject to change without further notice. To the best of our knowledge this product does not infringe Intellectual Property Rights of any third party. This information is offered solely for your consideration and verification. Copyright© Chr. Hansen A/S. All rights reserved.

CHR HANSEN

Research field: Other
Research subfield: Growth
Study type: Human study
Probiotic strain: B. animalis subsp. lactis BB-12
Dosage CFU/day: > 12 billion BB-12 or 12 billion BB-12 / S. thermophilus
Product formulation: Milk powder
Reference number: 0411

Nopchinda, et al. Effect of bifidobacterium Bb12 with or without Streptococcus thermophilus supplemented formula on nutritional status. J.Med.Assoc.Thai. 2002;85 Suppl 4:S1225-31

Abstract: Acute diarrhea is a common cause of infant morbidity and mortality. Probiotic supplemented infant formula is one of the effective methods for prevention of rotavirus diarrhea. Other benefits of the probiotics supplemented formula were evaluated by monitoring the growth of the children. A double-blind, placebo-controlled trial was done in 148 children aged 6-36 months. They were divided into 3 groups: the Bb12 group, 51 children received infant formula with Bifidobacteria Bb12 supplement; the Bb12+ST group, 54 children received infant formula with Bb12 and Streptococcus thermophilus supplement; and the control group, 43 children received infant formula without supplement. The mean weight Z-score according to WHO reference standard of the Bbl2 group was -1.8 +/- 0.12, the Bb12+ST group was -1.4 +/- 0.11 and the control group was -1.8 +/- 0.13 at entry. The mean weight Z-score of children after 6 month showed that the children in the Bbl2+ST group had the highest increase in weight which was increased from -1.4 +/- 0.11 to -0.9 +/- 0.12 compared to the Z-score of the Bb12 group which had increased from -1.8 +/- 0.12 to -1.2 +/- 0.13 and in the control group from -1.8 +/- 0.13 to -1.7 +/- 0.25. In terms of the mean height Z-score, the Bb12 group was -2.7 +/- 0.14 to -1.7 +/- 0.16 which was higher than the Bb12+ST group (- 2.2 +/- 0.13 to -1.7 +/- 0.13) but was not different from the control group. However, the mean weight/height Z-score of the Bbl2+ST group had approached the reference standard (Bb12 group -0.1 +/- 0.11 to -0.1 +/- 0.13, Bb12+ST group -0.1 +/- 0.10 to 0.3 +/- 0.17, control group -0.4 +/- 0.12 to -0.1 +/- 0.16). Data showed that children who received the probiotics supplement formula had better growth during the 6 month period.

Chr. Hansen A/S -10-12 Bøge Allé – DK-2970 Hørsholm, Denmark - Phone: +45 45 74 74 74 - Fax: +45 45 74 88 88 - www.chr-hansen.com

The information contained herein is to the best of our knowledge true and correct and presented in good faith. It may be subject to change without further notice. To the best of our knowledge this product does not infringe Intellectual Property Rights of any third party. This information is offered solely for your consideration and verification. Copyright© Chr. Hansen A/S. All rights reserved.

CHR HANSEN

Research field: Gastrointestinal Health
Research subfield: Diarrhea
Study type: Human study
Probiotic strain: B. animalis subsp. lactis BB-12 and L. acidophilus LA-5
Dosage CFU/day: > 10 billion
Product formulation: Fermented milk
Reference number: 0412

Sheu, et al. Impact of supplement with Lactobacillus- and Bifidobacterium-containing yogurt on triple therapy for Helicobacter pylori eradication. Aliment.Pharmacol.Ther. 2002;16:1669-1675

Abstract: Aim : To test whether supplements of Lactobacillus- and Bifidobacterium-containing yogurt (AB-Yogurt) affect the success of Helicobacter pylori eradication. /// Methods : One hundred and sixty H. pylori-infected patients were randomized into a triple-plus-yogurt group or a triple-only group, receiving 1 week of triple therapy with and without supplements of AB-Yogurt, respectively. In the triple-plus-yogurt group, AB-Yogurt was continued for 4 weeks after triple therapy. Eight weeks later, patients were assessed for the success of H. pylori eradication. The stool samples of 22 randomly selected patients, 11 from each group, were provided on enrolment, at the first week and at the fifth week for evaluation of the percentage of Bifidobacterium in anaerobes. /// Results : By intention-to-treat analysis, the triple-plus-yogurt group had a higher H. pylori eradication rate than the triple-only group (91% vs. 78%, P < 0.05). The per protocol H. pylori eradication rates were similar for both groups (93.5% vs. 89%, P = N.S.). Only patients supplemented with AB-Yogurt showed restoration of the percentage of Bifidobacterium in the anaerobes of stools at the fifth week to the level in the stools on enrolment. /// Conclusions : Supplement with AB-Yogurt can improve the intention-to-treat eradication rates of H. pylori, and can restore the depletion of Bifidobacterium in stools after triple therapy.

The information contained herein is to the best of our knowledge true and correct and presented in good faith. It may be subject to change without further notice. To the best of our knowledge this product does not infringe Intellectual Property Rights of any third party. This information is offered solely for your consideration and verification. Copyright© Chr. Hansen A/S. All rights reserved.

CHR HANSEN

Research field: Gastrointestinal Health
Research subfield: Microbiota / recovery
Study type: Human study
Probiotic strain: B. animalis subsp. lactis BB-12
Dosage CFU/day: 30 billion
Product formulation: Fermented milk
Reference number: 0416

Alander. Effect of galacto-oligosaccharide supplementation on human faecal microflora and on survival and persistence of Bifidobacterium lactis Bb-12 in the gastrointestinal tract. Int.Dairy J. 2001;11(10):817-825

Abstract: Galacto-oligosaccharides (GOS) are considered to have bifidogenic properties in humans. To study the effect of GOS-containing syrup (60% GOS) alone or together with the probiotic strain *Bifidobacterium lactis* Bb-12 on selected components of the faecal flora, and the effect of GOS supplementation on colonisation of *B. lactis* Bb-12, a feeding trial on 30 healthy volunteers was performed. Mean numbers of bifidobacteria increased slightly in all study groups during the feeding period. Isolates having the identical RAPD genotype with *B. lactis* Bb-12 were detected in high numbers in the Bb-12 group and in the GOS-containing syrup+Bb-12 group indicating a good survival of *B. lactis* Bb-12 through the gastrointestinal tract. No differences in the prevalence or numbers of isolates with *B. lactis* Bb-12 genotype could be observed between groups suggesting that GOS-containing syrup did not enhance the survival or persistence of *B. lactis* Bb-12 in the gut.

The information contained herein is to the best of our knowledge true and correct and presented in good faith. It may be subject to change without further notice. To the best of our knowledge this product does not infringe Intellectual Property Rights of any third party. This information is offered solely for your consideration and verification. Copyright© Chr. Hansen A/S. All rights reserved.

Research field: Gastrointestinal Health
Research subfield: Irregular bowel movements
Study type: Human study
Probiotic strain: B. animalis subsp. lactis BB-12
Dosage CFU/day: 5 billion or 15 billion
Product formulation: Fermented milk
Reference number: 0655

Matsumoto, et al. Effect of Yoghurt with Bifidobacterium lactis LKM512 in Improving Fecal Microflora and Defecation of Healthy Volunteers. Intestine Microbiology Magazine 2001;14:97-102

Abstract: DESIGN: The effects of Bifidobacterium lactis LMK512 (BB-12) on fecal properties, defecation frequency and fecal microflora were examined in 30 healthy, volunteers (average age of 30 y), with defecation frequency of 0.05). There were no significant changes in in fecal color, but with a tendency toward decreased hard feces and increased soft feces with both 100 g/d and 300 g/d. Bifidobacterium number increased during BB-12 administration ($p<0.05$), but not during placebo administration. Detection rate of Clostridium was reduced both by BB-12 and placebo. No substatial changes were observed in major bacteria (Bacteriodaceaea, Eubacterium, Clostridium, Enterobacteraceae).

The information contained herein is to the best of our knowledge true and correct and presented in good faith. It may be subject to change without further notice. To the best of our knowledge this product does not infringe Intellectual Property Rights of any third party. This information is offered solely for your consideration and verification. Copyright© Chr. Hansen A/S. All rights reserved.

Research field: Gastrointestinal Health
Research subfield: Various
Study type: Human study
Probiotic strain: B. animalis subsp. lactis BB-12
Dosage CFU/day: 5.2 billion
Product formulation: Fermented milk
Reference number: 0409

Matsumoto, et al. Impact of LKM512 yogurt on improvement of intestinal environment of the elderly. FEMS Immunol.Med.Microbiol. 2001;31:181-186

Abstract: Improvement of the intestinal environment by administration of LKM512 yogurt was examined using polyamine, haptoglobin and mutagenicity as indexes which directly reflect the health condition of the host. The concentration of spermine in feces increased significantly by 3-fold ($P<0.05$) at week 2 of administration of LKM512 yogurt compared with before administration, and that of putrescine, spermidine, and cadaverine also tended to increase with administration of LKM512 yogurt. The haptoglobin content in feces decreased significantly ($P<0.05$) at week 2 of administration of LKM512 yogurt, and it showed a negative correlation with the polyamine content, indicating that acute intestinal inflammation was suppressed. Fecal mutagenicity was measured using fecal extract and fecal precipitate. Both preparations showed similar significant decreases ($P<0.05$) by the administration of LKM512 yogurt, as well as a negative correlation with polyamine content. This result indicated that antimutagenicity due to administration of LKM512 yogurt was not based on binding of the mutagen to the bacterial cell wall. Many reports have suggested that polyamines increased by the administration of LKM512 yogurt led to inhibition of inflammation and antimutagenicity in the intestinal tract.

Chr. Hansen A/S -10-12 Bøge Allé – DK-2970 Hørsholm, Denmark - Phone: +45 45 74 74 74 - Fax: +45 45 74 88 88 - www.chr-hansen.com

The information contained herein is to the best of our knowledge true and correct and presented in good faith. It may be subject to change without further notice. To the best of our knowledge this product does not infringe Intellectual Property Rights of any third party. This information is offered solely for your consideration and verification. Copyright© Chr. Hansen A/S. All rights reserved.

CHR HANSEN

Research field: Immune Health
Research subfield: Atopic eczema
Study type: Human study
Probiotic strain: B. animalis subsp. lactis BB-12 and L. rhamnosus LGG
Dosage CFU/day: Child-dependent
Product formulation: -
Reference number: 0388

Isolauri, et al. Probiotics in the management of atopic eczema. Clin.Exp.Allergy 2000;30(11):1604-1610

Abstract: BACKGROUND: Over the last two decades the incidence of allergic diseases has increased in industrialized countries, and consequently new approaches have to be explored. OBJECTIVE: The potential of probiotics to control allergic inflammation at an early age was assessed in a randomized double-blind placebo-controlled study. METHODS: A total of 27 infants, mean age 4.6 months, who manifested atopic eczema during exclusive breast-feeding and who have had no exposure to any infant or substitute formula were weaned to probiotic-supplemented, Bifidobacterium lactis Bb-12 or Lactobacillus strain GG (ATCC 53103), extensively hydrolysed whey formulas or to the same formula without probiotics. The extent and severity of atopic eczema, the growth and nutrition of infants, and concentrations of circulating cytokines/chemokines and soluble cell surface adhesion molecules in serum and methyl-histamine and eosinophilic protein X in urine were determined. RESULTS: The SCORAD score reflecting the extent and severity of atopic eczema was 16 (7-25) during breast-feeding, median (interquartile range). After 2 months, a significant improvement in skin condition occurred in patients given probiotic-supplemented formulas, as compared to the unsupplemented group; chi(2) = 12.27, P = 0.002. SCORAD decreased in the Bifidobacterium lactis Bb-12 group to 0 (0-3.8), and in the Lactobacillus GG group to 1 (0.1-8.7), vs unsupplemented 13.4 (4.5-18.2), median (interquartile range), in parallel with a reduction in the concentration of soluble CD4 in serum and eosinophilic protein X in urine. CONCLUSION: The results provide the first clinical demonstration of specific probiotic strains modifying the changes related to allergic inflammation. The data further indicate that probiotics may counteract inflammatory responses beyond the intestinal milieu. The combined effects of these probiotic strains will guide infants through the weaning period, when sensitization to newly encountered antigens is initiated. The probiotic approach may thus offer a new direction in the search for future foods for allergy treatment and prevention strategies.

The information contained herein is to the best of our knowledge true and correct and presented in good faith. It may be subject to change without further notice. To the best of our knowledge this product does not infringe Intellectual Property Rights of any third party. This information is offered solely for your consideration and verification. Copyright© Chr. Hansen A/S. All rights reserved.

Research field: Gastrointestinal Health
Research subfield: Irregular bowel movements
Study type: Human study
Probiotic strain: B. animalis subsp. lactis BB-12 and L. acidophilus LA-5
Dosage CFU/day: 1 billion BB-12 and 0.3 billion LA-5
Product formulation: Fermented milk
Reference number: 0531

Shioya, et al. Effect of fermented milk containing Bifidobacterium lactis FK 120 on the fecal flora and fecal properties in healthy female volunteers. Food Health and Nutrition Research (Journal of Nutritional Food) 2000:18-18

Abstract: DESIGN: In a crossover study, 46 healthy female volunteers with the average age of 19.7 y were given 100 mL/d of fermented milk containing Bifidobacterium lactis FK 120 (BB-12, Lactobacillus acidophilus FK 205 (LA-5), Streptococcus thermophilus FK 303 (TH-3) and Sterptococcus thermophilus FK 320 (ST-20)and 100 mL/d of pure milk as placebo for up to ten days in order to examine the effect on fecal properties and defecation frequencies. The fecal microflora, pH, water content and ammonia concentration were also examined in 10 subjects (mean age 19.8 y). RESULTS: When comparing the BB-12 period with the placebo period, BB-12 was effective in increasing the fecal amount and in changing the fecal properties positively. The ratio of bifidobacteria to total bacteria was increased, and the frequency of Clostridium prefringens was decreased, as was the fecal pH. There was a tendency towards a decreased ammonia concentration and towards increased water content. It should be noted that the authors only highlight BB-12.

The information contained herein is to the best of our knowledge true and correct and presented in good faith. It may be subject to change without further notice. To the best of our knowledge this product does not infringe Intellectual Property Rights of any third party. This information is offered solely for your consideration and verification. Copyright© Chr. Hansen A/S. All rights reserved.

Research field: Gastrointestinal Health
Research subfield: Irregular bowel movements
Study type: Human study
Probiotic strain: B. animalis subsp. lactis BB-12 and L. acidophilus LA-5
Dosage CFU/day: 1 billion BB-12 and 0.3 billion LA-5 or 3 billion BB-12 and 0.9 billion LA-5
Product formulation: Fermented milk
Reference number: 0530

Shioya, et al. Effect of fermented milk containing Bifidobacterium lactis FK 120 on the fecal flora, with special reference to Bifidum species, and fecal properties in healthy volunteers. Food Health and Nutrition Research (Journal of Nutritional Food) 2000:19-32

Abstract: DESIGN: The effects of Bifidobacterium lactis FK 120 (BB-12), Lactobacillus acidophilus FK 320 (LA-5), Streptococcus thermophilus FK 303 (TH-3) and Streptococcus thermophilus FK 320 (ST20) on fecal properties and defecation frequency were examined in 48 healthy, volunteers with the average age of 42.0 y, who were given 100 mL/d or 300 mL/d of fermented milk containing BB-12 for two weeks. Additionally, the effect of BB-12 on the composition of Bifidobacterium species in fecal microflora and on fecal properties were examined in 7 volunteers with the average age of 45.7 y. RESULTS: The intake of fermented milk containing BB-12 improved the fecal flora without a change in the composition of Bifidobacterium species. During the intake period, increase in defecation frequency and fecal amount, and improvement of fecal shape and coloring were observed. The ratio of Bifidobacteria to total bacteria was increased by the intake. The frequency of C. perfringes and Clostridium-others as well as fecal ammonia ($P<0.01$) was decreased by the intake. BB-12 was detected in the feces of four volunteers during the BB-12 administration with 107.3-8.8 cfu/g. In the composition of fecal Bifidobacterium species, the high incidence if B. adolescentis and B. longum were observed throughout the experimental periods. It should be noted that the authors only highlight BB-12.

The information contained herein is to the best of our knowledge true and correct and presented in good faith. It may be subject to change without further notice. To the best of our knowledge this product does not infringe Intellectual Property Rights of any third party. This information is offered solely for your consideration and verification. Copyright© Chr. Hansen A/S. All rights reserved.

CHR HANSEN

Research field: Gastrointestinal Health
Research subfield: Irregular bowel movements
Study type: Human study
Probiotic strain: B. animalis subsp. lactis BB-12 and L. acidophilus LA-5
Dosage CFU/day: 1 billion BB-12 and 0.3 billion LA-5
Product formulation: Fermented milk
Reference number: 0532

Shioya, et al. Effect of fermented milk containing Bifidobacterium lactis FK 120 on the fecal flora, with special reference to Bifidum species, and fecal properties in elderly volunteers. Food Health and Nutrition Research (Journal of Nutritional Food) 2000:33-44

Abstract: DESIGN: Six elderly volunteers with the average age of 81.7 y were given 100 mL/d of fermented milk containing Bifidobacterium lactis FK 120 (BB-12), Lactobacillus acidophilus FK 205 (LA-5), Streptococcus thermophilus FK 303 (TH-3) and Streptococcus thermophilus FK 320 (ST-20) for 2 weeks in order to examine the effect of BB-12 on fecal flora and fecal properties. RESULTS: BB-12 was detected within the range of 10^6.6-8.9 cfu/g in the feces of five of the six volunteers. Although a high incidence of B. adolescentis was detected before the intake, BB-12 was predominant during the intervention period. The ratio of Bifidobacteria to fecal bacteria was significantly increased (P<0.05), and the number of Clostridium perfringens and Clostridium-others was significantly decreased (P<0.05). Furthermore, an improvement of diarrhea with decreased fecal water, and a significant (P<0.05) decrease of fecal odor was observed. It should be noted that the authors only highlight BB-12.

The information contained herein is to the best of our knowledge true and correct and presented in good faith. It may be subject to change without further notice. To the best of our knowledge this product does not infringe Intellectual Property Rights of any third party. This information is offered solely for your consideration and verification. Copyright© Chr. Hansen A/S. All rights reserved.

Research field: Gastrointestinal Health
Research subfield: Inflammatory Bowel Disease / irregular bowel movements / microbiota
Study type: Human study
Probiotic strain: B. animalis subsp. lactis BB-12 and L. acidophilus LA-5
Dosage CFU/day: > 50 billion or < 5 million (heat-treated)
Product formulation: -
Reference number: 0383

Laake, et al. Influence of fermented milk on clinical state, fecal bacterial counts and biochemical characteristics in patients with ileal- pouch- anal-anastomosis. Microb.Ecol.Health Dis. 1999;11:211-217

Abstract: Pouchitis is a condition with acute or chronic inflammation in the ileal pouch of colectomized patients. A disturbed microecology in the pouch has been suggested as a possible cause. The present study was an attempt to investigate if a fermented milk product containing live lactobacilli and bifidobacteria might influence on the intestinal microecology of IPAA-patients. Five hundred ml of a fermented milk product containing live lactobacilli (La-5) and bifidobacteriae (Bb-12) (Cultura, TM), was given in its regular form or heat-treated, in a randomized, double blinded fashion, to 16 patients operated on with ileal- pouch- anal-anastomosis (IPAA) for ulcerative colitis (UC). Symptoms recordings, stool cultures and determination of 6 different microflora associated characteristics (MACs);(short-chain fatty acids (SCFAs), urobilinogen, mucin degradation, coprostanol, fecal tryptic activity (FTA), and beta-aspartyl glycin) were performed at inclusion, after 1 and 2 weeks. In the group given regular Cultura, daily stool frequency was reduced from median 5.8 to 2.8. It was unchanged in the group given heat-treated Cultura. With regular Cultura the number of lactobacilli increased significantly during intervention, and remained elevated one week thereafter. The number of bifidobacteriae increased significantly during intervention, but was back to baseline one week thereafter. With heat-treated Cultura, no significant change was observed. Significant reduction in stool frequency was observed among the patients given regular Cultura. No change was observed among those given heat-treated Cultura. No major effects on six MACs were observed.

The information contained herein is to the best of our knowledge true and correct and presented in good faith. It may be subject to change without further notice. To the best of our knowledge this product does not infringe Intellectual Property Rights of any third party. This information is offered solely for your consideration and verification. Copyright© Chr. Hansen A/S. All rights reserved.

CHR HANSEN

Research field: Immune Health
Research subfield: Immune stimulation
Study type: Human study
Probiotic strain: B. animalis subsp. lactis BB-12
Dosage CFU/day: NA
Product formulation: Milk powder
Reference number: 0413

Phuapradit, et al. Reduction of rotavirus infection in children receiving bifidobacteria-supplemented formula. J.Med.Assoc.Thai. 1999;82 Suppl 1:S43

Abstract: This study was conducted at Pakkred Babies Home, Bangkok, Thailand; with the hypothesis that children receiving probiotic-supplemented milk-based formula may be protected from developing diarrheal diseases. Salivary rotavirus-specific IgA antibody was used as an indicator of rotavirus infection. One hundred and seventy-five children, aged 6-36 months, were enrolled in the study. They were divided into 3 groups according to the type of formula given. There were 81 episodes of diarrhea during an 8-month study period, most of which were caused by bacterial enteropathogens. Ninety-seven pairs of salivary samples were adequate for the analysis of rotavirus antibody. Among 23 children receiving milk-based follow-up formula and serving as control group, 30.4 per cent of them had > or = 4-fold increase in the antibody titre, indicating subclinical rotavirus infection. The majority of children in the other 2 study groups, receiving the same formula supplemented with either Bifidobacterium Bb12 alone or together with Streptococcus thermophilus, had no significant change in the antibody titres between the two time points. The results of this study support our hypothesis that children receiving bifidobacteria-supplemented milk-based formula may be protected against symptomatic rotavirus infection.

The information contained herein is to the best of our knowledge true and correct and presented in good faith. It may be subject to change without further notice. To the best of our knowledge this product does not infringe Intellectual Property Rights of any third party. This information is offered solely for your consideration and verification. Copyright© Chr. Hansen A/S. All rights reserved.

CHR HANSEN

Research field: Gastrointestinal Health and Immune Health
Research subfield: Microbiota / Immune stimulation / recovery
Study type: Human study
Probiotic strain: B. animalis subsp. lactis BB-12
Dosage CFU/day: 1 billion
Product formulation: Milk powder
Reference number: 0332

Fukushima, et al. Effect of a probiotic formula on intestinal immunoglobulin A production in healthy children. Int.J.Food Microbiol. 1998;42:39-44

Abstract: The anti-infectious effect of probiotics has recently been reported and one mechanism may be the non-specific stimulation of immunity. This study was performed to elucidate the influence of a probiotic formula on intestinal microflora and local immunity in healthy children. A follow-up formula containing viable bifidobacteria was given to seven healthy Japanese children (15 to 31 months old) for 21 days. During intake of the formula, the administered strain was detected in feces from five subjects (71%) and total fecal bifidobacteria slightly increased. Fecal levels of total IgA and anti-poliovirus IgA during intake of the formula were significantly higher than those before intake ($P < 0.05$). The increase in local IgA levels resulting from ingestion of the probiotic formula may contribute to enhancement of the mucosal resistance against gastrointestinal infections.

The information contained herein is to the best of our knowledge true and correct and presented in good faith. It may be subject to change without further notice. To the best of our knowledge this product does not infringe Intellectual Property Rights of any third party. This information is offered solely for your consideration and verification. Copyright© Chr. Hansen A/S. All rights reserved.

CHR HANSEN

Research field: Gastrointestinal Health
Research subfield: Diarrhea / Microbiota
Study type: Human study
Probiotic strain: B. animalis subsp. lactis BB-12 and L. acidophilus LA-5 and L. bulgaricus LBY-27 and S. thermophilus STY-31
Dosage CFU/day: 21 billion
Product formulation: Capsules
Reference number: 0186

Nord, et al. Oral supplementation with lactic acid-producing bacteria during intake of clindamycin. Clin.Microbiol.Infect. 1997:124-132

Abstract: OBJECTIVE: To study the effect of administration of clindamycin with or without supplementation of the intestinal microflora with Bifidobacterium bifidum and Lactobacillus acidophilus. METHODS: Twenty-three healthy subjects received clindamycin by mouth for 7 days. Eleven of the subjects also received capsules containing lyophilized L. acidophilus and B. bifidum for 14 days. The other 12 subjects received placebo. RESULTS: There was a marked decrease in total numbers of anaerobic bacteria during the administration of clindamycin. In the lactic acid bacteria-supplemented group, a tendency towards delayed reduction and earlier increase in bifidobacteria was observed, and two of 11 subjects (18%) were colonized with Clostridium difficile, in comparison with five of 12 (41%) in the placebo group. The total number of microorganisms was significantly higher in the lactic acid bacteria-supplemented group than in the placebo group (p=0.02) 4 days after the end of clindamycin administration. The difference was mainly due to higher counts of Escherichia coli and enterococci. Mean levels of other enterobacteria increased less in the lactic acid bacteria-supplemented group than in the placebo group between days 0 and 14. CONCLUSIONS: The recolonization with aerobic and anaerobic microorganisms was faster in the lactic acid bacteria-supplemented group than in the placebo group. This may be of importance in preventing colonization with C. difficile.

Chr. Hansen A/S -10-12 Bøge Allé – DK-2970 Hørsholm, Denmark - Phone: +45 45 74 74 74 - Fax: +45 45 74 88 88 - www.chr-hansen.com

The information contained herein is to the best of our knowledge true and correct and presented in good faith. It may be subject to change without further notice. To the best of our knowledge this product does not infringe Intellectual Property Rights of any third party. This information is offered solely for your consideration and verification. Copyright© Chr. Hansen A/S. All rights reserved.

CHR HANSEN

Research field: Gastrointestinal Health and Immune Health
Research subfield: Diarrhea / infections
Study type: Human study
Probiotic strain: B. animalis subsp. lactis BB-12 and L. acidophilus LA-5 and L. bulgaricus LBY-27 and S. thermophilus STY-31
Dosage CFU/day: 9 billion
Product formulation: Capsules
Reference number: 0306

Black, et al. Report from a double-blind placebo-controlled trial with HIP against traveler's diarrhea with a new dosage regimen. Internal report. Internal report 1995

Abstract: DESIGN: In a placebo-controlled, double-blind study, 107 tourists consumed a probiotic capsules at breakfast and dinner during a 13 day trip to Tunisia. Treatment began 2 days prior to departure and continued until 2 days after the trip. RESULTS: ParticipantsÃ¢â‚¬â„¢ subjective evaluation of the effect (recorded as accompanying symptoms) showed the general condition of the probiotic group was statistically significantly better during the trial. However, overall results indicate that 3 (vs. 2) capsules per day is more effective on traveler's diarrhea as seen in previous trials. Microbiological analyses of fecal samples appeared to show an effect against Candida infection (P=0.07).

Chr. Hansen A/S -10-12 Bøge Allé – DK-2970 Hørsholm, Denmark - Phone: +45 45 74 74 74 - Fax: +45 45 74 88 88 - www.chr-hansen.com

The information contained herein is to the best of our knowledge true and correct and presented in good faith. It may be subject to change without further notice. To the best of our knowledge this product does not infringe Intellectual Property Rights of any third party. This information is offered solely for your consideration and verification. Copyright© Chr. Hansen A/S. All rights reserved.

Research field: Gastrointestinal Health
Research subfield: Recovery / Microbiota / Safety / Stability
Study type: Human study
Probiotic strain: B. animalis subsp. lactis BB-12
Dosage CFU/day: Child-dependent
Product formulation: Milk powder
Reference number: 0221

Langhendries, et al. Effect of a fermented infant formula containing viable bifidobacteria on the fecal flora composition and pH of healthy full-term infants. J.Pediatr.Gastroenterol.Nutr. 1995;21:177-181

Abstract: We assessed the growth, tolerance, and acceptability as well as fecal flora composition and stool pH of 20 healthy full-term infants fed with a fermented whey-adapted infant formula containing viable bifidobacteria (10(6)/g of powder) during the first 2 months of life. This fermented infant formula, first biologically acidified by Streptococcus thermophilus and Lactobacillus helveticus, was compared to a whey-adapted, nonacidified, low-phosphate infant formula in a double-blind, randomized controlled study. The results were compared to a control group (n = 14) of fully breast-fed infants. The fermented whey-adapted formula containing viable bifidobacteria induced a prevalence of colonization with bifidobacteria at 1 month of age similar to that of breast-fed infants (12/20 versus 8/14) but significantly higher than in the group fed the standard infant formula (4/20). The mean bacterial count of bifidobacteria was similar in all colonized infants; however, fecal pH was significantly lower in the breast-fed infants than in the nonacidified bottle-fed infants. This kind of infant formula was well tolerated and promoted a normal growth during the first 2 months.

The information contained herein is to the best of our knowledge true and correct and presented in good faith. It may be subject to change without further notice. To the best of our knowledge this product does not infringe Intellectual Property Rights of any third party. This information is offered solely for your consideration and verification. Copyright© Chr. Hansen A/S. All rights reserved.

CHR HANSEN

Research field: Gastrointestinal Health
Research subfield: Recovery
Study type: Human study
Probiotic strain: B. animalis subsp. lactis BB-12
Dosage CFU/day: 10 billion (Nestle LA-1: 70 billion)
Product formulation: Fermented milk
Reference number: 0206

Schiffrin, et al. Immunomodulation of human blood cells following the ingestion of lactic acid bacteria. J.Dairy Sci. 1995;78:491-497

Abstract: Because of the lack of data that convincingly show immunomodulatory properties of lactic acid bacteria in humans, a study was performed in which healthy volunteers were divided into two groups and given a fermented milk product supplemented with Lactobacillus acidophilus strain La1 or Bifidobacterium bifidum strain Bb 12 for 3 wk. Blood was sampled throughout the study to assess changes in lymphocyte subsets or leukocyte phagocytic activity following consumption of the fermented products. No modifications of lymphocyte subpopulations were detected. In contrast, phagocytosis of Escherichia coli sp. in vitro was enhanced after the administration of both fermented products. The increment in phagocytosis was coincident with fecal colonization by the lactic acid bacteria and persisted for 6 wk after ingestion of the fermented products. By this time, the fecal lactobacilli and bifidobacteria had returned to concentrations prior to consumption. Nonspecific, anti-infective mechanisms of defense can be enhanced by the ingestion of specific lactic acid bacteria strains. These strains can be used as nutritional supplements to improve the immune function of particular age groups, i.e., the neonate or the elderly, for which these functions are diminished.

The information contained herein is to the best of our knowledge true and correct and presented in good faith. It may be subject to change without further notice. To the best of our knowledge this product does not infringe Intellectual Property Rights of any third party. This information is offered solely for your consideration and verification. Copyright© Chr. Hansen A/S. All rights reserved.

CHR HANSEN

Research field: Gastrointestinal Health
Research subfield: Recovery / Microbiota / Lactose intolerance
Study type: Human study
Probiotic strain: Part I: B. animalis subsp. lactis BB-12, Part II: B. animalis subsp. lactis BB-12 and L. acidophilus LA-5, Part III: B. animalis subsp. lactis BB-12 and L. acidophilus LA-5 and L. bulgaricus LBY-27 and S. thermophilus STY-31
Dosage CFU/day: Part I: 24 billion, Part III: 42 billion
Product formulation: Capsules
Reference number: 0090

Hove, et al. Effect of lactic acid bacteria on the intestinal production of lactate and short-chain fatty acids, and the absorption of lactose. Am.J.Clin.Nutr. 1994;59:74-79

Abstract: The characteristic fermentation pattern seen with specific saccharides in incubations with pure cultures of Lactobacillus acidophilus and Bifidobacterium bifidum disappeared when incubated in 16.7% fecal homogenate. The productions of lactate and short-chain fatty acids in mixed bacterial-fecal incubations were similar to productions in fecal homogenates without L acidophilus and B bifidum and were mainly associated with the specific mono-, di-, and polysaccharides added to the incubate. B bifidum was cultured from ileostomic contents in eight of nine ileostomists after oral administration (2.4 x 10(10) cells), but did not influence the concentrations and productions of DL-lactate and short-chain fatty acids in the ileostomic outputs and incubates. Large amounts of ingested lactic acid bacteria (4.2 x 10(10) cells) did not ameliorate lactose malabsorption measured by the breath-hydrogen test in 12 lactose malabsorbers. This study shows that ingested lactic acid bacteria are indeed present in the colon, but it does not support the theory that they change the pattern of colonic fermentation or the degree of intestinal lactose malabsorption.

The information contained herein is to the best of our knowledge true and correct and presented in good faith. It may be subject to change without further notice. To the best of our knowledge this product does not infringe Intellectual Property Rights of any third party. This information is offered solely for your consideration and verification. Copyright© Chr. Hansen A/S. All rights reserved.

CHR HANSEN

Research field: Gastrointestinal Health and Immune Health
Research subfield: Microbiota / Immune stimulation
Study type: Human study
Probiotic strain: B. animalis subsp. lactis BB-12
Dosage CFU/day: 4 - 40 billion
Product formulation: Fermented milk
Reference number: 0208

Link-Amster, et al. Modulation of a specific humoral immune response and changes in intestinal flora mediated through fermented milk intake. FEMS Immunol.Med.Microbiol. 1994;10:55-63

Abstract: This study was undertaken to elucidate whether eating a fermented milk containing Lactobacillus acidophilus La1 and bifidobacteria could induce changes in intestinal flora and modulate the immune response in man. Volunteers consumed a fermented milk containing L. acidophilus La1 and bifidobacteria over a period of three weeks during which an attenuated Salmonella typhi Ty21a was administered to mimic an enteropathogenic infection. A control group ate no fermented foods but received the S. typhi Ty21a. Faecal flora analyses showed an increase in L. acidophilus and bifidobacterial counts during fermented milk intake. The specific serum IgA titre rise to S. typhi Ty21a in the test group was > 4-fold and significantly higher (P = 0.04) than in the control group. An increase in total serum IgA was also observed. These results indicate that lactic acid bacteria which can persist in the gastrointestinal tract can act as adjuvants to the humoral immune response.

The information contained herein is to the best of our knowledge true and correct and presented in good faith. It may be subject to change without further notice. To the best of our knowledge this product does not infringe Intellectual Property Rights of any third party. This information is offered solely for your consideration and verification. Copyright© Chr. Hansen A/S. All rights reserved.

Research field: Gastrointestinal Health
Research subfield: Diarrhea
Study type: Human study
Probiotic strain: B. animalis subsp. lactis BB-12 and S. thermophilus TH-4
Dosage CFU/day: Child dependent
Product formulation: Milk powder
Reference number: 0200

Saavedra, et al. Feeding of Bifidobacterium bifidum and Streptococcus thermophilus to infants in hospital for prevention of diarrhoea and shedding of rotavirus. Lancet 1994;344(8929):1046-1049

Abstract: Acute diarrhoea is a serious cause of infant morbidity and mortality, and the development of preventive measures remains an important goal. Bifidobacteria (which constitute the predominant intestinal flora of breastfed infants), as well as other lactic-acid-producing organisms such as Streptococcus thermophilus, are thought to have a protective effect against acute diarrhoeal disease. However, their efficacy has not been assessed in controlled trials. In a double-blind, placebo-controlled trial, infants aged 5-24 months who were admitted to a chronic medical care hospital were randomised to receive a standard infant formula or the same formula supplemented with Bifidobacterium bifidum and S thermophilus. Patients were evaluated daily for occurrence of diarrhoea, and faecal samples, obtained weekly, were analysed for rotavirus antigen by enzyme immunoassay. Faecal samples were also obtained during an episode of diarrhoea for virological and bacteriological analyses. 55 subjects were evaluated for a total of 4447 patient-days during 17 months. 8 (31%) of the 26 patients who received the control formula and 2 (7%) of 29 who received the supplemented formula developed diarrhoea during the course of the study (p = 0.035, Fisher's exact test, two-tailed). 10 (39%) of the subjects who received the control formula and 3 (10%) of those who received the supplemented formula shed rotavirus at some time during the study (p = 0.025). The supplementation of infant formula with B bifidum and S thermophilus can reduce the incidence of acute diarrhoea and rotavirus shedding in infants admitted to hospital.

The information contained herein is to the best of our knowledge true and correct and presented in good faith. It may be subject to change without further notice. To the best of our knowledge this product does not infringe Intellectual Property Rights of any third party. This information is offered solely for your consideration and verification. Copyright© Chr. Hansen A/S. All rights reserved.

CHR HANSEN

Research field: Gastrointestinal Health
Research subfield: Irregular bowel movements
Study type: Human study
Probiotic strain: B. animalis subsp. lactis BB-12 and L. acidophilus LA-5
Dosage CFU/day: 400 - 450 billion LA-5 and 250 - 400 billion BB-12
Product formulation: Fermented milk
Reference number: 0056

Alm, et al. Effect of a new fermented milk product "CULTURA" on constipation in geriatric patients. Horizon Scientific Press 1993

Abstract: Abstract; DESIGN: 23 elderly geriatric patients with a history of chronic constipation consumed either unfermented or fermented milk. The study ran over 4 periods of 5 - 6 weeks. RESULTS: There was a significant improvement in frequency of bowel movement (P<0.05). No negative side effects were observed, and the product appears unlikely to cause electrolyte disturbances that can follow chronic use of laxatives. Supplementation with Lactobacilli and Bifidobacteria can improve natural bowel transit time and elimination rate, and can diminish the need for laxatives in the elderly and severely immobilized patients.

The information contained herein is to the best of our knowledge true and correct and presented in good faith. It may be subject to change without further notice. To the best of our knowledge this product does not infringe Intellectual Property Rights of any third party. This information is offered solely for your consideration and verification. Copyright© Chr. Hansen A/S. All rights reserved.

CHR HANSEN

Research field: Immune Health
Research subfield: Cancer
Study type: Human study
Probiotic strain: B. animalis subsp. lactis BB-12 and L. acidophilus LA-5
Dosage CFU/day: 24 billion
Product formulation: Capsules
Reference number: 0091

Ellegaard, et al. Infection prophlaxis in neutropenic patients by oral administration of Lactobacilli. The Seventh International Symposium on Infections in the Immunocompromised host 1992

Abstract: DESIGN: In a randomized, double-blind, placebo-controlled study, the prophylactic effect on infections in 30 neutropenic patients undergoing cytoreductive chemotherapy for acute leukemia was studied. Probiotic was given for 30 days, beginning at the start of chemotherapy. RESULTS: Fever was significantly postponed from 8 to 12 days with probiotic (P=0.033). Although not statistically significant, diarrhea was slightly higher in the probiotic group. A smaller decline in serum albumin concentration was seen in the probiotic group (P=0.056), which may be a rough estimation of the severeness of constitutional symptoms. No lactobacilli were cultured from blood, indicating no risk of septicemia from the probiotic.

The information contained herein is to the best of our knowledge true and correct and presented in good faith. It may be subject to change without further notice. To the best of our knowledge this product does not infringe Intellectual Property Rights of any third party. This information is offered solely for your consideration and verification. Copyright© Chr. Hansen A/S. All rights reserved.

Research field: Gastrointestinal Health
Research subfield: Diarrhea / Microbiota
Study type: Human study
Probiotic strain: B. animalis subsp. lactis BB-12 and L. acidophilus LA-5
Dosage CFU/day: 4 billion
Product formulation: Capsules
Reference number: 0092

Black, et al. Effect of lactic acid producing bacteria on the human intestinal microflora during ampicillin treatment. Scand.J.Infect.Dis. 1991;23:247-254

Abstract: 20 healthy volunteers participated in a double blind study concerning the effect of lactic acid producing bacteria on the intestinal microflora during ampicillin treatment. 10 volunteers received 500 mg ampicillin tablets t.i.d. together with capsules containing lactic acid producing bacteria (Lactobacillus acidophilus and Bifidobacterium bifidum) for 7 days, and the other 10 volunteers were given 500 mg ampicillin tablets together with placebo capsules t.i.d. for 7 days. Both groups of volunteers continued the intake of the capsules t.i.d. for another 14 days after the ampicillin administration had been completed. The number of enterococci, streptococci and corynebacteria decreased during ampicillin administration but returned to normal levels after 14 days. Yeasts increased during the antibiotic treatment but returned to the same levels as before treatment within 14 days. Escherichia coli strains were suppressed in most volunteers during ampicillin administration. The numbers of anaerobic gram-positive cocci and rods decreased in most subjects during ampicillin treatment but were normalized within 2 weeks. Bacteroides strains were recovered in higher numbers in the lactic acid producing bacteria group compared to the placebo group. The volunteers receiving lactic acid producing bacteria were recolonized slightly faster than those having placebo. There were adverse effects observed in 3 subjects receiving ampicillin plus placebo. In the lactic acid producing bacteria group, one subject had diarrhoea on day 3 to on day 3 to day 7.

The information contained herein is to the best of our knowledge true and correct and presented in good faith. It may be subject to change without further notice. To the best of our knowledge this product does not infringe Intellectual Property Rights of any third party. This information is offered solely for your consideration and verification. Copyright© Chr. Hansen A/S. All rights reserved.

CHR HANSEN

Research field: Gastrointestinal Health
Research subfield: Diarrhea
Study type: Human study
Probiotic strain: B. animalis subsp. lactis BB-12 and L. acidophilus LA-5 and L. bulgaricus LBY-27 and S. thermophilus STY-31
Dosage CFU/day: 9 billion
Product formulation: Capsules
Reference number: 0100

Black, et al. Prophylactic efficacy of lactobacilli on traveler's diarrhea. Travel Med. 1989:333-335

Abstract: DESIGN: In a double-blind, placebo controlled study, 94 healthy tourists on a 2 week trip to Egypt consumed either placebo or probiotic capsules, starting 2 days before departure and continuing throughout the trip. RESULTS: Frequency of diarrhea was significantly reduced (P=0.019) with the probiotic. Protection rate against traveler's diarrhea was 39%.

Chr. Hansen A/S -10-12 Bøge Allé – DK-2970 Hørsholm, Denmark - Phone: +45 45 74 74 74 - Fax: +45 45 74 88 88 - www.chr-hansen.com

The information contained herein is to the best of our knowledge true and correct and presented in good faith. It may be subject to change without further notice. To the best of our knowledge this product does not infringe Intellectual Property Rights of any third party. This information is offered solely for your consideration and verification. Copyright© Chr. Hansen A/S. All rights reserved.

Research field: Gastrointestinal Health
Research subfield: Diarrhea / irregular bowel movements
Study type: Human study
Probiotic strain: B. animalis subsp. lactis BB-12 and L. acidophilus LA-5
Dosage CFU/day: 12 billion
Product formulation: Capsules
Reference number: 0250

Sagen. Treatment of functional disturbance in the intestine by administration of lactic acid bacteria. Internal report. Internal report 1989

Abstract: DESIGN: 24 partly or completely immobile, nursing home patients with various intestinal function problems were included in a double blind trial. Patients received placebo or probiotic capsules for 4 weeks. Observations were made by the nursing staff. RESULTS: Intestinal function was improved in 56% of the group receiving probiotic compared to 10% of the placebo group (P=0.019). Improvements included effect on running diarrhea and severe constipation.

The information contained herein is to the best of our knowledge true and correct and presented in good faith. It may be subject to change without further notice. To the best of our knowledge this product does not infringe Intellectual Property Rights of any third party. This information is offered solely for your consideration and verification. Copyright© Chr. Hansen A/S. All rights reserved.

Research field: Gastrointestinal Health
Research subfield: Diarrhea
Study type: Human study
Probiotic strain: B. animalis subsp. lactis BB-12 and L. acidophilus LA-5 and L. bulgaricus LBY-27 and S. thermophilus STY-31
Dosage CFU/day: 11 billion
Product formulation: Capsules
Reference number: 0101

Black, et al. Report from a placebo-controlled double-blind trial of 4 latobacilli strains (HIP) used as a prophylactic agent against travellers diarrhoea. Internal Report. Internal report 1988

Abstract: DESIGN: A placebo-controlled double-blind randomized trial including 101 danish tourists on a 15-days round trip in Egypt. Probiotic treatment/placebo was started 7 days before departure and continued until 4 days after returning from Egypt. Recordings were made of defecation frequency and characteristics of feces during treatment period. 84 subjects were included in the calculation. RESULTS: 76% of placebogroup and 51% of probiotic group experienced diarrhea during their travel (P=0.0365). The total number of days with diarrhoea in the probiotic group was significantly smaller than placebo group (P=0.025)

Chr. Hansen A/S -10-12 Bøge Allé – DK-2970 Hørsholm, Denmark - Phone: +45 45 74 74 74 - Fax: +45 45 74 88 88 - www.chr-hansen.com

The information contained herein is to the best of our knowledge true and correct and presented in good faith. It may be subject to change without further notice. To the best of our knowledge this product does not infringe Intellectual Property Rights of any third party. This information is offered solely for your consideration and verification. Copyright© Chr. Hansen A/S. All rights reserved.

CHR HANSEN

Research field: Other
Research subfield: Safety
Study type: Human study
Probiotic strain: B. animalis subsp. lactis BB-12 and L. acidophilus LA-5 and L. bulgaricus LBY-27 and S. thermophilus STY-31
Dosage CFU/day: 9 billion
Product formulation: Capsules
Reference number: 0099

Black, Laulund. A study on the tolerance in voluntary subjects to a capsule preparation of lactic acid bacteria. Internal report. Internal report 1988

Abstract: DESIGN: The present study was carried out to evaluate the tolerance of the encapsulated LAB preparation over a period of 3 weeks. 8 healthy adults received 3 capsules 3 times daily containing either skimmed milk powder (placebo) or BB-12, LA-5, STY-31 and LBY-27 alternating between 3 weeks probiotic and 2 weeks placebo. RESULTS: No changes were observed in the day to day records of defecation frequency and characteristics of faeces. No side effects were observed in reaction to the ingestion of lactic acid bacteria. In conclusion 3 weeks treatment with probiotic mix did not influence the general condition of 8 volunteers.

The information contained herein is to the best of our knowledge true and correct and presented in good faith. It may be subject to change without further notice. To the best of our knowledge this product does not infringe Intellectual Property Rights of any third party. This information is offered solely for your consideration and verification. Copyright© Chr. Hansen A/S. All rights reserved.

Research field: Gastrointestinal Health
Research subfield: Recovery / Microbiota
Study type: Human study
Probiotic strain: B. animalis subsp. lactis BB-12 and L. acidophilus LA-5
Dosage CFU/day: 12 billion
Product formulation: Capsules
Reference number: 0308

Black, Laulund. A study on the recovery of ingested, encapsulated Lactobacillus acidophilus and Bifidobacteria bifidum from doudenal fluid and faeces. Internal report 1988

Abstract: DESIGN: 4 healthy adults consumed 1 encapsulated (with an acid-resistant matrix) probiotic capsule at each meal for 1 week. Fecal samples were collected before, during and after the week ingesting capsules. Duodenal samples were taken before and periodically after ingestion. RESULTS: Both strains were found to be in the duodenum after ingestion (identification directly on agar), indicating survival through the stomach. BB-12 appears to have a greater ability than LA-5 to persist in the upper small intestine in vivo. The number of Bifidobacteria in the feces increased significantly during treatment ($P<0.01$), indicating adherence and colonization.

The information contained herein is to the best of our knowledge true and correct and presented in good faith. It may be subject to change without further notice. To the best of our knowledge this product does not infringe Intellectual Property Rights of any third party. This information is offered solely for your consideration and verification. Copyright© Chr. Hansen A/S. All rights reserved.

Research field: Gastrointestinal Health
Research subfield: Diarrhea
Study type: Human study
Probiotic strain: B. animalis subsp. lactis BB-12 and L. acidophilus LA-5 and L. bulgaricus LBY-27 and S. thermophilus STY-31
Dosage CFU/day: 9 billion
Product formulation: Capsules
Reference number: 0307

Black. Placebo-contolled double-blind trial of 4 lactobacilli strains (HIP) used as prophylactic agent against traveller's diarrhea (2 trials). Report by G.Nirnberger, Bioconsult, GmbH 1987

Abstract: DESIGN: In 2 placebo-controlled, double-blind trials, a total of 195 travelers consumed a probiotic capsule at each meal during a 15 day tour of Egypt. Treatment began 2 or 7 days before departure, and continued to the end of, or 4 days after, the trip. RESULTS: Only 46.8% of the probiotic group, compared to 73.3% of the placebo group, suffered from travellerÃ¢â‚¬â„¢s diarrhea (P<0.05). Out of the subjects who suffered from diarrhea, only 45.9% of the probiotic group, compared to 68.3% in the placebo group, experienced a duration of diarrhea for more than one day.

The information contained herein is to the best of our knowledge true and correct and presented in good faith. It may be subject to change without further notice. To the best of our knowledge this product does not infringe Intellectual Property Rights of any third party. This information is offered solely for your consideration and verification. Copyright© Chr. Hansen A/S. All rights reserved.

CHR HANSEN

Study summaries L. casei 431®

This binder provides you with summaries of selected publications on *Lactobacillus paracasei* subsp. *paracasei* L. casei 431®.

The publications are clinical studies performed in humans documenting the effects in various conditions.

October 2011

Chr. Hansen A/S

Human Health & Nutrition

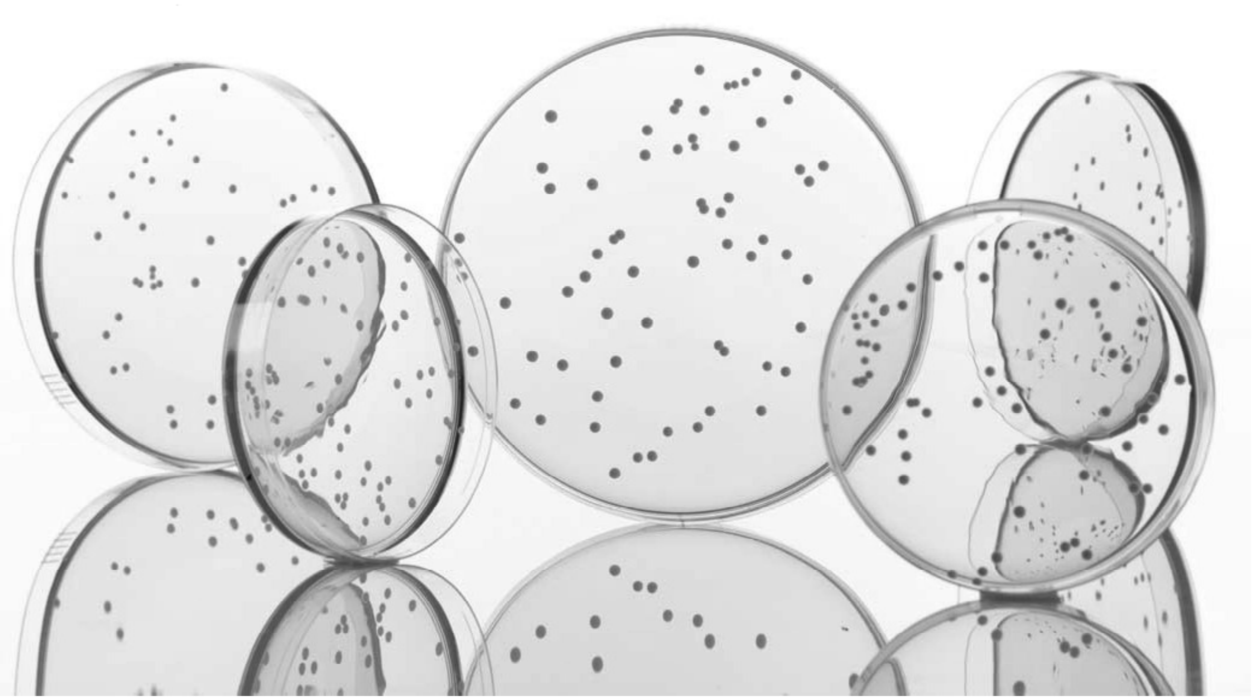

Chr. Hansen A/S -10-12 Bøge Allé – DK-2970 Hørsholm, Denmark - Phone: +45 45 74 74 74 - Fax: +45 45 74 88 88 - www.chr-hansen.com

The information contained herein is to the best of our knowledge true and correct and presented in good faith. It may be subject to change without further notice. To the best of our knowledge this product does not infringe Intellectual Property Rights of any third party. This information is offered solely for your consideration and verification. Copyright© Chr. Hansen A/S. All rights reserved.

CHR HANSEN

Research field: Immune Health
Research subfield: Immune stimulation
Study type: Human study
Probiotic strain: BB-12 and L. casei 431
Dosage CFU/day: 1 billion
Product formulation: Fermented milk (acidified milk) or capsules
Reference number: 1325

Rizzardini, et al. Evaluation of the immune benefits of two probiotic strains Bifidobacterium animalis ssp. lactis, BB-12(R) and Lactobacillus paracasei ssp. paracasei, L. casei 431(R) in an influenza vaccination model: a randomised, double-blind, placebo-controlled study Br.J.Nutr. 2011:1-9

Abstract: The present study investigated the ability of *Bifidobacterium animalis* ssp. *lactis* (BB-12®) and *Lactobacillus paracasei* ssp. *paracasei* (L. casei 431®) to modulate the immune system using a vaccination model in healthy subjects. A randomised, double-blind, placebo-controlled, parallel-group study was conducted in 211 subjects (56 % females, mean age 33·2 (sd 13·1) years). Subjects consumed a minimum of 10^9 colony-forming units of BB-12® (capsule) or L. casei 431® (dairy drink) or a matching placebo once daily for 6 weeks. After 2 weeks, a seasonal influenza vaccination was given. Plasma and saliva samples were collected at baseline and after 6 weeks for the analysis of antibodies, cytokines and innate immune parameters. Changes from baseline in vaccine-specific plasma IgG, IgG1 and IgG3 were significantly greater in both probiotic groups *v.* the corresponding placebo group (L. casei 431®, $P = 0{\cdot}01$ for IgG; $P < 0{\cdot}001$ for remaining comparisons). The number of subjects obtaining a substantial increase in specific IgG (defined as ≥ 2-fold above baseline) was significantly greater in both probiotic groups *v.* placebo (BB-12®, $P < 0{\cdot}001$ for IgG, IgG1 and IgG3; L. casei 431®, $P < 0{\cdot}001$ for IgG1 and IgG3). Significantly greater mean fold increases for vaccine-specific secretory IgA in saliva were observed in both probiotic groups *v.* placebo (BB-12®, $P = 0{\cdot}017$; L. casei 431®, $P = 0{\cdot}035$). Similar results were observed for total antibody concentrations. No differences were found for plasma cytokines or innate immune parameters. Data herein show that supplementation with BB-12® or L. casei 431® may be an effective means to improve immune function by augmenting systemic and mucosal immune responses to challenge.

The information contained herein is to the best of our knowledge true and correct and presented in good faith. It may be subject to change without further notice. To the best of our knowledge this product does not infringe Intellectual Property Rights of any third party. This information is offered solely for your consideration and verification. Copyright© Chr. Hansen A/S. All rights reserved.

Research field: Other
Research subfield: Safety
Study type: Human study
Probiotic strain: B. animalis subsp. lactis BB-12 and L. paracasei subsp. paracasei L. casei 431
Dosage CFU/day: NA
Product formulation: Milk powder
Reference number: 1253

Vlieger, et al. Tolerance and safety of Lactobacillus paracasei ssp. paracasei in combination with Bifidobacterium animalis ssp. lactis in a prebiotic-containing infant formula: a randomised controlled trial. Br.J.Nutr. 2009;102:869-875

Abstract: The addition of probiotics to infant formula has been shown to be an efficient way to increase the number of beneficial bacteria in the intestine in order to promote a gut flora resembling that of breast-fed infants. The objective of the present study was to evaluate the safety and tolerance of a combination of two probiotic strains in early infancy. A group of 126 newborns were randomised to receive a prebiotic-containing starter formula supplemented with Lactobacillus paracasei ssp. paracasei and Bifidobacterium animalis ssp. lactis or the same formula without probiotics for the first 3 months of life. A total of eighty infants continued the study until they were aged 6 months. Growth measurements were taken monthly at healthy baby clinics. Diaries were used to monitor behaviour, infections, use of antibiotics, as well as stool characteristics. Normal growth occurred in all infants and no statistically significant differences were detected between the probiotics group and the control group for gain in weight, length and head circumference. Infants in the probiotics group produced softer and more frequent stools during the first 3 months of life. No differences were found in crying and sleeping hours, number of parent-diagnosed infections, antibiotic use, visits to the general practitioner and number of adverse events. The use of a prebiotic-containing starter formula supplemented with L. paracasei ssp. paracasei and B. animalis ssp. lactis in early infancy is safe, well tolerated and has no adverse effects on growth and infant behaviour.

The information contained herein is to the best of our knowledge true and correct and presented in good faith. It may be subject to change without further notice. To the best of our knowledge this product does not infringe Intellectual Property Rights of any third party. This information is offered solely for your consideration and verification. Copyright© Chr. Hansen A/S. All rights reserved.

Research field: Immune Health
Research subfield: Immune stimulation
Study type: Human study
Probiotic strain: B. animalis subsp. lactis BB-12 and L. paracasei subsp. paracasei L. casei 431
Dosage CFU/day: 0.1 billion, 1 billion, 10 billion, 100 billion
Product formulation: Capsules
Reference number: 0501

Christensen, et al. Immunomodulating potential of supplementation with probiotics: a dose-response study in healthy young adults. FEMS Immunol.Med.Microbiol. 2006;47:380-390

Abstract: Certain probiotic microorganisms have been found beneficial in the treatment of immune-related diseases and may also affect immune function in healthy people. Intervention studies of probiotics in healthy humans are urgently required. Here, the immunomodulating potential of Bifidobacterium animalis ssp. lactis (BB-12) and Lactobacillus paracasei ssp. paracasei (CRL-431) was studied in a double-blind placebo-controlled parallel dose-response trial (n=71) based on five randomly assigned groups of young healthy adults supplemented for 3 weeks with 0, 10(8), 10(9), 10(10) and 10(11) CFU day(-1), respectively, of a mixture of BB-12 and CRL-431. No statistically significant dose-dependent effect was found for phagocytic activity in blood leukocytes, fecal immunoglobulin A (IgA) concentrations or production of interferon-gamma and interleukin-10 in blood cells. When evaluating data according to the amount of viable BB-12 recovered from faeces, the interferon-gamma production in blood cells was significantly reduced. In conclusion, no solid effect on the immune function of young healthy adults supplemented with even high doses of B. animalis ssp. lactis BB-12 and L. paracasei ssp. paracasei CRL-431 was demonstrated in this study.

The information contained herein is to the best of our knowledge true and correct and presented in good faith. It may be subject to change without further notice. To the best of our knowledge this product does not infringe Intellectual Property Rights of any third party. This information is offered solely for your consideration and verification. Copyright© Chr. Hansen A/S. All rights reserved.

Research field: Gastrointestinal Health
Research subfield: Recovery
Study type: Human study
Probiotic strain: B. animalis subsp. lactis BB-12 and L. paracasei subsp. paracasei L. casei 431
Dosage CFU/day: 0.1 billion, 1 billion, 10 billion, 100 billion
Product formulation: Capsules
Reference number: 0500

Larsen, et al. Dose-response study of probiotic bacteria Bifidobacterium animalis subsp lactis BB-12 and Lactobacillus paracasei subsp paracasei CRL-341 in healthy young adults. Eur.J.Clin.Nutr. 2006;60(11):1284-1293

Abstract: OBJECTIVE: This study was performed to investigate the dose-response effects of supplementation with Bifidobacterium animalis subsp lactis (BB-12) and Lactobacillus paracasei subsp paracasei (CRL-431) on blood lipids, recovery from feces and bowel habits. Changes of the fecal microflora was analyzed in the 10(10) CFU/day probiotic and placebo group. DESIGN: The study was designed as a randomized, placebo-controlled, double-blinded, parallel dose-response study. SUBJECTS: Healthy young adults (18-40 years) were recruited by advertising in local newspapers. Of the 75 persons enrolled, 71 (46 women, 25 men, mean age 25.6 years (range 18-40 years)) completed the study. INTERVENTION: The volunteers were randomly assigned into five groups receiving either placebo or a mixture of the two probiotics in the concentration of 10(8), 10(9), 10(10) or 10(11) CFU/day in 2 weeks run-in period, 3 weeks intervention and 2 weeks wash-out. Diary reporting bowel habits and well being (abdominal bloating, flatulence and headache) was kept for all 7 weeks and blood lipids, fecal recovery of BB-12 and CRL-431, as well as fecal microflora was tested before, immediately and 2 weeks after intervention. RESULTS: The fecal recovery of BB-12 increased significantly ($P < 0.001$) with increasing dose. In the group receiving 10(11) CFU/day BB-12 was recovered from 13 out of 15 volunteers. CRL-431 was not recovered in any of the fecal samples. Supplementation with probiotics did not change the fecal bacterial composition. A significant linear increase in fecal consistency (looser stool) with increasing probiotic dose ($P = 0.018$) was observed. No overall dose-response effect was found on the blood lipids. High doses of probiotics were well tolerated. CONCLUSION: A dose-related recovery of BB-12 from feces was observed.

The information contained herein is to the best of our knowledge true and correct and presented in good faith. It may be subject to change without further notice. To the best of our knowledge this product does not infringe Intellectual Property Rights of any third party. This information is offered solely for your consideration and verification. Copyright© Chr. Hansen A/S. All rights reserved.

Research field: Immune Health
Research subfield: Immune stimulation
Study type: Human study
Probiotic strain: L. paracasei subsp. paracasei L. casei 431 and L. rhamnosus LGG
Dosage CFU/day: 10 billion
Product formulation: Fermented milk
Reference number: 0462

de Vrese, et al. Probiotic bacteria stimulate virus-specific neutralizing antibodies following a booster polio vaccination. Eur.J.Nutr. 2005;44:406-413

Abstract: BACKGROUND: Orally ingested probiotic bacteria may modulate the immune response and increase antibody titers against enteric infections by bacteria or viruses. Even though positive effects of probiotics on respiratory tract infections have been reported, overall only few studies have examined effects on virus infections concerning organs other than the gastrointestinal tract. AIM OF THE STUDY: It was the aim of the study to investigate whether and how probiotics affect the immune response to a standardized enterovirus challenge (polio) and infections not limited to the gastrointestinal tract in healthy adults. METHODS: In a randomized, controlled and double-blind study 64 volunteers consumed for 5 weeks chemically acidified clotted milk without bacteria or with 10(10)/serving (Lactobacillus rhamnosus) GG or Lactobacillus acidophilus CRL431 added. In the second week subjects were vaccinated orally against polio 1, 2 and 3. Polio virus neutralizing serum activity, the primary parameter, was determined by the standard neutralization test (WHO) before and three times after vaccination. Polio-specific IgA, IgG and IgM were detected by ELISAs. RESULTS: Probiotics increased poliovirus neutralizing antibody titers (NT) and affected the formation of poliovirus-specific IgA and IgG in serum. The maximum increase after immunization was about 2, 2.2, or 4-fold higher, respectively, for NT, IgG or, IgA, in volunteers consuming probiotics instead of placebo. No consistent difference was noted between bacterial strains. CONCLUSIONS: Probiotics induce an immunologic response that may provide enhanced systemic protection of cells from virus infections by increasing production of virus neutralizing antibodies.

The information contained herein is to the best of our knowledge true and correct and presented in good faith. It may be subject to change without further notice. To the best of our knowledge this product does not infringe Intellectual Property Rights of any third party. This information is offered solely for your consideration and verification. Copyright© Chr. Hansen A/S. All rights reserved.

Research field: Gastrointestinal Health
Research subfield: Diarrhea
Study type: Human study
Probiotic strain: L. paracasei subsp. paracasei L. casei 431
Dosage CFU/day: 3.5 - 350 trillion
Product formulation: Fermented milk
Reference number: 0419

Gaon, et al. Effect of Lactobacillus strains and Saccharomyces boulardii on persistent diarrhea in children. Medicina 2003;63:293-298

Abstract: The efficacy of probiotics on persistent diarrhea remains uncertain. The purpose of this study was to evaluate the effect of Lactobacillus sp and Saccharomyces boulardii on persistent diarrhea in children. In a double-blind trial eighty-nine children, aged 6-24 months were randomly distributed to receive pasteurized cow milk containing 2 viable lyophilized strains Lactobacillus casei and Lactobacillus acidophillus strains CERELA, (10(10)-10(12) colony-forming units per g) (n = 30), or lyophilized S. boulardii, (10(10)-10(12) colony forming units per g) (n = 30) or pasteurized cow milk as placebo (n = 29); on each diet 175 g was given twice a day for a 5 day period. Number of depositions, duration of illness and frequency of vomiting were considered. Enteric pathogens were isolated from stools in 40% of the patients, 27% had rotavirus. Lactobacillus and S. boulardii significantly reduced the number of depositions (p

----

The information contained herein is to the best of our knowledge true and correct and presented in good faith. It may be subject to change without further notice. To the best of our knowledge this product does not infringe Intellectual Property Rights of any third party. This information is offered solely for your consideration and verification. Copyright© Chr. Hansen A/S. All rights reserved.

CHR HANSEN

Research field: Gastrointestinal Health
Research subfield: Diarrhea
Study type: Human study
Probiotic strain: L. paracasei subsp. paracasei L. casei 431
Dosage CFU/day: NA
Product formulation: Capsules
Reference number: 0407

Gaon, et al. Effect of Lactobacillus strains (L. casei and L. Acidophillus Strains cerela) on bacterial overgrowth-related chronic diarrhea. Medicina 2002;62:159-163

Abstract: Small bowel bacterial overgrowth and related diarrhea is a condition that frequently accompanies anatomic disorders, surgically created blind loops or strictures with partial small bowel obstruction and although it is often controlled with antimicrobial therapy, alternative treatment may be needed. The aim of this study was to evaluate the efficacy of an oral probiotic preparation of 2 viable lyophilized strains of lactobacilli (1.5 g each) compared with placebo. Twenty two patients with proven overgrowth and chronic diarrhea are described. In random order and double-blind fashion, 2 groups of patients received identical capsules with both Lactobacillus casei and L. acidophillus strains CERELA (12 patients) (LC) and placebo (10 patients) (P) during three consecutive periods of 7 days each followed by a similar three periods of control after withdrawal. At the end of each period the mean daily number of stools, glucose breath H2 test, and symptoms were considered. Lactobacillus were investigated in feces in both groups at day 0 (baseline), on day 21 of treatment with LC and P and on day 21 after withdrawal. Compared with P a significant reduction in mean daily number of stools was achieved with LC (p

----

The information contained herein is to the best of our knowledge true and correct and presented in good faith. It may be subject to change without further notice. To the best of our knowledge this product does not infringe Intellectual Property Rights of any third party. This information is offered solely for your consideration and verification. Copyright© Chr. Hansen A/S. All rights reserved.

Research field: Immune Health
Research subfield: Cancer
Study type: Human study
Probiotic strain: L. paracasei subsp. paracasei L. casei 431
Dosage CFU/day: 22 billion
Product formulation: Fermented milk
Reference number: 0334

Moises, et al. Lactobacillus casei and Lactobacillus acidophilus in the treatment of bladder superficial tumors: follow-up during 36 months. Third communication 1996

Abstract: DESIGN: 20 patients with superficial urinary bladder carcinoma were surgically treated, and then orally administered CRL-431 and Lactobacillus acidophilus for 8 weeks. Tumor recurrence was evaluated for 36 months. RESULTS: Treatment with milk fermented with CRL-431 and Lactobacillus acidophilus minimized tumor recurrence after surgery, and maintained or diminished the tumoral grade.Only 1 relapse was detected. No adverse side-effects, including hepatomegaly, splenomegaly or blood alterations were observed. CRL-431 and Lactobacillus acidophilus fermented milk is recommended as immunopotentiators in tumoral processes.

The information contained herein is to the best of our knowledge true and correct and presented in good faith. It may be subject to change without further notice. To the best of our knowledge this product does not infringe Intellectual Property Rights of any third party. This information is offered solely for your consideration and verification. Copyright© Chr. Hansen A/S. All rights reserved.

CHR HANSEN

Research field: Gastrointestinal Health
Research subfield: Lactose intolerance
Study type: Human study
Probiotic strain: L. paracasei subsp. paracasei L. casei 431
Dosage CFU/day: 2.4 - 240 or 4.8 - 480 billion
Product formulation: Fermented milk
Reference number: 0331

Gaon, et al. Lactose digestion by milk fermented with Lactobacillus acidophilus and Lactobacillus casei of human origin. Medicina 1995;55:237-242

Abstract: 18 persons with lactose intolerance and 12 persons without lactose intolerance consumed either milk fermented with CRL-431 and Lactobacillus acidophilus, or regular unfermented milk. Lactose absorption and orocecal transit time was measured by the breath hydrogen test during 3 hours after ingestion. In lactase deficient subjects, breath hydrogen was significantly lower with consumption of 480 ml of fermented milk compared to regular milk (P<0.008). Intestinal orocecal transit was also significantly slower with the fermented milk (P<0.001). In lactose tolerant subjects, breath hydrogen and orocecal transit were at similar levels with both milks. Clinical symptoms with 480 ml of fermented milk were significantly less frequent (P<0.08). Regular milk caused more frequencies of borborygmi (P<0.025), bloating (P<0.05), diarrhea (P<0.05) and abdominal pain (P<0.05). No significant differences were observed with consumption of 240 ml of the milks, except for borborygmi (P<0.05). Milk fermented with CRL-431 and Lactobacillus acidophilus enhances lactose digestion, influences orocecal transit time, and diminishes intolerance symptoms in lactose intolerant people.

The information contained herein is to the best of our knowledge true and correct and presented in good faith. It may be subject to change without further notice. To the best of our knowledge this product does not infringe Intellectual Property Rights of any third party. This information is offered solely for your consideration and verification. Copyright© Chr. Hansen A/S. All rights reserved.

CHR HANSEN

Research field: Gastrointestinal Health
Research subfield: Diarrhea / microbiota
Study type: Human study
Probiotic strain: L. paracasei subsp. paracasei L. casei 431
Dosage CFU/day: 10 - 100 billion
Product formulation: Fermented milk
Reference number: 0333

Gonzalez, et al. Biotherapeutic role of fermented milk. Biotherapy 1994:129-134

Abstract: Fermented milk was used as therapy in infantile diarrhoea due to post-gastroenteritis syndrome. This treatment eliminated the disease in 4.0 days (mean value, SD = 2.8; n = 13) and allowed patients to return to free feeding according to their age. The weight percentile variation during treatment with fermented milk (15 days) was higher in the patients showing 3rd degree malnutrition than in other children. Bacteriotherapy can restore faecal flora which has been lowered by diarrhoea. Our results showed that levels higher than 10(6) UFC lactobacilli/g of faeces correlated with a healthy status of the children. Clinical applications of fermented milk with a mixture of Lactobacillus casei and Lactobacillus acidophilus in the prevention of gastrointestinal disorders are possible.

The information contained herein is to the best of our knowledge true and correct and presented in good faith. It may be subject to change without further notice. To the best of our knowledge this product does not infringe Intellectual Property Rights of any third party. This information is offered solely for your consideration and verification. Copyright© Chr. Hansen A/S. All rights reserved.

Research field: Gastrointestinal Health
Research subfield: Diarrhea
Study type: Human study
Probiotic strain: L. paracasei subsp. paracasei L. casei 431
Dosage CFU/day: 0.15 - 1.5 billion
Product formulation: Fermented milk
Reference number: 0296

Gonzalez, et al. Prevention of infantile diarrhoea by fermented milk. Microbiologie - Aliments - Nutrition 1990:349-354

Abstract: Prevention of intestinal diseases through administration of lactobacilli was studied in a double-blind study with 49 apparently healthy children from populations at high risk of developing diarrhoea. Lactobacillus casei and Lactobacillus acidophilus, originally isolated from faeces of healthy children, were used to prepare cultured milk (microorganisms were added to sterile 10% reconstituted milk at final concn. of 107-108 cells/ml). Children received daily for three 15-day periods (with two 15-day periods of no supplementation between) 240 ml of skim milk supplemented with 15 ml of non-cultured or cultured milk. Of the 25 children receiving non-cultured milk, 13 (52%) developed diarrhoeal syndrome during or after the experimental periods. Stool cultures from only 2 of these children gave positive results for lactic flora (>1 Ãƒâ€” 105 c.f.u./g), while unaffected children showed faecal lactobacilli counts >106 c.f.u./g. Of the 24 children given cultured milk, only 4 (17%) developed diarrhoea and in 3 cases this was attributed to antibiotics therapy; in the 4th case stool cultures were negative for lactobacilli. Mean wt increases were 203 and 445 g for children receiving non-cultured and cultured milk resp. The use of cultured milk in prevention of diarrhoea in children of high-risk populations is suggested.

Chr. Hansen A/S -10-12 Bøge Allé – DK-2970 Hørsholm, Denmark - Phone: +45 45 74 74 74 - Fax: +45 45 74 88 88 - www.chr-hansen.com

The information contained herein is to the best of our knowledge true and correct and presented in good faith. It may be subject to change without further notice. To the best of our knowledge this product does not infringe Intellectual Property Rights of any third party. This information is offered solely for your consideration and verification. Copyright© Chr. Hansen A/S. All rights reserved.

CHR HANSEN

Research field: Gastrointestinal Health
Research subfield: Diarrhea
Study type: Human study
Probiotic strain: L. paracasei subsp. paracasei L. casei 431
Dosage CFU/day: NA
Product formulation: Fermented milk
Reference number: 0318

Oliver, et al. Consumption of lactobacilli through fermented milk and effects on children's health and nutrition. Internal report NA

Abstract: DESIGN: In a double-blind, randomized study, infants received unfermented milk (placebo) or milk fermented with CRL-431 and Lactobacillus acidophilus to evaluate the therapeutic and protective effect against infantile diarrhea. RESULTS: Significant differences between the placebo and treated groups were noted with respect to the cure and development of diarrhea, and weight gain. Results suggest that milk fermented with CRL-431 and Lactobacillus acidophilus can be used in the prevention and treatment of children's diarrhea, a disease that is still the main cause of mortality among infants between 1 month and 3 years of age in Third World countries.

Chr. Hansen A/S -10-12 Bøge Allé – DK-2970 Hørsholm, Denmark - Phone: +45 45 74 74 74 - Fax: +45 45 74 88 88 - www.chr-hansen.com

The information contained herein is to the best of our knowledge true and correct and presented in good faith. It may be subject to change without further notice. To the best of our knowledge this product does not infringe Intellectual Property Rights of any third party. This information is offered solely for your consideration and verification. Copyright© Chr. Hansen A/S. All rights reserved.

Study summaries LA-5®

This binder provides you with summaries of selected publications on *Lactobacillus acidophilus* LA-5®.

The publications are clinical studies performed in humans documenting the effects in various conditions.

October 2011

Chr. Hansen A/S

Human Health & Nutrition

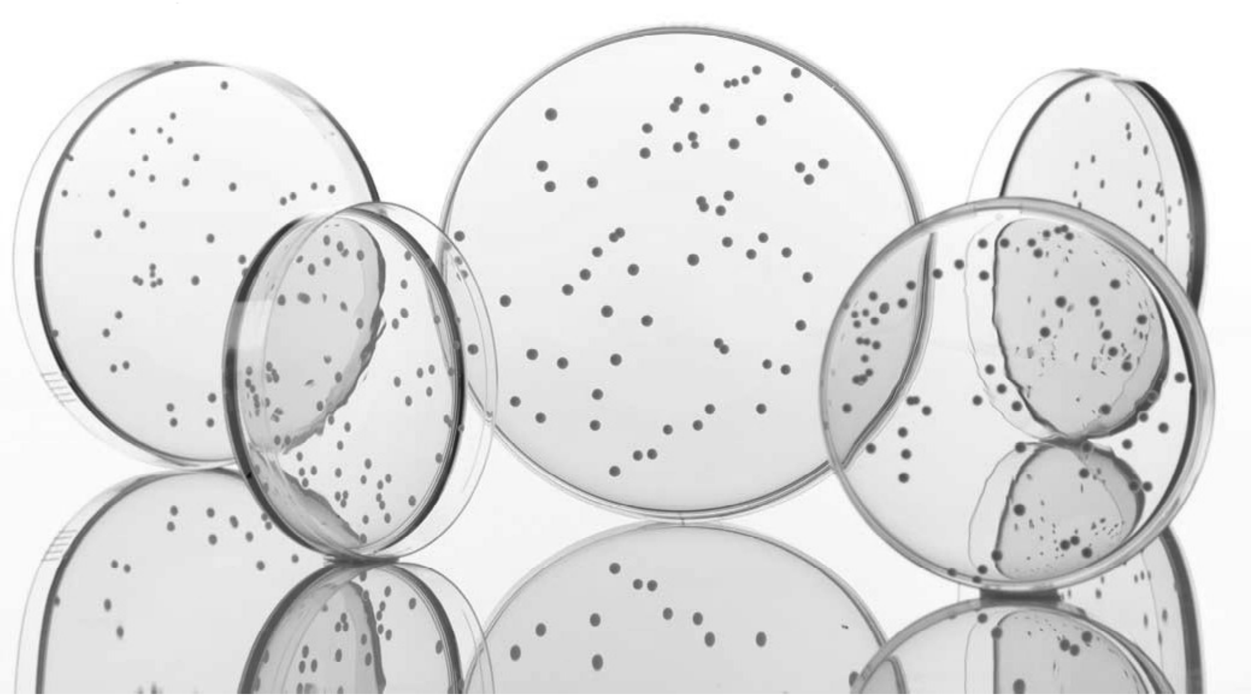

Chr. Hansen A/S -10-12 Bøge Allé – DK-2970 Hørsholm, Denmark - Phone: +45 45 74 74 74 - Fax: +45 45 74 88 88 - www.chr-hansen.com

The information contained herein is to the best of our knowledge true and correct and presented in good faith. It may be subject to change without further notice. To the best of our knowledge this product does not infringe Intellectual Property Rights of any third party. This information is offered solely for your consideration and verification. Copyright© Chr. Hansen A/S. All rights reserved.

CHR HANSEN

Research field: Gastrointestinal Health
Research subfield: Diarrhea
Study type: Human study
Probiotic strain: B. animalis subsp. lactis BB-12 and L. acidophilus LA-5
Dosage CFU/day: 4 billion
Product formulation: Capsules
Reference number: 1326

Bhalla. Randomized placebo-controlled, double blind, Multicentric Trial on Efficacy and Safety of Providac techsules (Lactobacillus acidophilus LA-5 and Bifidobacterium BB -12) for prevention of Antibiotic-Associated Diarrhea in Indian patients. ACCP 2011;Chicago, Illinois:.

Abstract: Statement of Purpose, Innovation or Hypothesis: The role of probiotics in Antibiotic Associated Diarrhoea [AAD] has not been well studied. This trial evaluated the efficacy and safety of Providac techsules in the prevention of AAD in Indian patients. **Description of Methods and Materials:** Double blind placebo controlled multicentric trial with patients of either gender who require a systemic oral antibiotic therapy for 7 days were randomized into Providac or placebo treatment, taken twice daily after food for 14 days and followed up with symptom diary card for AAD assessment. **Data and Results:** Evaluable subjects, 176 in the Providac group while 167 in the placebo group, had comparable baseline profiles. After 14 days therapy, incidence of AAD in the Providac group was 10.8% compared to 15.56% in the placebo group($p=0.19$). The duration of diarrhoea was significantly less ($p=0.010$) in the Providac group (2.32 ±2.31 days) compared to the placebo (4.58 ±3.08 days) group. Severe diarrhoea - (manifested as watery stool) was more in the Placebo group (96%) than the Providac group (31.6%) and this difference was highly significant statistically ($p<0.0001$). There were no Serious Adverse Events in any of the treatment arms and the adverse events which were observed were all non-serious, mild and self limiting. **Interpretation, Conclusion or Significance:** In Indian adult patients with AAD, Providac can effectively reduce the duration of diarrhea as well as its severity in terms of consistency of stools.

The information contained herein is to the best of our knowledge true and correct and presented in good faith. It may be subject to change without further notice. To the best of our knowledge this product does not infringe Intellectual Property Rights of any third party. This information is offered solely for your consideration and verification. Copyright© Chr. Hansen A/S. All rights reserved.

CHR HANSEN

Research field: Immune Health
Research subfield: Inflammation
Study type: Human study
Probiotic strain: B. animalis subsp. lactis BB-12 and L. acidophilus LA-5
Dosage CFU/day: 2 billion
Product formulation: Fermented milk
Reference number: 1350

Asemi, et al. Effects of daily consumption of probiotic yoghurt on inflammatory factors in pregnant women: a randomized controlled trial Pak.J.Biol.Sci. 2011;14(8):476-482

Abstract: Previous studies have shown that inflammatory factors increases in pregnancy and is associated with several complications of pregnancy. The aim of this study was to assess effects of daily consumption of probiotic yoghurt on inflammatory factors in pregnant women. In a randomized clinical trial, seventy primigravid (the first pregnancy) and singleton pregnant women aged 18-30 years were assigned to two groups. Subjects consumed daily 200 g probiotic yoghurt containing Lactobacillus acidophilus La5 and Bifidobacterium animalis BB12 (10(7) CFU g(-1) for each) or 200 g conventional yoghurt for 9 weeks. Fasting blood samples were collected at baseline (28 weeks of gestation) and after intervention (37 weeks of gestation). Inflammatory factors, hs-CRP and TNF-alpha, were measured by Enzyme-linked Immunosorbent Assay (ELISA). Independent t-test was used to compare the two groups after intervention and paired-sample t-test compared variables before and after treatment. The results showed that the probiotic yogurt brought about a decrease in the serum hs-CRP level, from 10.44 +/- 1.56 to 7.44 +/- 1.03 microg mL(-1) (p = 0.041). There was no significant change in the conventional yogurt group in the serum hs-CRP level (12.55 +/- 1.57 to 14.51 +/- 1.62 microg mL(-1), p = 0.202). The probiotic yogurt had no effect on TNF-alpha (from 73.75 +/- 6.59 to 77.91 +/- 5.61 pg mL(-1), p = 0.633). Serum TNF-alpha did not change in the conventional yogurt group (p = 0.134). In conclusion probiotic yogurt significantly decreased hs-CRP in pregnant women but had no effect on TNF-alpha.

The information contained herein is to the best of our knowledge true and correct and presented in good faith. It may be subject to change without further notice. To the best of our knowledge this product does not infringe Intellectual Property Rights of any third party. This information is offered solely for your consideration and verification. Copyright© Chr. Hansen A/S. All rights reserved.

Research field: Gastrointestinal Health
Research subfield: Diarrhea
Study type: Human study
Probiotic strain: B. animalis subsp. lactis BB-12 and L. acidophilus LA-5
Dosage CFU/day: 0.25 billion
Product formulation: Fermented milk
Reference number: 1345

de Vrese, et al. Probiotic lactobacilli and bifidobacteria in a fermented milk product with added fruit preparation reduce antibiotic associated diarrhea and Helicobacter pylori activity J.Dairy Res. 2011:1-8

Abstract: To investigate matrix-specifity of probiotic effects and particularly of the reduction of antibiotics-associated diarrhea, a controlled, randomized, double-blind study was performed, in which 88 Helicobacter pylori-infected but otherwise healthy subjects were given for eight weeks either a) a probiotic fruit yoghurt "mild" containing Lactobacillus acidophilus LA-5 plus Bifidobacterium lactis BB-12, n=30), b) the same product but pasteurized after fermentation (n=29) or c) milk acidified with lactic acid (control, n=29). During week five, a Helicobacter eradication therapy was performed. Helicobacter activity was measured via 13C-2-urea breath tests and antibiotic-associated diarrhoea and other gastrointestinal complaints were recorded by validated questionnaires. In intervention group a, b and c the mean number of days with diarrhoea was 4, 10 and 10 ($P<0{\cdot}05$), the frequency of episodes 17%, 7% and 27% (n.s.), and the change in total symptoms score before antibiotics treatment was $-1{\cdot}4\pm1{\cdot}1$, $-1{\cdot}2\pm1{\cdot}1$, $2{\cdot}6\pm1{\cdot}1$ points/four weeks ($P<0{\cdot}05$). All milk products decreased Helicobacter activity by 18 to 45% without significant differences between groups. The observed decrease in Hel. pylori activity seems to be not or not only due to probiotic bacteria but (rather) to components of acidified milk (most probably lactic acid). Fruit-yogurt-like fermented milk products with living probiotic bacteria significantly shorten the duration of antibiotics-associated diarrhoea and improve gastrointestinal complaints. Fruit yogurt-like fermented milk is a matrix suitable for probiotic bacteria.

Chr. Hansen A/S -10-12 Bøge Allé – DK-2970 Hørsholm, Denmark - Phone: +45 45 74 74 74 - Fax: +45 45 74 88 88 - www.chr-hansen.com

The information contained herein is to the best of our knowledge true and correct and presented in good faith. It may be subject to change without further notice. To the best of our knowledge this product does not infringe Intellectual Property Rights of any third party. This information is offered solely for your consideration and verification. Copyright© Chr. Hansen A/S. All rights reserved.

CHR HANSEN

Research field: CVD markers
Research subfield: Cholesterol
Study type: Human study
Probiotic strain: B. animalis subsp. lactis BB-12 and L. acidophilus LA-5
Dosage CFU/day: NA
Product formulation: Fermented milk
Reference number: 1320

Ejtahed, et al. Effect of probiotic yogurt containing Lactobacillus acidophilus and Bifidobacterium lactis on lipid profile in individuals with type 2 diabetes mellitus. J.Dairy Sci. 2011;94:3288-3294

Abstract: The purpose of this study was to investigate the effects of probiotic and conventional yogurt on the lipid profile in type 2 diabetic people. In a randomized double-blind controlled trial, 60 people (23 males and 37 females) with type 2 diabetes and low-density lipoprotein cholesterol (LDL-C) greater than 2.6 mmol/L were assigned to 2 groups. Participants consumed daily 300g of probiotic yogurt containing Lactobacillus acidophilus La5 and Bifidobacterium lactis Bb12 or 300g of conventional yogurt for 6 wk. Fasting blood samples, anthropometric measurements and 3-d, 24-h dietary recalls were collected at the baseline and at the end of the trial. Probiotic yogurt consumption caused a 4.54% decrease in total cholesterol and a 7.45% decrease in LDL-C compared with the control group. No significant changes from baseline were shown in triglyceride and high-density lipoprotein cholesterol (HDL-C) in the probiotic group. The total cholesterol:HDL-C ratio and LDL-C:HDL-C ratio as atherogenic indices significantly decreased in the probiotic group compared with the control group. Probiotic yogurt improved total cholesterol and LDL-C concentrations in type 2 diabetic people and may contribute to the improvement of cardiovascular disease risk factors.

The information contained herein is to the best of our knowledge true and correct and presented in good faith. It may be subject to change without further notice. To the best of our knowledge this product does not infringe Intellectual Property Rights of any third party. This information is offered solely for your consideration and verification. Copyright© Chr. Hansen A/S. All rights reserved.

CHR HANSEN

Research field: Gastrointestinal Health and Immune Health
Research subfield: Irregular bowel movements / Inflammation
Study type: Human study
Probiotic strain: B. animalis subsp. lactis BB-12 and L. acidophilus LA-5
Dosage CFU/day: 2.4 billion
Product formulation: Capsules
Reference number: 1294

Nova, et al. Beneficial effects of a synbiotic supplement on self-perceived gastrointestinal well-being and immunoinflammatory status of healthy adults. J.Med.Food 2011;14:79-85

Abstract: Abstract The use of synbiotics as health promoters is still poorly defined, and human intervention studies are scarce. This study was designed to evaluate the effects of a commercialized synbiotic product containing Lactobacillus acidophilus La5, Bifidobacterium animalis ssp. lactis Bb-12, Lactobacillus delbrueckii ssp. bulgaricus, Lactobacillus paracasei ssp. paracasei, Streptococcus thermophilus, and fructooligosaccharides on the self-reported gastrointestinal well-being and the immunoinflammatory status of healthy human subjects. In this randomized, double-blind, placebo-controlled study, 20 women and 16 men (25-45 years old) received either three tablets per day of the synbiotic product (2.4 x 10(9) colony-forming units/day) or placebo during 6 weeks. Gastrointestinal symptoms and bowel habits were evaluated through a self-administered questionnaire. In those subjects suffering from any kind of digestive disturbance (mild dyspepsia, flatulence, postprandial bloating, constipation, etc.), improvements in symptoms after product consumption were also evaluated. Blood lymphocyte subsets, phagocytic activity, serum C-reactive protein, ceruloplasmin, and adhesion molecules concentrations were analyzed prior and after treatment. A significant improvement in overall self-reported gastrointestinal symptoms and bowel habit was found in the synbiotic group. A marginal effect of treatment (analysis of variance P = .050) was observed with L-selectin, which showed a significant decrease in the synbiotic group (P = .019). In addition, basal L-selectin levels correlated with final intercellular adhesion molecule (ICAM)-1 levels (r = 0.468; P = .050), and basal ICAM-1 levels tended to correlate negatively with final L-selectin concentration (r = -0.457; P = .056). None of these correlations was found in the placebo group. The rest of the immunological parameters studied were not modified by the intervention. In conclusion, consumption of the synbiotic product improves self-perceived bowel habits and might facilitate a better profile of adhesion molecules in healthy adults.

Chr. Hansen A/S -10-12 Bøge Allé – DK-2970 Hørsholm, Denmark - Phone: +45 45 74 74 74 - Fax: +45 45 74 88 88 - www.chr-hansen.com

The information contained herein is to the best of our knowledge true and correct and presented in good faith. It may be subject to change without further notice. To the best of our knowledge this product does not infringe Intellectual Property Rights of any third party. This information is offered solely for your consideration and verification. Copyright© Chr. Hansen A/S. All rights reserved.

CHR HANSEN

Research field: Gastrointestinal Health
Research subfield: Microbiota
Study type: Human study
Probiotic strain: B. animalis subsp. lactis BB-12 and L. acidophilus LA-5
Dosage CFU/day: 1 billion LA-5 + 1 or 10 billion BB-12
Product formulation: Fermented milk
Reference number: 1296

Savard, et al. Impact of Bifidobacterium animalis subsp. lactis BB-12 and, Lactobacillus acidophilus LA-5-containing yoghurt, on fecal bacterial counts of healthy adults. Int.J.Food Microbiol. 2011:.

Abstract: This randomized, placebo-controlled, double blind, parallel dose-response study investigated the impact of 4-week commercial yoghurt consumption supplemented with Bifidobacterium animalis subsp. lactis (BB-12) and Lactobacillus acidophilus (LA-5) on fecal bacterial counts of healthy adults. Fifty-eight volunteers were randomly assigned to three different groups: 1. placebo (no probiotic, no starter and no green tea extract); 2. Yoptimal (10(9)cfu/100g of BB-12 and LA-5 and 40mg of green tea extract) and 3. Yoptimal-10 (10(10)cfu/100g of BB-12, 10(9)cfu/100g of LA-5 and 40mg of green tea extract). These yoghurt products also contained Lactobacillus delbrueckii subsp. bulgaricus (10(7)cfu/100g) and Streptococcus thermophilus (10(10)cfu/100g). The quantitative PCR (qPCR) results showed that there were significant increases (P=0.02) in bifidobacteria counts with the Yoptimal treatment as compared to baseline. The fecal numbers of B. animalis subsp. lactis and LA-5 significantly increased in the two probiotic treatments compared to the placebo treatment. Viable counts of fecal lactobacilli were significantly higher (P=0.05) and those of enterococci were significantly lower (P=0.04) after the intervention when compared to placebo. No significant difference was observed between treatments in volunteers' weight, waist girth, blood pressure, fasting plasma triglyceride and HDL-C concentrations, as well as cholesterol/HDL-cholesterol ratio. However, a significant increase in plasma cholesterol levels was observed in the placebo group (P=0.0018) but the levels remained stable in the two probiotic yoghurt groups. These results show that probiotic strains supplemented in the form of yoghurt remain active during gut transit and are associated with an increase in beneficial bacteria and a reduction in potentially pathogenic bacteria. This trial was registered at clinicaltrials.gov as NCT00730626.

The information contained herein is to the best of our knowledge true and correct and presented in good faith. It may be subject to change without further notice. To the best of our knowledge this product does not infringe Intellectual Property Rights of any third party. This information is offered solely for your consideration and verification. Copyright© Chr. Hansen A/S. All rights reserved.

CHR HANSEN

Research field: Other
Research subfield: Oral health
Study type: Human study
Probiotic strain: B. animalis subsp. lactis BB-12 and L. acidophilus LA-5
Dosage CFU/day: 0.1 billion
Product formulation: Other
Reference number: 1304

Singh, et al. Salivary mutans streptococci and lactobacilli modulations in young children on consumption of probiotic ice-cream containing Bifidobacterium lactis Bb12 and Lactobacillus acidophilus La5. Acta Odontol.Scand. 2011:.

Abstract: Abstract Objectives. To compare the levels of mutans streptococci and lactobacilli in saliva of school children, before and after consumption of probiotic and control ice-cream. Materials and methods. A double-blind, cross-over, placebo-controlled trial was carried out in forty, 12-14 year-old children, with no clinically detectable caries. The selected children were randomized equally into two groups I and II. Following an initial run-in period of 1 week, children in group I and II were given ice-creams 'A' and 'B', respectively, for 10 days. Being a cross-over study, the ice-creams were interchanged in the two groups after a 2-week wash-out period. Saliva samples at baseline and follow-up were assessed using Dentocult SM and Dentocult LB kits. Results. On statistical evaluation, it was seen that probiotic ice-cream brought about a statistically significant reduction (p-value = 0.003) in salivary mutans streptococci levels with no significant effect on lactobacilli levels. Conclusion. In conclusion, probiotic ice-cream containing Bifidobacterium lactis Bb-12 ATCC27536 and Lactobacillus acidophilus La-5 can reduce the levels of certain caries-associated micro-organisms in saliva.

The information contained herein is to the best of our knowledge true and correct and presented in good faith. It may be subject to change without further notice. To the best of our knowledge this product does not infringe Intellectual Property Rights of any third party. This information is offered solely for your consideration and verification. Copyright© Chr. Hansen A/S. All rights reserved.

CHR HANSEN

Research field: Immune Health
Research subfield: Atopic diseases
Study type: Human study
Probiotic strain: B. animalis subsp. lactis BB-12 and L. acidophilus LA-5 and L. rhamnosus LGG
Dosage CFU/day: 1 billion LA-5 + 10 billion BB-12 and LGG each
Product formulation: Fermented milk
Reference number: 0855

Dotterud, et al. Probiotics in pregnant women to prevent allergic disease: a randomized, double-blind trial. Br.J.Dermatol. 2010;163:.

Abstract: Summary Background Previous reports have suggested that certain probiotics given to mothers and children at risk of atopy halves the incidence of atopic dermatitis (AD) at 2 years of age. Objectives To examine if probiotics given to pregnant women in a nonselected population could prevent atopic sensitization or allergic diseases during the child's first 2 years. Methods In a randomized, double-blind trial of children from a nonselected maternal population (ClinicalTrials.gov identifier: NCT00159523), women received probiotic milk or placebo from 36 weeks of gestation to 3 months postnatally during breastfeeding. The probiotic milk contained Lactobacillus rhamnosus GG, L. acidophilus La-5 and Bifidobacterium animalis subsp. lactis Bb-12. Children with an itchy rash for more than 4 weeks were assessed for AD. At 2 years of age, all children were assessed for atopic sensitization, AD, asthma and allergic rhinoconjunctivitis. The intention-to-treat (ITT) analysis was enabled by multiple imputations. Results Four hundred and fifteen pregnant women were computer randomized. At 2 years, 138 and 140 children in the probiotic and the placebo groups, respectively, were assessed. In the ITT analysis, the odds ratio (OR) for the cumulative incidence of AD was 0.51 in the probiotic group compared with the placebo [95% confidence interval (CI) 0.30-0.87; P = 0.013]. There were no significant effects on asthma (OR 0.68, 95% CI 0.26-1.80; P = 0.437) or atopic sensitization (OR 1.52, 95% CI 0.74-3.14; P = 0.254). Conclusions Probiotics given to nonselected mothers reduced the cumulative incidence of AD, but had no effect on atopic sensitization.

The information contained herein is to the best of our knowledge true and correct and presented in good faith. It may be subject to change without further notice. To the best of our knowledge this product does not infringe Intellectual Property Rights of any third party. This information is offered solely for your consideration and verification. Copyright© Chr. Hansen A/S. All rights reserved.

CHR HANSEN

Research field: Gastrointestinal Health
Research subfield: Diarrhea
Study type: Human study
Probiotic strain: B. animalis subsp. lactis BB-12 and L. acidophilus LA-5 and L. rhamnosus LGG
Dosage CFU/day: 25 billion LGG, 2.5 billion LA-5, 25 billion BB-12
Product formulation: Fermented milk
Reference number: 0627

Wenus, et al. Prevention of antibiotic-associated diarrhoea by a fermented probiotic milk drink. Eur.J.Clin.Nutr. 2008;62:299-301

Abstract: OBJECTIVE: To study the preventive effect of a milk drink fermented with multistrain probiotics on antibiotic associated diarrhoea (AAD). DESIGN: Double-blind placebo controlled study. SETTING: University Hospital of North Norway. SUBJECTS AND METHODS: Of 853 patients treated with antibiotics, 87 met the inclusion criteria, and were randomized to ingestion of a fermented milk drink containing LGG, La-5 and Bb-12 (n=46) or placebo with heat-killed bacteria (n=41), during a period of 14 days. A diary was recorded, and stool samples were collected for microbiological analyses. RESULTS: Sixty-three patients completed the study according to the protocol; two patients (5.9%) in the treatment group and eight (27.6%) in the placebo group developed AAD (P=0.035). The relative risk of developing AAD was 0.21 (95% confidence interval: 0.05-0.93) when given probiotic milk drink. CONCLUSION: A fermented multistrain probiotic milk drink may prevent four of five cases of AAD in adult hospitalized patients. SPONSORSHIP: TINE BA, Oslo, Norway.

The information contained herein is to the best of our knowledge true and correct and presented in good faith. It may be subject to change without further notice. To the best of our knowledge this product does not infringe Intellectual Property Rights of any third party. This information is offered solely for your consideration and verification. Copyright© Chr. Hansen A/S. All rights reserved.

CHR HANSEN

Research field: Gastrointestinal Health
Research subfield: Diarrhea
Study type: Human study
Probiotic strain: B. animalis subsp. lactis BB-12 and L. acidophilus LA-5
Dosage CFU/day: > 400 billion of each bacteria
Product formulation: Fermented milk
Reference number: 0508

Sheu, et al. Pretreatment with Lactobacillus- and Bifidobacterium-containing yogurt can improve the efficacy of quadruple therapy in eradicating residual Helicobacter pylori infection after failed triple therapy. Am.J.Clin.Nutr. 2006;83:864-869

Abstract: Background: Lactobacillus- and Bifidobacterium-containing yogurt (AB-yogurt) can suppress Helicobacter pylori. Improvement of the eradication rate by quadruple therapy of residual H. pylori after failed triple therapy is needed. /// Objective: We tested whether prior treatment with AB-yogurt improved the efficacy of quadruple therapy in eradicating residual H. pylori after failed triple therapy. /// Design: One hundred thirty-eight patients in whom triple therapy failed were enrolled for a culture study of H. pylori to assess antimicrobial resistance. These patients were then randomly assigned in equal numbers to either a yogurt-plus-quadruple therapy group or a quadruple therapy-only group. The patients received 1 wk of quadruple therapy with or without a 4-wk pretreatment with AB-yogurt (400 mL/d). In the yogurt-plus-quadruple group, excessive δ13CO2/mL values of the 13C-urea breath test were collected before and every 2 wk during the 4-wk ingestion of yogurt. For both groups, a 13C-urea breath test was conducted >6 wk after the quadruple therapy to assess the outcome of residual H. pylori eradication. /// Results: For the patients in the yogurt-plus-quadruple therapy group infected with either antibiotic-sensitive or -resistant H. pylori, the excessive δ13CO2/mL values of the 13C-urea breath test were significantly decreased after the 4-wk ingestion of AB-yogurt ($P < 0.0001$). The yogurt-plus-quadruple therapy group had a higher H. pylori eradication rate than did the quadruple therapy-only group (intention-to-treat analysis: 85% compared with 71.1%, $P < 0.05$; per-protocol analysis: 90.8% compared with 76.6%, $P < 0.05$). /// Conclusion: A 4-wk pretreatment with AB-yogurt can decrease H. pylori loads despite antimicrobial resistance, thus improving the efficacy of quadruple therapy in eradicating residual H. pylori.

Chr. Hansen A/S -10-12 Bøge Allé – DK-2970 Hørsholm, Denmark - Phone: +45 45 74 74 74 - Fax: +45 45 74 88 88 - www.chr-hansen.com

The information contained herein is to the best of our knowledge true and correct and presented in good faith. It may be subject to change without further notice. To the best of our knowledge this product does not infringe Intellectual Property Rights of any third party. This information is offered solely for your consideration and verification. Copyright© Chr. Hansen A/S. All rights reserved.

Research field: Gastrointestinal Health
Research subfield: Diarrhea
Study type: Human study
Probiotic strain: B. animalis subsp. lactis BB-12 and L. acidophilus LA-5
Dosage CFU/day: 40 billion
Product formulation: Capsules
Reference number: 0499

Wildt, et al. Probiotic treatment of collagenous colitis: a randomized, double-blind, placebo-controlled trial with Lactobacillus acidophilus and Bifidobacterium animalis subsp. Lactis. Inflamm.Bowel Dis. 2006;12:395-401

Abstract: BACKGROUND: Probiotic treatment may be effective in diseases involving gut microflora and intestinal inflammation. In collagenous colitis (CC), a potential pathogenic role of the gut microflora has been proposed. The effect of probiotic treatment in CC is unknown. Our aim was to investigate the clinical effect of treatment with Lactobacillus acidophilus LA-5 and Bifidobacterium animalis subsp. lactis BB-12 (AB-Cap-10) in patients with CC. MATERIALS AND METHODS: Patients with CC and diarrhea were in a double-blind placebo-controlled study randomized (2:1) to AB-Cap-10 or placebo for 12 weeks. The primary end point was reduction in bowel frequency per week of >or=50%. Secondary end points were changes in bowel frequencies, stool consistency, stool weight, histopathology, and abdominal bloating and pain. RESULTS: Twenty-nine patients were randomized: 21 to probiotics and 8 to placebo. Reduction in bowel frequency per week of >or=50% occurred in 6 of 21 (29%) and in 1 of 8 (13%) patients receiving probiotic and placebo, respectively (P = 0.635). No differences between treatments were observed regarding the secondary end points. Post hoc analysis showed a median reduction in bowel frequency per week from 32 (range 18-84) to 23 (range 11-56; P < 0.005), a reduction in number of days with liquid stools per week from 6 days (range 0-7 days) to 1 day (range 0-7 days; P < 0.005), and an increase in number of days with solid stools per week (P < 0.05) in the AB-Cap-10 group. CONCLUSIONS: AB-Cap-10 had no significant effect on the chosen end points. Post hoc analysis demonstrated amelioration of clinical symptoms in the AB-Cap-10 group, indicating that probiotic treatment may potentially influence the disease course of CC.

Chr. Hansen A/S -10-12 Bøge Allé – DK-2970 Hørsholm, Denmark - Phone: +45 45 74 74 74 - Fax: +45 45 74 88 88 - www.chr-hansen.com

The information contained herein is to the best of our knowledge true and correct and presented in good faith. It may be subject to change without further notice. To the best of our knowledge this product does not infringe Intellectual Property Rights of any third party. This information is offered solely for your consideration and verification. Copyright© Chr. Hansen A/S. All rights reserved.

CHR HANSEN

Research field: Gastrointestinal Health
Research subfield: Other
Study type: Human study
Probiotic strain: B. animalis subsp. lactis BB-12 and L. acidophilus LA-5
Dosage CFU/day: 50 billion
Product formulation: Fermented milk
Reference number: 0473

Laake, et al. Outcome of four weeks' intervention with probiotics on symptoms and endoscopic appearance after surgical reconstruction with a J-configurated ileal-pouch-anal-anastomosis in ulcerative colitis. Scand.J.Gastroenterol. 2005;40:43-51

Abstract: OBJECTIVE: Pouchitis is a common and troublesome condition in patients operated on with ileal-pouch-anal-anastomosis (IPAA). A disturbed microecology in the pouch has been suggested as one possible explanation. In a previous double-blind, randomized, controlled study we demonstrated clinical improvement of symptoms in patients with ulcerative colitis (UC) operated on with IPAA, during intervention with live probiotic microbes Lactobacilli and Bifidobacteriae. The aim of the present study was to confirm our previous results in a much larger material, including clinical symptoms, faecal flora and endoscopic evaluation, and to compare the results in UC/IPAA patients with those of patients with familial adenomatous polyposis (FAP) with IPAA and UC patients with ileorectal anastomosis (IRA). MATERIAL AND METHODS: Five hundred millilitres of a fermented milk product (Cultura) containing live lactobacilli (La-5) and bifidobacteriae (Bb-12) was given daily for 4 weeks to 51 UC patients and 10 patients with FAP, operated on with IPAA, and six UC patients operated on for IRA. Stool samples were cultured for examination of lactobacilli, bifidobacteriae, fungi and pH before, during and after intervention. Before, during and after intervention, endoscopic evaluation was performed. Categorized symptomatology was examined prospectively using diary cards in addition to an interview, before and on the last day of intervention. RESULTS: The number of lactobacilli and bifidobacteriae increased significantly during intervention in the UC patients operated on with IPAA and remained significantly increased one week after intervention. Involuntary defecation, leakage, abdominal cramps and the need for napkins (category I), faecal number and consistency (category II) and mucus and urge to evacuate stools (category III) were significantly decreased during intervention in the UC/IPAA group. In the FAP group there was a significant decrease in faecal leakage, abdominal cramps and use of napkins (category I) during intervention. The median endoscopic score of inflammation was significantly decreased during intervention in the UC/IPAA patients. Blood tests, faecal fungi and faecal pH did not change significantly during intervention. CONCLUSIONS: Results of this extended study, showing an effect of probiotics on symptoms and endoscopic inflammation in UC patients operated on with IPAA confirm our previously reported effect of probiotics on clinical symptoms and endoscopic score in a smaller, double-blind, randomized, controlled study. The significantly higher response to probiotics in families with increased risk of IBD will have to be repeated in future studies.

The information contained herein is to the best of our knowledge true and correct and presented in good faith. It may be subject to change without further notice. To the best of our knowledge this product does not infringe Intellectual Property Rights of any third party. This information is offered solely for your consideration and verification. Copyright© Chr. Hansen A/S. All rights reserved.

CHR HANSEN

Research field: Gastrointestinal Health
Research subfield: Inflammatory bowel disease
Study type: Human study
Probiotic strain: B. animalis subsp. lactis BB-12 and L. acidophilus LA-5
Dosage CFU/day: Approx. 50 billion of each bacteria
Product formulation: Fermented milk
Reference number: 0466

Laake, et al. Assessment of mucosal inflammation and blood flow in response to four weeks' intervention with probiotics in patients operated with a J-configurated ileal-pouch-anal-anastomosis (IPAA). Scand.J.Gastroenterol. 2004;39(12):1228-1235

Abstract: BACKGROUND: Pouchitis is a common and troublesome condition in patients operated on with ileal-pouch-anal-anastomosis (IPAA). A disturbed mucosal perfusion in the pouch has been suggested as a possible cause. Laser Doppler flowmetry (LDF) has been used successfully to measure gastric and colonic mucosal perfusion in humans. In a previous study, we demonstrated a reduced mucosal perfusion in the distal part of the pouch, during probiotic intervention, examined by LDF measurement. The aim of the present study was to confirm our previous results in a much larger material, and to compare the results of LDF measurements and inflammatory activity in ulcerative colitis (UC) patients with those in familial adenomatous polyposis (FAP) patients. METHODS: Five hundred millilitres of a fermented milk product (Cultura), containing live lactobacilli (La-5) and bifidobacteria (Bb-12), was given daily for 4 weeks to 41 UC and 10 patients with FAP, operated on with IPAA. Mucosal perfusion was measured with LDF and the degree of inflammation was examined at predefined levels of the distal bowel by histology and faecal calprotectin measurements both before and after intervention. We also evaluated the applicability of a Pouchitis Disease Activity Index (PDAI). RESULTS: The LDF measurements were reproducible in the pelvic pouch at each of the predefined levels, but did not change during intervention. Mucosal perfusion was significantly reduced in the distal compared to the proximal part of the pouch in the UC group ($P < 0.05$). The perfusion levels were higher in the FAP patients compared to the UC patientsat all predefined levels ($P < 0.05$). Calprotectin levels and histological score did not change significantlyafter intervention in any of the groups. The calprotectin level was significantly lower in the FAP compared to the UC group both before and after intervention. The PDAI decreased in both groups from alevel considered diagnostic for pouchitis to a level considered as not active pouchitis. The decreasewas significant for the UC patients. CONCLUSIONS: The results did not demonstrate an effect of probiotics on histology, although a significant effect on the PDAI was achieved, which concurs with the previously reported effect on symptoms and endoscopic score. The significantly reduced blood flow in the UC group compared to the FAP group, operated on with the same procedure, and the significantly increased calprotectin levels in the UC group, are original findings. Both findings may be related to an increased risk for pouchitis among UC patients. The lack of effect of intervention on mucosal perfusion does not exclude a role for reduced circulation as a cause of pouchitis based on the reduced LDF measurements in the distal part of the pouch.

The information contained herein is to the best of our knowledge true and correct and presented in good faith. It may be subject to change without further notice. To the best of our knowledge this product does not infringe Intellectual Property Rights of any third party. This information is offered solely for your consideration and verification. Copyright© Chr. Hansen A/S. All rights reserved.

Research field: Gastrointestinal Health
Research subfield: Diarrhea
Study type: Human study
Probiotic strain: B. animalis subsp. lactis BB-12 and L. acidophilus LA-5
Dosage CFU/day: > 4.6 billion
Product formulation: Fermented milk
Reference number: 0447

Wang, et al. Effects of ingesting Lactobacillus- and Bifidobacterium-containing yogurt in subjects with colonized Helicobacter pylori. Am.J.Clin.Nutr. 2004;80:737-741

Abstract: Background: Evidence suggests that ingesting lactic acid bacteria exerts a suppressive effect on Helicobacter pylori infection in both animals and humans. Supplementing with Lactobacillus- and Bifidobacterium-containing yogurt (AB-yogurt) was shown to improve the rates of eradication of H. pylori in humans. /// Objective: We administered AB-yogurt to subjects with asymptomatic H. pylori to test whether the yogurt could inhibit H. pylori growth. /// Design: The in vitro inhibition of H. pylori growth was determined by inoculating Lactobacillus acidophilus La5 or Bifidobacterium lactis Bb12 on plates that were inoculated with H. pylori. Assessment of the viability of H. pylori was performed by the mixed culture method with La5 or Bb12. In an intervention study, 59 adult volunteers infected with H. pylori were given AB-yogurt (107 colony-forming units of both La5 and Bb12/mL) twice daily after a meal for 6 wk. Eleven subjects positive for H. pylori infection were treated with milk placebo as control subjects. H. pylori bacterial loads were determined with use of the 13C-urea breath test, which was performed before and 4 and 8 wk after the start of AB-yogurt supplementation. /// Results: Bb12 exerted an in vitro inhibitory effect against H. pylori, whereas La5 did not show an effect. Administration of AB-yogurt decreased the urease activity of H. pylori after 6 wk of therapy ($P < 0.0001$). /// Conclusion: Regular intake of yogurt containing Bb12 and La5 effectively suppressed H. pylori infection in humans.

The information contained herein is to the best of our knowledge true and correct and presented in good faith. It may be subject to change without further notice. To the best of our knowledge this product does not infringe Intellectual Property Rights of any third party. This information is offered solely for your consideration and verification. Copyright© Chr. Hansen A/S. All rights reserved.

CHR HANSEN

Research field: Gastrointestinal Health
Research subfield: Inflammatory bowel disease / Microbiota
Study type: Human study
Probiotic strain: B. animalis subsp. lactis BB-12 and L. acidophilus LA-5
Dosage CFU/day: 50 billion
Product formulation: Fermented milk
Reference number: 0420

Laake, et al. Assessment of mucosal inflammation and circulation in response to probiotics in patients operated with ileal pouch anal anastomosis for ulcerative colitis. Scand.J.Gastroenterol. 2003;38:409-414

Abstract: BACKGROUND: Pouchitis is a common and troublesome condition, and a disturbed microbiological flora and mucosal blood flow in the pouch have been suggested as possible causes. Laser Doppler flowmetry (LDF) has been used successfully to measure gastric and colonic mucosal perfusion in humans. The aim of this study was to evaluate the effect of intervention with probiotics on ileal pouch inflammation and perfusion in the pouch, assessed by endoscopy, histology, fecal calprotectin and LDF. METHODS: A fermented milk product (Cultura; 500 ml) containing live lactobacilli (La-5) and bifidobacteria (Bb-12) was given daily for 4 weeks to 10 patients operated with ileal-pouch-anal anastomosis (IPAA) for ulcerative colitis (UC). Mucosal perfusion was measured with LDF and the degree of inflammation was examined at predefined levels of the distal bowel by endoscopy and histology. Stool samples were cultured for lactobacilli and bifidobacteria and calprotectin were measured before and after intervention. RESULTS: The LDF measurements were reproducible in the pelvic pouch at each of the predefined levels, but did not change after intervention. The mucosal perfusion was reduced in the distal compared to the proximal part of the pouch. Calprotectin levels did not change significantly after intervention. The median endoscopic score for inflammation was significantly reduced by 50% after intervention, whereas the histological score did not change significantly. CONCLUSION: The results suggest that probiotics primarily act superficially, with change of gross appearance of the mucosa at endoscopy, but without significant effect on histological picture, mucosal perfusion or faecal calprotectin, during a relatively short period of 4 weeks.

Chr. Hansen A/S -10-12 Bøge Allé – DK-2970 Hørsholm, Denmark - Phone: +45 45 74 74 74 - Fax: +45 45 74 88 88 - www.chr-hansen.com

The information contained herein is to the best of our knowledge true and correct and presented in good faith. It may be subject to change without further notice. To the best of our knowledge this product does not infringe Intellectual Property Rights of any third party. This information is offered solely for your consideration and verification. Copyright© Chr. Hansen A/S. All rights reserved.

Research field: Gastrointestinal Health
Research subfield: Diarrhea
Study type: Human study
Probiotic strain: B. animalis subsp. lactis BB-12 and L. acidophilus LA-5
Dosage CFU/day: > 10 billion
Product formulation: Fermented milk
Reference number: 0412

Sheu, et al. Impact of supplement with Lactobacillus- and Bifidobacterium-containing yogurt on triple therapy for Helicobacter pylori eradication. Aliment.Pharmacol.Ther. 2002;16:1669-1675

Abstract: Aim : To test whether supplements of Lactobacillus- and Bifidobacterium-containing yogurt (AB-Yogurt) affect the success of Helicobacter pylori eradication. /// Methods : One hundred and sixty H. pylori-infected patients were randomized into a triple-plus-yogurt group or a triple-only group, receiving 1 week of triple therapy with and without supplements of AB-Yogurt, respectively. In the triple-plus-yogurt group, AB-Yogurt was continued for 4 weeks after triple therapy. Eight weeks later, patients were assessed for the success of H. pylori eradication. The stool samples of 22 randomly selected patients, 11 from each group, were provided on enrolment, at the first week and at the fifth week for evaluation of the percentage of Bifidobacterium in anaerobes. /// Results : By intention-to-treat analysis, the triple-plus-yogurt group had a higher H. pylori eradication rate than the triple-only group (91% vs. 78%, P < 0.05). The per protocol H. pylori eradication rates were similar for both groups (93.5% vs. 89%, P = N.S.). Only patients supplemented with AB-Yogurt showed restoration of the percentage of Bifidobacterium in the anaerobes of stools at the fifth week to the level in the stools on enrolment. /// Conclusions : Supplement with AB-Yogurt can improve the intention-to-treat eradication rates of H. pylori, and can restore the depletion of Bifidobacterium in stools after triple therapy.

Chr. Hansen A/S -10-12 Bøge Allé – DK-2970 Hørsholm, Denmark - Phone: +45 45 74 74 74 - Fax: +45 45 74 88 88 - www.chr-hansen.com

The information contained herein is to the best of our knowledge true and correct and presented in good faith. It may be subject to change without further notice. To the best of our knowledge this product does not infringe Intellectual Property Rights of any third party. This information is offered solely for your consideration and verification. Copyright© Chr. Hansen A/S. All rights reserved.

Research field: Gastrointestinal Health
Research subfield: Irregular bowel movements
Study type: Human study
Probiotic strain: B. animalis subsp. lactis BB-12 and L. acidophilus LA-5
Dosage CFU/day: 1 billion BB-12 and 0.3 billion LA-5
Product formulation: Fermented milk
Reference number: 0531

Shioya, et al. Effect of fermented milk containing Bifidobacterium lactis FK 120 on the fecal flora and fecal properties in healthy female volunteers. Food Health and Nutrition Research (Journal of Nutritional Food) 2000:18-18

Abstract: DESIGN: In a crossover study, 46 healthy female volunteers with the average age of 19.7 y were given 100 mL/d of fermented milk containing Bifidobacterium lactis FK 120 (BB-12, Lactobacillus acidophilus FK 205 (LA-5), Streptococcus thermophilus FK 303 (TH-3) and Sterptococcus thermophilus FK 320 (ST-20)and 100 mL/d of pure milk as placebo for up to ten days in order to examine the effect on fecal properties and defecation frequencies. The fecal microflora, pH, water content and ammonia concentration were also examined in 10 subjects (mean age 19.8 y). RESULTS: When comparing the BB-12 period with the placebo period, BB-12 was effective in increasing the fecal amount and in changing the fecal properties positively. The ratio of bifidobacteria to total bacteria was increased, and the frequency of Clostridium prefringens was decreased, as was the fecal pH. There was a tendency towards a decreased ammonia concentration and towards increased water content. It should be noted that the authors only highlight BB-12.

The information contained herein is to the best of our knowledge true and correct and presented in good faith. It may be subject to change without further notice. To the best of our knowledge this product does not infringe Intellectual Property Rights of any third party. This information is offered solely for your consideration and verification. Copyright© Chr. Hansen A/S. All rights reserved.

Research field: Gastrointestinal Health
Research subfield: Irregular bowel movements
Study type: Human study
Probiotic strain: B. animalis subsp. lactis BB-12 and L. acidophilus LA-5
Dosage CFU/day: 1 billion BB-12 and 0.3 billion LA-5 or 3 billion BB-12 and 0.9 billion LA-5
Product formulation: Fermented milk
Reference number: 0530

Shioya, et al. Effect of fermented milk containing Bifidobacterium lactis FK 120 on the fecal flora, with special reference to Bifidum species, and fecal properties in healthy volunteers. Food Health and Nutrition Research (Journal of Nutritional Food) 2000:19-32

Abstract: DESIGN: The effects of Bifidobacterium lactis FK 120 (BB-12), Lactobacillus acidophilus FK 320 (LA-5), Streptococcus thermophilus FK 303 (TH-3) and Streptococcus thermophilus FK 320 (ST20) on fecal properties and defecation frequency were examined in 48 healthy, volunteers with the average age of 42.0 y, who were given 100 mL/d or 300 mL/d of fermented milk containing BB-12 for two weeks. Additionally, the effect of BB-12 on the composition of Bifidobacterium species in fecal microflora and on fecal properties were examined in 7 volunteers with the average age of 45.7 y. RESULTS: The intake of fermented milk containing BB-12 improved the fecal flora without a change in the composition of Bifidobacterium species. During the intake period, increase in defecation frequency and fecal amount, and improvement of fecal shape and coloring were observed. The ratio of Bifidobacteria to total bacteria was increased by the intake. The frequency of C. perfringes and Clostridium-others as well as fecal ammonia ($P<0.01$) was decreased by the intake. BB-12 was detected in the feces of four volunteers during the BB-12 administration with 107.3-8.8 cfu/g. In the composition of fecal Bifidobacterium species, the high incidence if B. adolescentis and B. longum were observed throughout the experimental periods. It should be noted that the authors only highlight BB-12.

The information contained herein is to the best of our knowledge true and correct and presented in good faith. It may be subject to change without further notice. To the best of our knowledge this product does not infringe Intellectual Property Rights of any third party. This information is offered solely for your consideration and verification. Copyright© Chr. Hansen A/S. All rights reserved.

CHR HANSEN

Research field: Gastrointestinal Health
Research subfield: Irregular bowel movements
Study type: Human study
Probiotic strain: B. animalis subsp. lactis BB-12 and L. acidophilus LA-5
Dosage CFU/day: 1 billion BB-12 and 0.3 billion LA-5
Product formulation: Fermented milk
Reference number: 0532

Shioya, et al. Effect of fermented milk containing Bifidobacterium lactis FK 120 on the fecal flora, with special reference to Bifidum species, and fecal properties in elderly volunteers. Food Health and Nutrition Research (Journal of Nutritional Food) 2000:33-44

Abstract: DESIGN: Six elderly volunteers with the average age of 81.7 y were given 100 mL/d of fermented milk containing Bifidobacterium lactis FK 120 (BB-12), Lactobacillus acidophilus FK 205 (LA-5), Streptococcus thermophilus FK 303 (TH-3) and Streptococcus thermophilus FK 320 (ST-20) for 2 weeks in order to examine the effect of BB-12 on fecal flora and fecal properties. RESULTS: BB-12 was detected within the range of 10^6.6-8.9 cfu/g in the feces of five of the six volunteers. Although a high incidence of B. adolescentis was detected before the intake, BB-12 was predominant during the intervention period. The ratio of Bifidobacteria to fecal bacteria was significantly increased (P<0.05), and the number of Clostridium perfringens and Clostridium-others was significantly decreased (P<0.05). Furthermore, an improvement of diarrhea with decreased fecal water, and a significant (P<0.05) decrease of fecal odor was observed. It should be noted that the authors only highlight BB-12.

The information contained herein is to the best of our knowledge true and correct and presented in good faith. It may be subject to change without further notice. To the best of our knowledge this product does not infringe Intellectual Property Rights of any third party. This information is offered solely for your consideration and verification. Copyright© Chr. Hansen A/S. All rights reserved.

CHR HANSEN

Research field: Gastrointestinal Health
Research subfield: Inflammatory bowel disease / Irregular bowel movements / Microbiota
Study type: Human study
Probiotic strain: B. animalis subsp. lactis BB-12 and L. acidophilus LA-5
Dosage CFU/day: > 50 billion or < 5 million (heat-treated)
Product formulation: Fermented milk
Reference number: 0383

Laake, et al. Influence of fermented milk on clinical state, fecal bacterial counts and biochemical characteristics in patients with ileal- pouch- anal-anastomosis. Microb.Ecol.Health Dis. 1999;11:211-217

Abstract: Pouchitis is a condition with acute or chronic inflammation in the ileal pouch of colectomized patients. A disturbed microecology in the pouch has been suggested as a possible cause. The present study was an attempt to investigate if a fermented milk product containing live lactobacilli and bifidobacteria might influence on the intestinal microecology of IPAA-patients. Five hundred ml of a fermented milk product containing live lactobacilli (La-5) and bifidobacteriae (Bb-12) (Cultura, TM), was given in its regular form or heat-treated, in a randomized, double blinded fashion, to 16 patients operated on with ileal- pouch- anal-anastomosis (IPAA) for ulcerative colitis (UC). Symptoms recordings, stool cultures and determination of 6 different microflora associated characteristics (MACs);(short-chain fatty acids (SCFAs), urobilinogen, mucin degradation, coprostanol, fecal tryptic activity (FTA), and beta-aspartyl glycin) were performed at inclusion, after 1 and 2 weeks. In the group given regular Cultura, daily stool frequency was reduced from median 5.8 to 2.8. It was unchanged in the group given heat-treated Cultura. With regular Cultura the number of lactobacilli increased significantly during intervention, and remained elevated one week thereafter. The number of bifidobacteriae increased significantly during intervention, but was back to baseline one week thereafter. With heat-treated Cultura, no significant change was observed. Significant reduction in stool frequency was observed among the patients given regular Cultura. No change was observed among those given heat-treated Cultura. No major effects on six MACs were observed.

Chr. Hansen A/S -10-12 Bøge Allé – DK-2970 Hørsholm, Denmark - Phone: +45 45 74 74 74 - Fax: +45 45 74 88 88 - www.chr-hansen.com

The information contained herein is to the best of our knowledge true and correct and presented in good faith. It may be subject to change without further notice. To the best of our knowledge this product does not infringe Intellectual Property Rights of any third party. This information is offered solely for your consideration and verification. Copyright© Chr. Hansen A/S. All rights reserved.

Research field: Gastrointestinal Health
Research subfield: Irregular bowel movements
Study type: Human study
Probiotic strain: B. animalis subsp. lactis BB-12 and L. acidophilus LA-5
Dosage CFU/day: 400 - 450 billion LA-5 and 250 - 400 billion BB-12
Product formulation: Fermented milk
Reference number: 0056

Alm, et al. Effect of a new fermented milk product "CULTURA" on constipation in geriatric patients. Horizon Scientific Press 1993

Abstract: Abstract; DESIGN: 23 elderly geriatric patients with a history of chronic constipation consumed either unfermented or fermented milk. The study ran over 4 periods of 5 - 6 weeks. RESULTS: There was a significant improvement in frequency of bowel movement (P<0.05). No negative side effects were observed, and the product appears unlikely to cause electrolyte disturbances that can follow chronic use of laxatives. Supplementation with Lactobacilli and Bifidobacteria can improve natural bowel transit time and elimination rate, and can diminish the need for laxatives in the elderly and severely immobilized patients.

The information contained herein is to the best of our knowledge true and correct and presented in good faith. It may be subject to change without further notice. To the best of our knowledge this product does not infringe Intellectual Property Rights of any third party. This information is offered solely for your consideration and verification. Copyright© Chr. Hansen A/S. All rights reserved.

CHR HANSEN

Research field: Women's Health
Research subfield: Yeast vaginitis
Study type: Human study
Probiotic strain: L. acidophilus LA-5
Dosage CFU/day: > 23 billion
Product formulation: Fermented milk
Reference number: 0151

Hilton, et al. Ingestion of Yogurt Containing Lactobacillus acidophilus as Prophylaxis for Candidal Vaginitis. Annals of Internal Medicine 1992;116:353-357

Abstract: *Objective:* To assess whether daily ingestion of yogurt containing *Lactobacillus acidophilus* prevents vulvovaginal candidal infections. ▪ *Design:* Crossover trial for at least 1 year during which patients were examined for candidal infections and colonizations while receiving either a yogurt-free or a yogurt-containing diet. Patients served as their own controls. ▪ *Setting:* Ambulatory infectious disease center in a teaching hospital providing tertiary care. ▪ *Patients:* Thirty-three women with recurrent candidal vaginitis were eligible after recruitment from community practices and clinics and through advertising. Twelve patients were eliminated for protocol violations. Of the remaining 21 patients, 8 who were assigned to the yogurt arm initially refused to enter the control phase 6 months later. Thus, 13 patients completed the protocol. ▪ *Interventions:* Women ate yogurt for 6 months of the study period. ▪ *Measurements:* Colonization of lactobacilli and Candida in the vagina and rectum; candidal infections of the vagina. ▪ *Main Results:* Thirty-three eligible patients were studied. A threefold decrease in infections was seen when patients consumed yogurt containing *Lactobacillus acidophilus*. The mean (± SD) number of infections per 6 months was 2.54 ± 1.66 in the control arm and 0.38 ± 0.51 per 6 months in the yogurt arm (P = 0.001). Candidal colonization decreased from a mean of 3.23 ± 2.17 per 6 months in the control arm to 0.84 ± 0.90 per 6 months in the yogurt arm (P = 0.001). ▪ *Conclusion:* Daily ingestion of 8 ounces of yogurt containing *Lactobacillus acidophilus* decreased both candidal colonization and infection.

Chr. Hansen A/S -10-12 Bøge Allé – DK-2970 Hørsholm, Denmark - Phone: +45 45 74 74 74 - Fax: +45 45 74 88 88 - www.chr-hansen.com

The information contained herein is to the best of our knowledge true and correct and presented in good faith. It may be subject to change without further notice. To the best of our knowledge this product does not infringe Intellectual Property Rights of any third party. This information is offered solely for your consideration and verification. Copyright© Chr. Hansen A/S. All rights reserved.

Research field: Gastrointestinal Health
Research subfield: Diarrhea / Microbiota
Study type: Human study
Probiotic strain: B. animalis subsp. lactis BB-12 and L. acidophilus LA-5
Dosage CFU/day: 4 billion
Product formulation: Capsules
Reference number: 0092

Black, et al. Effect of lactic acid producing bacteria on the human intestinal microflora during ampicillin treatment. Scand.J.Infect.Dis. 1991;23:247-254

Abstract: 20 healthy volunteers participated in a double blind study concerning the effect of lactic acid producing bacteria on the intestinal microflora during ampicillin treatment. 10 volunteers received 500 mg ampicillin tablets t.i.d. together with capsules containing lactic acid producing bacteria (Lactobacillus acidophilus and Bifidobacterium bifidum) for 7 days, and the other 10 volunteers were given 500 mg ampicillin tablets together with placebo capsules t.i.d. for 7 days. Both groups of volunteers continued the intake of the capsules t.i.d. for another 14 days after the ampicillin administration had been completed. The number of enterococci, streptococci and corynebacteria decreased during ampicillin administration but returned to normal levels after 14 days. Yeasts increased during the antibiotic treatment but returned to the same levels as before treatment within 14 days. Escherichia coli strains were suppressed in most volunteers during ampicillin administration. The numbers of anaerobic gram-positive cocci and rods decreased in most subjects during ampicillin treatment but were normalized within 2 weeks. Bacteroides strains were recovered in higher numbers in the lactic acid producing bacteria group compared to the placebo group. The volunteers receiving lactic acid producing bacteria were recolonized slightly faster than those having placebo. There were adverse effects observed in 3 subjects receiving ampicillin plus placebo. In the lactic acid producing bacteria group, one subject had diarrhoea on day 3 to on day 3 to day 7.

The information contained herein is to the best of our knowledge true and correct and presented in good faith. It may be subject to change without further notice. To the best of our knowledge this product does not infringe Intellectual Property Rights of any third party. This information is offered solely for your consideration and verification. Copyright© Chr. Hansen A/S. All rights reserved.

CHR HANSEN

Research field: Gastrointestinal Health
Research subfield: Lactose intolerance
Study type: Human study
Probiotic strain: L. acidophilus LA-5
Dosage CFU/day: 4 - 40 billion
Product formulation: Other
Reference number: 0115

Lin, et al. Influence of nonfermented dairy products containing bacterial starter cultures on lactose maldigestion in humans. J.Dairy Sci. 1991;74:87-95

Abstract: The effect of nonfermented dairy products containing yogurt or acidophilus cultures on lactose utilization by lactose-maldigesting humans was investigated. Yogurt and acidophilus milk containing 10(7) or 10(8) of Streptococcus thermophilus and Lactobacillus bulgaricus, or Lactobacillus acidophilus, respectively, were prepared using commercially processed 2% low fat milk. Immediately following inoculation, products were refrigerated. Lactose maldigestion was monitored by measuring breath hydrogen excretion at hourly intervals for 8 h following consumption of 400 ml of each test meal containing approximately 20 g of lactose. The yogurt milk containing 10(8) cfu/ml was shown to contain significant concentrations of microbial beta-galactosidase (EC 3.2.1.23; approximately 3 U/ml), which remained stable for at least 14 d at refrigerator temperatures. Breath hydrogen peaks were delayed and significantly lower (approximately 20 ppm at 5 to 7 h) than control values (approximately 70 ppm at 4 h), and intolerance symptoms were eliminated in all subjects. Yogurt milk containing 10(7) cfu/ml demonstrated intermediate breath hydrogen values and was marginally significantly different from control values. Lactobacillus acidophilus strains with varying resistance to bile and total beta-galactosidase-producing potential were also tested. Only one strain, LA-1, which demonstrated low bile resistance and intermediate beta-galactosidase activity, was capable of significantly decreasing breath hydrogen values when 10(8) cfu/ml of milk was consumed.

The information contained herein is to the best of our knowledge true and correct and presented in good faith. It may be subject to change without further notice. To the best of our knowledge this product does not infringe Intellectual Property Rights of any third party. This information is offered solely for your consideration and verification. Copyright© Chr. Hansen A/S. All rights reserved.

Research field: Gastrointestinal Health
Research subfield: Diarrhea / Irregular bowel movements
Study type: Human study
Probiotic strain: B. animalis subsp. lactis BB-12 and L. acidophilus LA-5
Dosage CFU/day: 12 billion
Product formulation: Capsules
Reference number: 0250

Sagen. Treatment of functional disturbance in the intestine by administration of lactic acid bacteria. Internal report. Internal report 1989:.

Abstract: DESIGN: 24 partly or completely immobile, nursing home patients with various intestinal function problems were included in a double blind trial. Patients received placebo or probiotic capsules for 4 weeks. Observations were made by the nursing staff. RESULTS: Intestinal function was improved in 56% of the group receiving probiotic compared to 10% of the placebo group (P=0.019). Improvements included effect on running diarrhea and severe constipation.

Chr. Hansen A/S -10-12 Bøge Allé – DK-2970 Hørsholm, Denmark - Phone: +45 45 74 74 74 - Fax: +45 45 74 88 88 - www.chr-hansen.com

The information contained herein is to the best of our knowledge true and correct and presented in good faith. It may be subject to change without further notice. To the best of our knowledge this product does not infringe Intellectual Property Rights of any third party. This information is offered solely for your consideration and verification. Copyright© Chr. Hansen A/S. All rights reserved.

CHR HANSEN

Research field: Gastrointestinal Health
Research subfield: Recovery / Microbiota
Study type: Human study
Probiotic strain: B. animalis subsp. lactis BB-12 and L. acidophilus LA-5
Dosage CFU/day: 12 billion
Product formulation: Capsules
Reference number: 0308

Black, Laulund. A study on the recovery of ingested, encapsulated Lactobacillus acidophilus and Bifidobacteria bifidum from doudenal fluid and faeces. Internal report 1988:.

Abstract: DESIGN: 4 healthy adults consumed 1 encapsulated (with an acid-resistant matrix) probiotic capsule at each meal for 1 week. Fecal samples were collected before, during and after the week ingesting capsules. Duodenal samples were taken before and periodically after ingestion. RESULTS: Both strains were found to be in the duodenum after ingestion (identification directly on agar), indicating survival through the stomach. BB-12 appears to have a greater ability than LA-5 to persist in the upper small intestine in vivo. The number of Bifidobacteria in the feces increased significantly during treatment ($P<0.01$), indicating adherence and colonization.

The information contained herein is to the best of our knowledge true and correct and presented in good faith. It may be subject to change without further notice. To the best of our knowledge this product does not infringe Intellectual Property Rights of any third party. This information is offered solely for your consideration and verification. Copyright© Chr. Hansen A/S. All rights reserved.
