# Supplementary material for: The evaluation of the effect of probiotics on the healing of equine distal limb wounds
Source: PLoS One. 2020 Jul 29;15(7):e0236761. doi: 10.1371/journal.pone.0236761 (PMC7390451; doi:10.1371/journal.pone.0236761)
Supplement: S1 Table — Number of wounds with presence of bacterial species at day 0, 3, and 9 per treatment group. (DOCX) [file pone.0236761.s003.docx]

**S1 Table.** **Presence of bacterial species in the wounds.**

|  | **Control** | | | **Probiotic** | | |
| --- | --- | --- | --- | --- | --- | --- |
| **Bacterial species** | **D0** | **D3** | **D9** | **D0** | **D3** | **D9** |
| Aeromonas sp. | 1 |  |  |  |  |  |
| Alcaligenes faecalis | 1 |  |  |  |  |  |
| Citrobacter koseri |  |  |  |  |  | 1 |
| Corynebacterium ulcerans |  |  |  | 1 |  |  |
| Enterobacter cloacae |  | 1 | 1 |  |  |  |
| Enterobacter sp. | 2 |  |  | 1 | 1 | 1 |
| Enterokokker | 1 | 1 |  |  |  |  |
| Escherichia coli | 4 | 3 | 7 | 3 | 3 | 2 |
| Escherichia coli with haemolytic properties |  | 1 |  |  |  | 1 |
| Klebsiella pneumoniae |  | 1 | 2 |  |  |  |
| Neisseria sp. |  | 1 |  |  |  |  |
| Proteus mirabilis | 7 | 4 | 9 | 3 | 3 | 3 |
| Pseudomonas sp. |  |  |  | 1 | 1 |  |
| Rahnella aquatilis | 1 | 1 |  | 1 |  |  |
| Serratia marcescens | 1 |  | 1 |  |  |  |
| Staphylococcus aureus | 5 | 4 | 2 | 4 | 4 | 2 |
| Staplylococcus intermedium |  |  | 1 |  |  |  |
| Staphylococcus epidermidis (physiological flora) |  | 1 |  |  |  |  |
| Staphylococcus pseudintermedius | 1 | 1 |  | 3 | 1 |  |
| Streptococcus agalactiae |  |  |  | 1 | 1 | 1 |
| Streptococcus e. zooepidemicus | 6 | 7 | 3 | 7 | 8 | 3 |
| α-haemolysis Streptococcus (physio-logical flora) |  |  |  |  | 1 |  |
| β-haemolysing Streptococcus | 3 |  | 2 | 2 | 3 | 3 |
| Pseudomonas aeruginosa | 1 |  |  | 2 |  |  |
| Staplylococcus haemolyticus |  |  |  |  |  | 2 |
| Myroides sp. |  | 1 |  |  |  |  |
| Morganella morganii (Proteus organii) |  | 1 |  |  |  |  |
| Total | 34 | 30 | 29 | 28 | 24 | 19 |

Number of wounds with presence of bacterial species at day 0, 3, and 9 per treatment group
